# LRP8‐mediated selenocysteine uptake is a targetable vulnerability in MYCN‐amplified neuroblastoma

**DOI:** 10.15252/emmm.202318014

**Published:** 2023-07-12

**Authors:** Hamed Alborzinia, Zhiyi Chen, Umut Yildiz, Florencio Porto Freitas, Felix C E Vogel, Julianna Patricia Varga, Jasmin Batani, Christoph Bartenhagen, Werner Schmitz, Gabriele Büchel, Bernhard Michalke, Jashuo Zheng, Svenja Meierjohann, Enrico Girardi, Elisa Espinet, Andrés F Flórez, Ancely Ferreira dos Santos, Nesrine Aroua, Tasneem Cheytan, Julie Haenlin, Lisa Schlicker, Thamara N Xavier da Silva, Adriana Przybylla, Petra Zeisberger, Giulio Superti‐Furga, Martin Eilers, Marcus Conrad, Marietta Fabiano, Ulrich Schweizer, Matthias Fischer, Almut Schulze, Andreas Trumpp, José Pedro Friedmann Angeli

**Affiliations:** ^1^ Heidelberg Institute for Stem Cell Technology and Experimental Medicine (HI‐STEM GmbH) Heidelberg Germany; ^2^ Division of Stem Cells and Cancer German Cancer Research Center (DKFZ) Heidelberg Germany; ^3^ Rudolf Virchow Zentrum (RVZ), Center for Integrative and Translational Bioimaging University of Würzburg Würzburg Germany; ^4^ European Molecular Biology Laboratory, Genome Biology Unit Heidelberg Germany; ^5^ Division of Tumor Metabolism and Microenvironment German Cancer Research Center (DKFZ) Heidelberg Germany; ^6^ European Molecular Biology Organization Heidelberg Germany; ^7^ Center for Molecular Medicine Cologne (CMMC) and Department of Experimental Pediatric Oncology, University Children's Hospital, Medical Faculty University of Cologne Cologne Germany; ^8^ Department of Biochemistry and Molecular Biology, Theodor Boveri Institute, Biocenter University of Würzburg Würzburg Germany; ^9^ Mildred Scheel Early Career Center University Hospital Würzburg Würzburg Germany; ^10^ Research Unit Analytical BioGeoChemistry Helmholtz Center München (HMGU) Neuherberg Germany; ^11^ Institute of Metabolism and Cell Death Helmholtz Zentrum München (HMGU) Neuherberg Germany; ^12^ Department of Pathology University of Würzburg Würzburg Germany; ^13^ CeMM‐Research Center for Molecular Medicine of the Austrian Academy of Sciences Vienna Austria; ^14^ Solgate GmbH Klosterneuburg Austria; ^15^ Anatomy Unit, Department of Pathology and Experimental Therapy, School of Medicine University of Barcelona (UB), L'Hospitalet de Llobregat Barcelona Spain; ^16^ Molecular Mechanisms and Experimental Therapy in Oncology Program (Oncobell) Institut d'Investigació Biomèdica de Bellvitge (IDIBELL), L'Hospitalet de Llobregat Barcelona Spain; ^17^ Department of Molecular and Cellular Biology Harvard University Cambridge MA USA; ^18^ Center for Physiology and Pharmacology Medical University of Vienna Vienna Austria; ^19^ Institut für Biochemie und Molekularbiologie, Rheinische Friedrich‐Wilhelms‐Universität Bonn Bonn Germany

**Keywords:** ferroptosis, neuroblastoma, selenocysteine, selenoprotein, synthetic lethality, Autophagy & Cell Death, Cancer, Neuroscience

## Abstract

Ferroptosis has emerged as an attractive strategy in cancer therapy. Understanding the operational networks regulating ferroptosis may unravel vulnerabilities that could be harnessed for therapeutic benefit. Using CRISPR‐activation screens in ferroptosis hypersensitive cells, we identify the selenoprotein P (SELENOP) receptor, LRP8, as a key determinant protecting *MYCN*‐amplified neuroblastoma cells from ferroptosis. Genetic deletion of *LRP8* leads to ferroptosis as a result of an insufficient supply of selenocysteine, which is required for the translation of the antiferroptotic selenoprotein GPX4. This dependency is caused by low expression of alternative selenium uptake pathways such as system Xc^−^. The identification of LRP8 as a specific vulnerability of *MYCN*‐amplified neuroblastoma cells was confirmed in constitutive and inducible *LRP8* knockout orthotopic xenografts. These findings disclose a yet‐unaccounted mechanism of selective ferroptosis induction that might be explored as a therapeutic strategy for high‐risk neuroblastoma and potentially other *MYCN*‐amplified entities.

## Graphical abstract

The low‐density lipoprotein receptor (LRP8) was identified as a critical suppressor of ferroptosis in MYCN‐amplified neuroblastoma. Blocking selenium/selenocysteine uptake mechanisms via LRP8 offers a selective strategy to induce ferroptosis and disrupt GPX4 function.

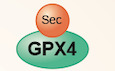

**The paper explained**

**Problem**

Ferroptosis, a distinct form of cell death, has emerged as a potential approach for eliminating solid tumors that are refractory to treatment. However, inhibiting the selenoprotein glutathione peroxidase 4 (GPX4), which suppresses ferroptosis, remains challenging due to the absence of suitable inhibitors and to the potential systemic toxicity. Our previous findings have demonstrated a notable dependence on GPX4 in high‐risk MYCN‐amplified neuroblastomas, yet the precise factors contributing to this phenomenon remain incompletely understood.

**Results**

Our study reveals that high‐risk MYCN‐amplified neuroblastomas exhibit a significant dependency on LRP8. Through genome‐wide and single‐cell transcriptomics CRISPR‐activation screens, we identify the low‐density lipoprotein receptor‐related protein 8 (LRP8) as a critical factor in selenium/selenocysteine metabolism in MYCN‐amplified cancers. We find that MYCN‐amplified cells ineficiently activate alternative selenium/selenocysteine pathways, resulting in vulnerability to LRP8 inhibition. These metabolic vulnerabilities provide a unique opportunity to target LRP8 to induce ferroptosis selectively and safely.

**Impact**

In light of the limited success in repurposing adult oncology drugs for neuroblastoma treatment, our research presents a significant advance by introducing innovative strategies based on ferroptosis. By specifically focusing on targeting LRP8, we have identified a potential breakthrough that not only offers novel therapeutic approaches for neuroblastoma but also holds promise for a range of pediatric malignancies and MYCN‐driven cancers.

## Introduction

**Figure 1:**
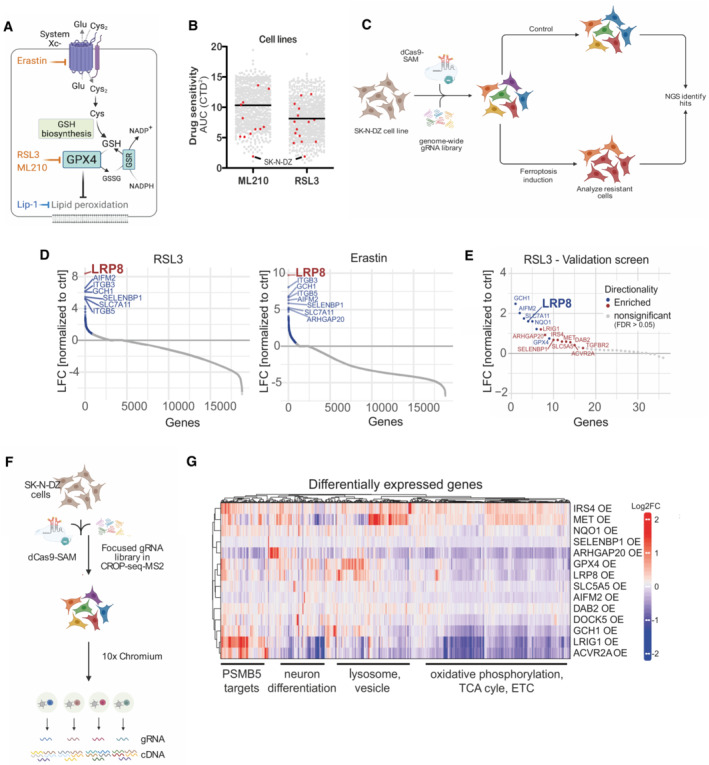
Genome‐wide CRISPR activation screen identifies negative regulators of ferroptosis Schematic depiction of ferroptosis regulators and small molecule modulators. Inhibitors are shown in light blue, and inducers in orange.Analyzis of the depmap portal (www.depmap.org) reveals *MYCN*‐amplified SK‐N‐DZ as hypersensitive cell lines to the GPX4 inhibitors RSL3 and ML210.Strategy of the genome‐wide CRISPR activation (CRISPRa) screen in *MYCN*‐amplified SK‐N‐DZ cells.Overexpression phenotypes conferring resistance to 300 nM RSL3 (left) or 1 μM Erastin (right) treatment. Significant hits are marked in blue (FDR ≤ 0.05), while the highest‐scoring hit, LRP8, is highlighted in red.Overexpression phenotypes conferring resistance to RSL3 (100 nM) induced ferroptosis in the pooled validation CRISPRa screen. Significantly enriched hits (FDR ≤ 0.05) are marked and labeled in blue (known ferroptosis regulators) or red (novel regulators identified in this study). The highest‐scoring hit from the primary screens, LRP8, is highlighted.Strategy of the single‐cell CRISPRa screen to characterize hits from the ferroptosis‐resistance screen. Guide RNA labels are recovered alongside the whole transcriptome readout for each cell.Transcriptomic consequences of CRISPRa of 14 scoring hits from the primary and the validation screens. Each row represents one CRISPRa cluster. For each cluster, the top 50 genes with the most significant differential expression (compared to the nontargeting control cluster) were selected and merged to a signature gene list represented by the columns. Columns and rows were hierarchically clustered based on Pearson's correlation. Source data are available online for this figure.

Ferroptosis is a cell death modality that is attracting increasing interest as a means to eradicate therapeutically challenging tumor entities (Viswanathan *et al*, 2017; Friedmann Angeli *et al*, 2019). Early studies reported that ferroptosis is triggered by cyst(e)ine starvation. Cysteine depletion can be initiated pharmacologically by small molecules such as Erastin that can specifically block cystine uptake via inhibiting the amino acid transporter system Xc^−^. System Xc^−^ is a heterodimer consisting of SLC3A2 (also known as 4F2hc or CD98) and the specificity‐conferring subunit SLC7A11 (also known as xCT). Characterization of the molecular events leading to ferroptosis execution has shown that cysteine starvation promotes cell death by the acute depletion of the cysteine‐containing tripeptide glutathione (GSH), which in turn is essential to fuel the activity of the selenoprotein glutathione peroxidase 4 (GPX4; Yang *et al*, 2014). GPX4 utilizes reducing equivalents from glutathione (GSH) to suppress the accumulation of lipid hydroperoxides and, ultimately, the induction of ferroptotic cell death (Shah *et al*, 2018; Fig 1A).

Given that alternative pathways can provide cysteine and thus bypass the requirement for system Xc^−^, such as the transsulfuration pathway, there is an intense search for strategies and molecular tools to trigger ferroptosis at the level of GPX4. GPX4 inhibitors include the covalent inhibitors RSL3 and ML210, which perform in cultured cells but are inefficient in *in vivo* settings (Yang *et al*, 2014; Viswanathan *et al*, 2017; Liu *et al*, 2018; Eaton *et al*, 2019; Fig 1A). Thus, the lack of suitable *in vivo* GPX4 inhibitors and the predicted systemic toxicity of such compounds (Friedmann Angeli *et al*, 2014; Brutsch *et al*, 2015; Carlson *et al*, 2016) limit the translation of these discoveries into cancer therapies. Recently, we, and others, have uncovered that high‐risk *MYCN*‐amplified neuroblastomas are characterized by a striking GPX4 dependency (Floros *et al*, 2021; Lu *et al*, 2021; Alborzinia *et al*, 2022). The molecular determinants of this increased dependency remain elusive and untangling the mechanisms dictating ferroptosis hypersensitive states could potentially pave the way to exploit these vulnerabilities.

In this work, using genome‐wide and single‐cell CRISPR‐activation (CRISPRa) screens, we identify novel regulators of ferroptosis and detect shared transcriptional signatures and states regulating ferroptosis hypersensitivity. Specifically, we identify the low‐density lipoprotein receptor (LDLR)‐related protein 8 (LRP8, also known as APOER2) as a critical suppressor of ferroptosis in *MYCN*‐amplified neuroblastoma. We find that the LRP8 dependency in *MYCN*‐amplified neuroblastomas is partially due to the limited activity of alternative selenium transporters, specifically system Xc^−^. Therefore, our work demonstrates that the utilization of different selenium/selenocysteine acquisition pathways offers unanticipated opportunities to selectively and safely induce ferroptosis for therapeutic benefit.

## Results

### CRISPR activation screens identify novel regulators of ferroptosis

In order to gain additional insights into the process of ferroptosis, we reasoned that understanding the mechanisms that could increase the resistance of intrinsically sensitive cells would provide a means to identify yet uncharacterized regulators of ferroptosis (Fig 1A). Toward this end, we initially analyzed data from the depmap portal (www.depmap.org) in search of cell lines that are hypersensitive to the GPX4 inhibitors RSL3 and ML210 (Fig 1B). We selected the *MYCN*‐amplified neuroblastoma cell line SK‐N‐DZ, a representative of tumor entities that still defy current treatments and for which we and others have already reported a marked dependency on GPX4 (Lewerenz *et al*, 2018; Floros *et al*, 2021; Lu *et al*, 2021; Alborzinia *et al*, 2022), thus providing an ideal setting to interrogate the mechanisms underlying this hypersensitivity. Using this cell line, we performed genome‐wide CRISPR activation (CRISPRa) screens where we induced ferroptosis via two nonoverlapping mechanisms (Fig 1C), namely via inhibition of (i) GPX4 by RSL‐3 or (ii) system Xc^−^ by Erastin (Fig 1A and C). Screen deconvolution based on the enrichment of gRNAs after ferroptosis induction allowed us to identify several known and novel ferroptosis regulators (Fig 1D, Dataset EV1, FDR ≤ 0.05). For instance, we found the overexpression of several known key ferroptosis regulators as hits including GCH1 (Kraft *et al*, 2020), AIFM2 (Bersuker *et al*, 2019; Mao *et al*, 2021), SLC7A11 (Dixon *et al*, 2014), GPX4 (Yang *et al*, 2014), and NQO1 (Sun *et al*, 2016) supporting the validity of our screening results. While the majority of hits were exclusive for one mechanism of ferroptosis induction, we found several overlapping hits (120 shared hits, Appendix Fig S1A). A secondary screen focusing on shared and functionally related hits (list provided in Dataset EV2) confirmed the resistance phenotype for most of the scoring hits when selected against the GPX4 inhibitor RSL3 (Fig 1E). To provide an unbiased understanding of the differential mechanism of cellular states that prevent ferroptosis, we coupled the focused screen to single‐cell RNA‐seq (scRNA‐seq, CROP‐seq) as a readout (Fig 1F). The focused library consisted of two guide RNAs (gRNAs) targeting each of the selected 36 candidate genes together with 10 nontargeting control gRNAs (NT‐ctrl). We obtained high‐quality data from ~11,000 CRISPRa‐assigned cells (mean of ~78,000 reads and median of ~6,600 genes per cell; Appendix Fig S1B). Selecting cells with a clear CRISPRa phenotype (see Materials and Methods) allowed us to retrieve 130 cells on average for each of the selected hits (32/36 target gene identities detected, Appendix Fig S1C). As expected, cells assigned to a particular perturbation cluster showed an increased expression of the targeted gene (Fig 1G, Appendix Fig S1E and F and Dataset EV3). Interestingly, the impact of several CRISPRa phenotypes converged on the expression of known ferroptosis regulators. For instance, overexpression phenotypes of *IRS4* and *MET* shared the upregulation of known ferroptosis suppressors, including the transcription factor *NFE2L2* that drives the expression of genes involved in redox signaling and oxidative protection (Yamamoto *et al*, 2018), the heat‐shock protein *HSPB1*, as well as the CoQ oxidoreductases *AIFM2* (also known as *FSP1* (Bersuker *et al*, 2019; Doll *et al*, 2019); Appendix Fig S1F and G). Gene Ontology analysis of differentially expressed genes upon CRISPRa showed significant enrichment in processes involved in cellular detoxification, energy metabolism, and proteasomal degradation that have been previously linked to ferroptosis (Dixon *et al*, 2012; Gao *et al*, 2019; Chen *et al*, 2021; Appendix Fig S1H). Interestingly, transcriptomic consequences of overexpressing the highest scoring hit in our primary screen, *LRP8*, clustered together with the *GPX4* overexpression phenotype in our scCRISPRa screen, underscoring that the mechanism underlying ferroptosis resistance might overlap and be shared between multiple groups (Fig 1F and Appendix Fig S1D, F and I for the co‐expressed genes between *GPX4* and *LRP8* overexpression). Similarly, regulatory network inference and pathway activity analysis showed coclustering of the *LRP8*‐ and *GPX4*‐OE phenotypes further supporting an underlying shared transcriptional mechanism of ferroptosis resistance (Appendix Fig S1J). In sum, the scCRISPRa secondary screen faithfully recapitulated the gRNA identity in single‐cell assays and simultaneously provided transcriptional signatures of different ferroptosis‐resistant states derived from the genome‐wide screen.

### SELENOP is a critical source of selenium to support the growth of a subset of *MYCN*‐amplified neuroblastomas

**Figure 2:**
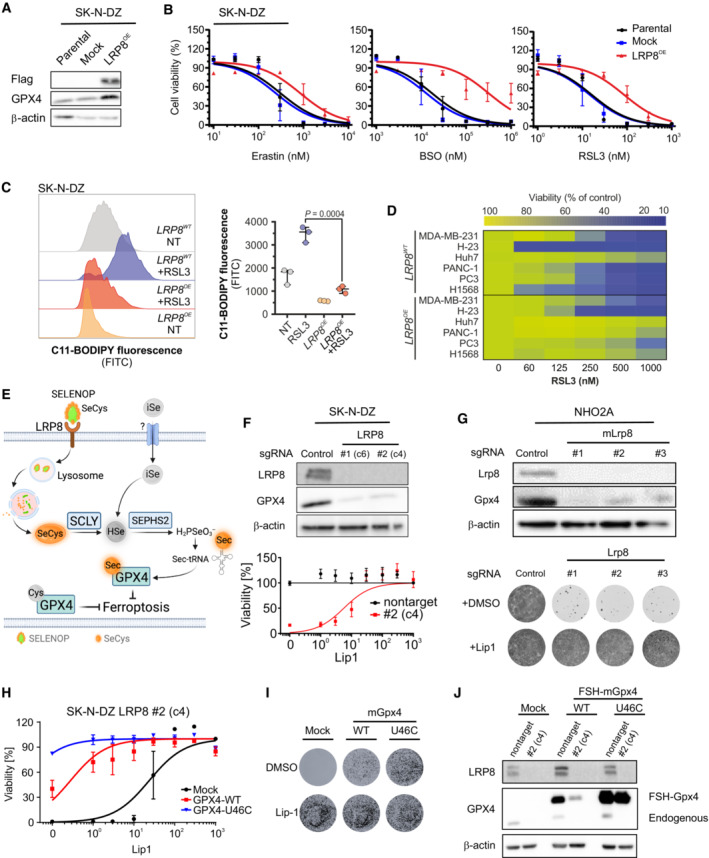
LRP8 loss triggers ferroptosis in *MYCN*‐amplified neuroblastoma Immunoblot analyzis of FLAG and GPX4 in SK‐N‐DZ cells overexpressing an empty vector or *hLRP8*‐Flag.Dose‐dependent toxicity of the ferroptosis inducers Erastin, BSO, and RSL3 in SK‐N‐DZ cell lines stably transduced with a vector expressing *hLRP8*‐Flag. Data are the mean ± SEM of *n* = 3 wells of a 96‐well plate from three independent experiments.Flow cytometry analyzis of BODIPY 581/591 C11 oxidation in SK‐N‐DZ overexpressing *hLRP8*‐Flag induced by RSL3 treatment (100 nM, 6 h) Data represent the mean ± SEM; *n* = 3 samples. Statistical analysis was performed using a two‐tailed Student's *t*‐test.Heat map depicting the dose‐dependent response of RSL3 in a panel of cell lines overexpressing *hLRP8*‐Flag.Schematic representation of selenium uptake mechanisms.Generation and characterization of *LRP8* knockout cell lines using two independent gRNAs. Upper panel, immunoblot analyzis of LRP8 expression in cells transduced with gRNA targeting *LRP8*. Lower panel, the viability of cell lines, either wild‐type or knockout for *LRP8* in the presence of increasing concentrations of Lip‐1. Data are the mean ± SEM of *n* = 3 wells of a 96‐well plate from two independent experiments.Recapitulation of LRP8 dependency in the NHO2A cell line derived from a murine model of *MYCN* amplification. The upper panel depicts the immunoblot of Lrp8 and Gpx4 in cells expressing three independent gRNAs targeting *Lrp8*. The lower panel shows the clonogenic capacity of Lrp8‐deficient cells and the protective effect of Lip‐1 (500 nM).Dose‐dependent response of SK‐N‐DZ *LRP8* knockout clonal cell line (#2) expressing wild‐type and U46C‐GPX4 in the present of Lip1. Data are the mean ± SEM of *n* = 3 wells of a 96‐well plate from two independent experiments.The clonogenic capacity of an SK‐N‐DZ *LRP8* knockout clonal cell line (#2) expressing wild‐type and U46C‐GPX4.Immunoblot analyzis of FLAG and GPX4 in SK‐N‐DZ cells overexpressing flag‐tagged WT or a U46C variant of GPX4 in wild‐type and knockout *LRP8* background. Source data are available online for this figure.

The identification of LRP8 as a critical regulator of ferroptosis sensitivity is consistent with data from the cancer therapeutic response portal (CTRP), showing that *LRP8* expression strongly correlates with resistance to GPX4 inhibitors (ML210, RSL3, and ML162; Appendix Fig S2A; Basu *et al*, 2013). To functionally validate our findings, we first overexpressed *LRP8* using a CRISPRa system in which SK‐N‐DZ cells became robustly resistant to RSL3 treatment (Extended Data Fig 2B). Similarly, stable overexpression of a Flag‐tagged h*LRP8* in SK‐N‐DZ cells conveyed resistance to a panel of ferroptosis inducers covering differential modes of action (Fig 2A and B). In line with a specific inhibitory role for LRP8 in ferroptosis, an overall impact on sensitivity toward other cytotoxic compounds was not observed (Appendix Fig S2C). In accordance with the protective effect observed, *LRP8* overexpression suppressed lipid peroxidation in cells treated with a GPX4 inhibitor (Fig 2C). Furthermore, overexpression of *LRP8* was able to increase ferroptosis resistance in a larger panel of cell lines (Fig 2D). No other members of the LRP family showed similar effects when overexpressed, highlighting the specific role of LRP8 on ferroptosis resistance (Appendix Fig S2D and E).

Next, we explored the mechanism by which LRP8 protects cells from ferroptosis. Previous studies have established that members of the low‐density lipoprotein (LDL) receptor‐related protein, including LRP2 and LRP8, are receptors for the selenium carrier protein SELENOP (Burk *et al*, 2006; Olson *et al*, 2007), indicating that modulation of selenocysteine metabolism would be the likely mechanism by which LRP8 protects cells from ferroptosis (Fig 2E). This premise agrees with the finding that overexpression of *LRP8* leads to upregulation of GPX4 at the protein level (Fig 2A) without impacting RNA levels (Appendix Fig S2F), pointing toward an underlying post‐transcriptional regulation. Next, to address the requirement of LRP8, we generated LRP8‐deficient SK‐N‐DZ cells. Unexpectedly, without additional stressors, we observed that these cells readily underwent massive ferroptosis‐like cell death in the absence of ferroptosis‐inhibiting compounds (Fig 2F and Appendix Fig S2G). To further corroborate this observation and provide mechanical support for targeting LRP8 in neuroblastoma, we deleted *Lrp8* in the murine TH‐MYCN neuroblastoma mouse model (Fig 2G) and a panel of neuroblastoma cell lines with and without *MYCN* amplification (Appendix Fig S2G–I). These additional models recapitulated the observation that LRP8 is essential to support the viability of human neuroblastoma cell lines and that it is also crucial in a cell line derived from a MYCN‐driven genetic neuroblastoma model (Appendix Fig S2H and I).

Furthermore, the loss of viability caused by LRP8 loss could be fully rescued by providing selenite that provides selenium by a mechanism independent of LRP8 and the ferroptosis inhibitor Liproxstatin‐1 (Lip‐1; Appendix Fig S2J). To validate the role of SELENOP, we used two distinct LRP8‐deficient models to show that SELENOP can only boost GPX4 expression in LRP8 proficient cells (Appendix Fig S3A and B). Lastly, the specificity of ferroptosis was confirmed by showing that only canonical inhibitors of ferroptosis can suppress cell death, whereas other cell death inhibitors cannot (Appendix Fig S3C).

Given the important role of selenocysteine metabolism in preventing ferroptosis, we next asked whether any correlation with the clinical outcome can be observed in cohorts of pediatric neuroblastoma patients. While *LRP8* expression was not generally associated with event‐free survival, high expression of *GPX4* and other members of the selenocysteine incorporation machinery (i.e., *SEPHS2*, *PSTK*, *EEFSEC*, and *SECISBP2*) showed a strong correlation with poor disease outcome. This finding is in line with the generally higher expression of members of the selenocysteine biosynthetic pathway in high‐risk neuroblastoma (Appendix Fig S3D). These data suggest that the efficient flux of selenium/selenocysteine through this pathway is a strong determinant for poor survival of neuroblastoma patients and may contribute to therapy resistance. This reasoning agrees with the observation that SK‐N‐DZ cells could not grow in selected batches of fetal bovine serum (FBS), which lack detectable SELENOP unless supplemented with exogenous sources of selenium or ferroptosis‐inhibiting compounds (Appendix Fig S4A and B). Accordingly, the protective effect conferred by the overexpression of *LRP8* was dramatically reduced in low SELENOP conditions (Appendix Fig S4C).

Strong evidence for a direct link between LRP8, selenocysteine, GPX4, and cell survival we provide by showing that overexpression of wild‐type GPX4 was unable to fully rescue cell viability upon loss of *LRP8*. Importantly, the expression of GPX4 (U46C), a mutant that is approximately 100× catalytically less efficient but bypasses the requirement of selenocysteine loaded tRNAs for efficient translation (Ingold *et al*, 2018), could fully restore cell viability (Fig 2H–J). Consequently, only the levels of ectopic wild‐type (WT) and endogenous GPX4 were decreased by LRP8 deficiency, while the ectopic U46C GPX4 level remained unchanged (Fig 2H). In support of this, the knockdown of selenophosphate synthetase 2 (*SEPHS2*), a central enzyme in the metabolization of selenium, significantly reduced ferroptosis resistance of cells overexpressing *LRP8* (Appendix Fig S4D). Additional evidence that ferroptosis is induced in LRP8‐deficient cells was provided by demonstrating that lipid peroxidation can be triggered in the absence of LRP8 and that this is prevented by the addition of ferrostatin‐1 (Fer‐1) or by the overexpression of the GPX4‐independent ferroptosis suppressor *AIFM2* (*FSP1*; Appendix Fig S4E and F; Doll *et al*, 2019). Our data demonstrate that targeting SELENOP uptake via LRP8 is a valuable strategy to efficiently disrupt GPX4 maintenance and trigger ferroptosis in cultured *MYCN*‐amplified neuroblastoma cells.

### Limited activity of system Xc^−^ mediates the dependency on LRP8

**Figure 3:**
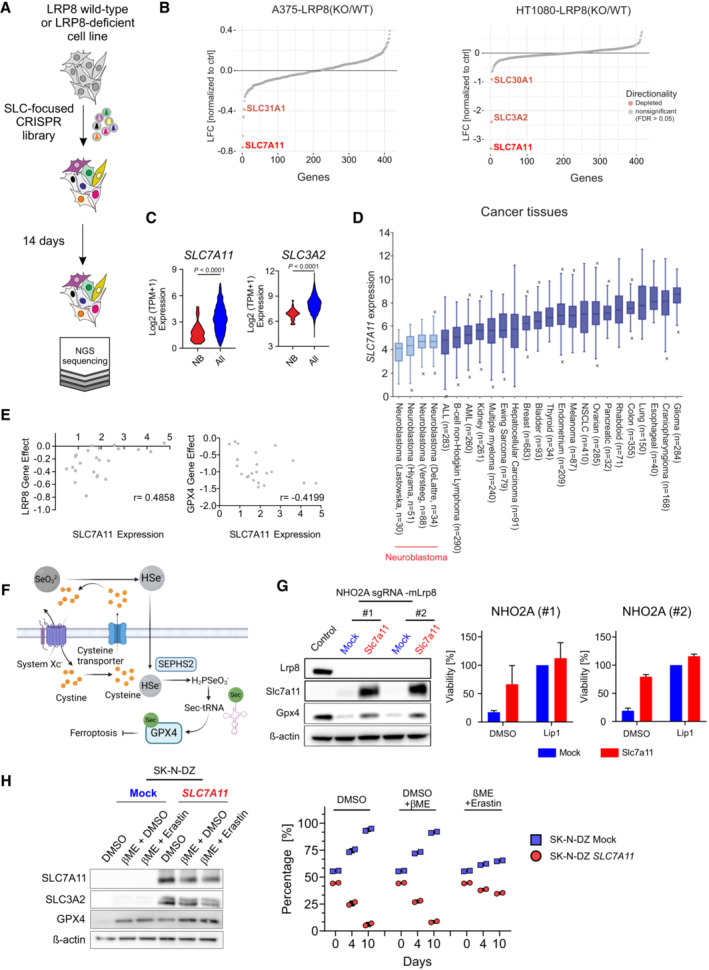
SLC‐focused CRISPR screen identifies alternative selenium uptake mechanisms Schematic representation of the SLC‐focused CRISPR knockout screen in the LRP8 wild‐type and LRP8‐deficient cell lines.Results of a CRISPR deletion screen conducted in H1080 and A375 cell lines displayed a log2 fold change between LRP8‐deficient and wild‐type cells.Comparison of *SLC7A11* expression in a panel of 23 neuroblastoma cell lines against 1,349 non‐neuroblastoma cell lines demonstrating the lineage‐specific lower expression of *SLC7A11* and *SLC3A2* (http://www.depmap.org). Statistical analysis was performed using an Unpaired *t*‐test. ****P < 0.0001.
*SLC7A11* expression in cancer tissues of different entities. Data retrieved from the R2database. The center line in the box plots indicates the median value. Lower and upper hinges represent the 25^th^ and 75^th^ quantiles, the whiskers denote the 1.5× interquartile range, and outliers are marked in the plot.Dot plot depicting the correlation of the dependency of neuroblastoma cell lines on LRP8 and GPX4 (CERES score of −1 means full dependency based on CRISPR–Cas9 knockout screening data) and the expression levels of *SLC7A11* in a panel of 27 neuroblastoma cell lines (depmap portal; https://depmap.org/portal/). Cell lines with low expression of *SLC7A11* were found to be dependent on LRP8 (Pearson correlation *r*: 0.4858).Schematic representation of system Xc^−^ of selenium uptake mechanisms.The immunoblot of Lrp8, Slc7a11 and Gpx4 in NHO2A stably expressing Cas9 and two independent sgRNAs targeting *Lrp8* in an *SLC7A11* or mock expressing background and viability of the cell lines in the presence of the DMSO, Lip1 (500 nM), or a combination of both. Cell viability assays depicted are mean ± SD of two independently performed experiments (*n* = 4 wells of a 96‐well plate).Generation and characterization of cells overexpressing SLC7A11. Immunoblot analysis of SLC7A11 and SLC3A2 from SK‐N‐DZ cells overexpressing SLC7A11 or mock. Cell competition assay of SK‐N‐DZ cell line overexpressing SLC7A11 or mock controls. For the experiment, cells were seeded at a ratio of 50/50 and 50,000 events were measured via flow cytometry at the depicted time points. Rescue experiments were performed in the presence of 50 μM beta‐Mercaptoethanol (beta‐ME) and 2 μM Erastin. Bars display percentage of eGFP‐ (blue) and Scarlet‐cells (red) with the means ± SD of two independent experiments. Statistical analysis was performed using an Unpaired *t*‐test comparing the abundance of SK‐N‐DZ cells overexpressing SLC7A11 (Red). Source data are available online for this figure.

Our observation that *MYCN*‐amplified neuroblastoma cells are highly dependent on LRP8 for selenium uptake is unanticipated, given that alternative forms and uptake mechanisms exist (Burk & Hill, 2015). Moreover, the specification analysis of FBS showed that selenite and selenate were present at approximately 1–2 nM. Still, these forms of selenium could not sufficiently support selenocysteine biosynthesis and consequently could not prevent ferroptosis in neuroblastoma cell lines (Appendix Fig S4B). Our rescue experiments suggested that additional 20‐fold excess of selenite was required to rescue viability (Appendix Fig S4B). These findings led us to hypothesize that alternative mechanisms of selenium provision must be inefficient or inactive in neuroblastoma cells. In order to gain insights into these alternative mechanisms, we performed a series of CRISPR‐based screens focusing on solute carriers (SLCs), as these transporters are the major mediators of soluble metabolite uptake into cells (Superti‐Furga *et al*, 2020). Aiming to identify SLC synthetic lethal interactions with LRP8 loss, we used cells in which the deletion of *LRP8* did not lead to the loss of viability (HT1080 and A375; Appendix Fig S5A–D). Despite being viable upon genetic loss of *LRP8*, these cell lines display a high sensitivity toward several ferroptosis inducers (Appendix Fig S5B and D). Interestingly, screens in both cell lines retrieved the system Xc^−^ subunits SLC7A11 and SLC3A2 as genes generating synthetic lethality with LRP8 deficiency (Fig 3A and B).

This indicates that system Xc^−^, in addition to its previously reported role in indirect uptake (Olm *et al*, 2009; Carlisle *et al*, 2020), is a major contributing factor for the direct uptake of inorganic (SeO_3_^2−^) and organic forms (SeCys) of selenium (Fig 3B). In line with these findings, we noted that neuroblastoma show a generally low expression level of both system Xc^−^ subunits (*SLC7A11* and *SLC3A2*) compared with cancer tissues and cell lines of other entities (Fig 3C and D, and Appendix Fig S4H). These data suggest that the sensitivity and therefore dependency of neuroblastoma to *LRP8* deletion could be at least partly due to the reduced activity of alternative selenium transporters. Publicly available data (depmap portal) indicate that in neuroblastomas, LRP8 dependency is inversely correlated with *SLC7A11* expression with *LRP8* knockout sensitive cell lines exhibiting lower *SLC7A11* expression levels, while this is not observed for GPX4 (Fig 3E and Appendix Fig S4G). Lastly, providing support for our hypothesis that low *SLC7A11* contributes to the increased LRP8 dependency, our data show that overexpression of *SLC7A11* was able to restore GPX4 levels and partially rescue the viability of LRP8‐deficient cells (Fig 3F and G). However, despite these observations, we noted that *SLC7A11* overexpression resulted in a profound growth defect of neuroblastoma cells. This effect was specific to SLC7A11 activity, as it was rescued by the addition of the system Xc^−^ inhibitor Erastin (Fig 3H). Our initial characterization further demonstrated that in an isogenic model where MYCN is inducibly activated, the process was both aggravated by MYCN activity and cystine lelels (Appendix Fig S6A–D). Altogether, our data suggest that low expression of system Xc^−^ components that limit intracellular uptake of inorganic selenium is the likely cause for the remarkable dependence of neuroblastoma cells on LRP8.

### LRP8 is required for the initiation and maintenance of neuroblastoma *in vivo*

**Figure 4:**
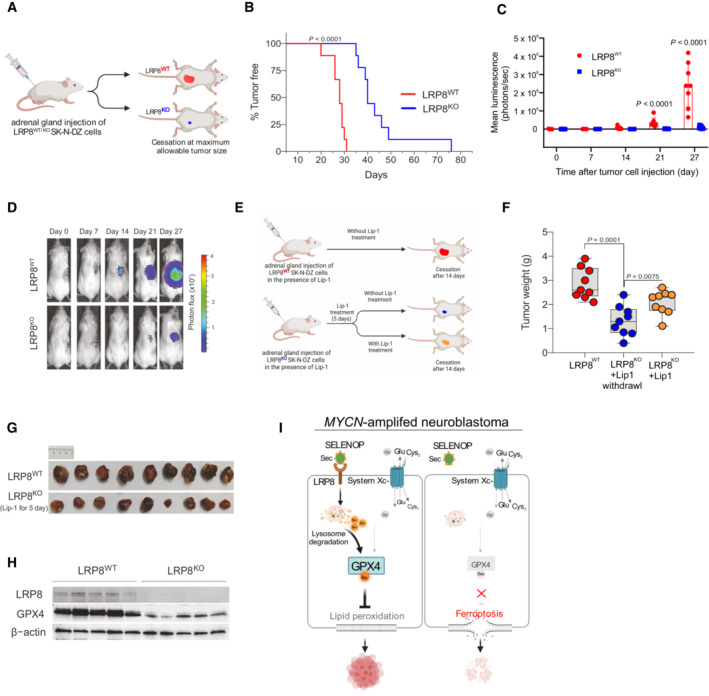
LRP8 is essential for orthotopic neuroblastoma growth Schematic representation of the orthotopic implantation of control (LRP8^WT^) or LRP8‐deficient (LRP8^KO^) SK‐N‐DZ cell lines.Kaplan–Meier plot displaying tumor‐free survival (TFS) for mice injected orthotopically with LRP8^WT^ (red, *n* = 9) or LRP8^KO^ (blue, *n* = 9) SK‐N‐DZ cells. A Gehan–Breslow–Wilcoxon test was conducted for statistical analyzis.Tumor growth upon orthotopic implantation of LRP8^WT^ (red, *n* = 9) or LRP8^KO^ (blue, *n* = 9) of SK‐N‐DZ cell line. Data represent the mean ± SEM; Mann–Whitney test one‐tailed *P*‐values are indicated.Representative luminescence images from each group are sown in (C).Outline of the orthotopic implantation and treatment scheme with Liproxstatin‐1 (Lip‐1) of LRP8^WT^ and LRP8^KO^ SK‐N‐DZ cells.Tumor weight of orthotopically implanted LRP8^WT^ (red, *n* = 9) or LRP8^KO^ (blue and yellow, *n* = 9 each) SK‐N‐DZ cells. All mice were treated with Lip‐1 for 5 days. After this, treatment was ceased for LRP8^WT^ (red) and LRP8^KO^ cohorts (blue) or maintained for an additional 14 days (yellow); see Materials and Methods for details. Tumors were analyzed at the endpoint. The center line in the box plots indicates the median value. Lower and upper hinges represent the 25^th^ and 75^th^ quantiles, the whiskers denote the Max and Min value. Mann–Whitney test one‐tailed *P* values are indicated.Representative images of tumors in the control (red) and LRP8^KO^ groups (blue) treated with Lip‐1 for 5 days after implantation.Immunoblot analyzis of LRP8 and GPX4 levels from orthotopic tumors of LRP8^WT^ or LRP8^KO^ SK‐N‐DZ cells, treated with Lip‐1 for 5 days after implantation, followed by 14 days after randomization.Schematic representation for the proposed model of LRP8 inhibition essentiality. Comparison of selenium /selenocysteine uptake mechanisms via LRP8 and system Xc^−^ in proliferating MYCN‐amplified cells, depicting the contribution of primarily LRP8/SELENOP supporting selenoprotein translation (left panel). Inhibition of LRP8 in system Xc^−^ low cell selectively triggers ferroptosis in *MYCN*‐amplified neuroblastoma (right panel). SELENOP, Selenoprotein P; iSe, inorganic selenium; SeCys, Selenocysteine. Source data are available online for this figure.

Given the critical role played by LRP8 and the selenocysteine metabolism in preventing ferroptosis in highly aggressive neuroblastoma subtypes with *MYCN* amplifications, we addressed whether LRP8 elimination could target neuroblastoma growth by inducing ferroptosis *in vivo*. We took advantage of our reported orthotopic animal model in which SK‐N‐DZ cells were implanted in the adrenal gland of NOD.Cg‐Prkdc^scid^Il2rgtm1^Wjl^/SzJ (NSG) mice (Alborzinia *et al*, 2022). Briefly, all cells were grown in the presence of liproxstatin‐1 (Lip‐1) before implantation to prevent ferroptotic cell death of LRP8‐deficient neuroblastoma cells. Subsequently, SK‐N‐DZ cells were implanted, and tumor growth was monitored using *in vivo* bioluminescence imaging (Fig 4A). LRP8‐deficient tumors showed a marked decrease in tumor growth and, compared with LRP8 proficient cells, showed a significantly increased overall survival of mice (76d ± vs. 31d ±, defined by reaching termination criteria) with a median survival of 29 days vs. 40 days (Fig 4B–D). More clinically relevant, we explored the therapeutic potential of targeting LRP8 in established SK‐N‐DZ neuroblastoma. For this, LRP8 proficient (LRP8^WT^) and deficient (LRP8^KO^) tumors were both allowed to grow in the presence of *in vivo* active Lip‐1 (Fig 4E), with the former ones serving as controls (Fig 4E, top, red). After LRP8‐deficient neuroblastomas were established (seven days of postimplantation with five days of Lip‐1 treatment), mice were randomized into two groups. Lip‐1 treatment was continued in the yellow group, while in the blue group, Lip‐1 injections, and thus ferroptosis inhibition, were stopped (Fig 4E, bottom). Analysis of the three cohorts after 14 days of postimplantation showed that both LRP8‐deficient xenograft groups (blue, yellow) showed impaired tumor growth compared with WT controls (red) despite Lip‐1‐mediated ferroptosis inhibition (Fig 4F and G). Importantly, Lip‐1 withdrawal in the randomized LRP8^KO^ group for only 9 days (the maximum time approved by the animal protocol) already significantly reduced neuroblastoma growth also in established tumors, mimicking the clinical situation (Fig 4F and G). The data above support the requirement of LRP8 for the establishment and maintenance of *MYCN*‐amplified SK‐N‐DZ neuroblastoma by preventing ferroptosis. This is further supported by the robust loss of GPX4 protein in LRP8^KO^ tumors (Fig 4H). Collectively, our data indicate that LRP8 is required to prevent ferroptosis by maintaining high levels of GPX4 in *MYCN*‐amplified orthotopic neuroblastoma models and suggest that inhibition of the SELENOP/LRP8 axis as a novel and selective strategy to trigger ferroptosis and thereby limit tumor growth in highly aggressive and hard to treat MYCN‐amplified neuroblastoma cells (Fig 4I).

## Discussion

The present study demonstrates that blocking selenium/selenocysteine uptake mechanisms could be an attractive indirect strategy to disrupt GPX4 function specifically and selectively to induce ferroptotic cell death in *MYCN*‐amplified neuroblastoma. We show here that selenium/selenocysteine can be obtained by cancer cells via multiple routes: First by the SELENOP/LRP8 axis and second, via the activity of system Xc^−^ (SLC7A11/SLC3A2). Our data also imply that the striking LRP8 dependency may have emerged during tumor evolution as a result of the growth advantage of *MYCN*‐amplified neuroblastoma displaying low expression/activity of system Xc^−^. Of note, our study shows that forced expression of SLC7A11 prevents cell death of LRP8‐deficient neuroblastoma, but it also demonstrates its negative impact on the survival of these cells. While the mechanism is currently not clear, a possibility is that cystine might evoke dissulfide stress in a manner similar to a process dubbed disulfidptosis and described in a recent publication (Liu *et al*, 2023).

Thus, LRP8 blockade represents a rational strategy to indirectly deplete GPX4 and selectively trigger ferroptosis in *MYCN*‐amplified neuroblastoma and potentially other entities with low system Xc^−^ expression/activity such as AML and lymphoma, while sparring normal cells. Of notice, recent reports have also identified LRP8 as a strong predictor of ferroptosis resistance both in cancer and in neurons (Greenough *et al*, 2022; Li *et al*, 2022). In both studies, the loss of LRP8 was not sufficient to spontaneously trigger ferroptosis neither *in vitro* nor *in vivo*. This echoes our observation that these dependencies are specific to defined contexts related to the fueling of the selenocysteine pathways. Overall, these discoveries could have broader implications. A recently published cancer dependency map of pediatric tumors identified *LRP8* as an essential gene in pediatric Ewing sarcoma and medulloblastoma, all entities associated with *MYCN* amplifications (Dharia *et al*, 2021). Moreover, given the largely unsuccessful repurposing of adult oncology drugs for treating neuroblastoma and other childhood malignancies, these discoveries could provide an attractive framework to target ferroptosis in this entity. Targeting LRP8, for example, by developing LRP8/SELENOP neutralizing antibodies in *MYCN*‐amplified settings may overcome potential limitations of strategies that aim to inhibit GPX4 directly, expected to cause widespread organ toxicity as indicated by mouse genetic studies (Friedmann Angeli & Conrad, 2018).

One aspect that deserves attention is that all our studies are carried out in immunocompromised settings, and given that recent reports have suggested that CD8^+^ T‐Cells can drive tumor regression partially via ferroptosis (Matsushita *et al*, 2015; Liao *et al*, 2022) the effects observed here might be to some extent underestimated. Nevertheless, direct targeting of GPX4 in an immunocompetent setting might be a double‐edged sword as it could impair T‐cell function (Matsushita *et al*, 2015). Yet, targeting LRP8 might bypass such an unwanted off‐target effect since CD8^+^ T‐cells lack *LRP8* expression. Therefore, additional studies on the inhibition of LRP8 in immunocompetent models are warranted to fully understand the therapeutic potential of targeting the LRP8/SELENOP axis for therapeutic benefit in neuroblastoma and other malignancies in particular the ones with amplified MYCN.

## Materials ans Methods

### Cell culture

The human neuroblastoma cell line SK‐N‐DZ (purchased from ATCC) was maintained in DMEM medium (Gibco) supplemented with 10% FCS (Gibco), 1× HEPES (Gibco), and 100 U/ml penicillin/streptomycin (Gibco) at 37°C with 5% CO_2_. Cell lines were tested for identity by SNP genotyping and mycoplasma contamination by the respective Multiplexion service (Heidelberg, Germany).

The human neuroblastoma cell line SH‐EP MYCN‐ER (Kindly provided by Prof. Dr. Martin Eilers; Herold *et al*, 2019), the murine neuroblastoma cell line NHO2A (derived from TH‐MYCN murine neuroblastoma model, kindly provided by Prof. Per Dr. Kogner), the human melanoma cell line A375 (purchased from ATCC), the human fibrosarcoma cell line HT1080 (purchased from ATCC) and the human hepatocellular carcinoma HepG2 (purchased from ATCC) were maintained in DMEM medium (Gibco) supplemented with 10% FCS (Gibco) and 100 U/ml penicillin/streptomycin (Gibco) at 37°C with 5% CO_2_. Cell lines were tested for mycoplasma contamination by the respective Multiplexion service (Heidelberg, Germany).

### Immuoblotting

Cells were lysed in RIPA Buffer containing protease (Roche) inhibitor cocktail. Proteins were separated by SDS–polyacrylamide gel electrophoresis and electro transferred onto polyvinylidene difluoride membranes that were subsequently blocked in 5% defatted milk in Tris‐buffered saline (TBS) for 1 h room temperature (RT). Primary antibodies were diluted in a solution of 5% bovine serum albumin (BSA) in TBS and incubated overnight at 4°C. Membranes were washed and then incubated at RT for 2 h with horseradish peroxidase‐labeled secondary (1:3,000, anti‐rabbit #7074, anti‐mouse #7076 or anti‐rat #7077, Cell Signaling) diluted in a solution of 5% milk in TBS. Proteins were detected using enhanced chemiluminescence substrate (107‐5061, BioRad). Images were acquired using detection systems Amersham ImageQuant 800 (Cytiva, EUA) or Azure 400, (Biozym, Germany).

Following antibodies were used against β‐actin (1:5,000; A5441, Sigma‐Aldrich), GPX4 (1:1,000; no. ab125066, abcam), LRP8 (1:1,000; ab108208, abcam), SLC3A2 (CD98, 1:1,000; sc‐376815, Santa Cruz), SLC7A11 (1:10; antibody raised against a N‐terminal peptide of SLC7A11, clone 15C2‐111, developed in Munich + NaN_3_), SCLY (1:50; IG‐P1097, ImmunoGlobe GmbH), Flag (1:3,000; F3165, Sigma‐Aldrich), GPX1 (1:1,000; #3286, Cell Signaling), mouse Slc7a11 (1:10; monoclonal antibody generated at the HMGU, clone 4B3‐1113, developed in Munich + NaN_3_).

### Molecular cloning

The human CRISPR activation pooled library Set A (Addgene plasmid #92379 was a gift from David Root and John Doench) was amplified as described previously (Joung *et al*, 2017). Briefly, Endura electrocompetent cells (Lucigen, Cat. No. 60242) were used to perform six electroporation reactions on a MicroPulserTM II (BioRad) following the manufacturer's instructions (pre‐set EC1 setting with V: 1.8 kV). Subsequently, cells were pooled and recovered at 37°C for 45 min on a shaking incubator. The cell suspension was diluted with LB‐medium, and 2 ml of bacterial suspension was spread evenly on prewarmed LB‐agar plates containing carbenicillin (100 μg/ml, 1× 245 mm square dish per 10,000 gRNAs in library) and incubated for 12 h at 37°C. The electroporation efficiency was assessed, and cells were harvested when library representation was > 100 bacterial colonies per gRNA. According to the manufacturer's instructions, the plasmid DNA was purified using NucleoBond® Xtra Maxi EF (Macherey‐Nagel, Cat. No. 740424). Sequencing libraries were prepared using the NEBNext 2× High‐Fidelity Master Mix (NEB, Cat. No. M0541L) with amplification primers partially complementary to the lentiviral gRNA backbone with overhangs introducing the Illumina P5/P7 adapter sequences. A pool of staggered P5 primer and 5% PhiX spike‐in were used to increase sequence diversity. Maintenance of gRNA distribution and library complexity was confirmed via next‐generation sequencing (NGS) on a Hi‐Seq2000 with the following read configuration: 125 cycles Read 1, 8 cycles index 1 (i7). Sequencing was performed by the High‐Throughput Sequencing Unit of the DKFZ Genomics and Proteomics Core Facility.

For the validation and the scCRISPRa screens, a custom gRNA library was constructed with selected hits from the primary screen. A pool of 82 oligonucleotides containing two gRNAs for each target and 10 NT control gRNAs were ordered from Twist Bioscience (San Francisco, USA) and cloned into a modified CROP‐seq‐MS2 vector via Golden Gate Assembly (NEB, Cat. No. E5520). The modified CROP‐seq‐MS2 plasmid was obtained as follows: the lentiviral CROP‐seq‐opti vector (Addgene plasmid #106280 was a gift from Jay Shendure) was sub‐cloned via restriction digest of the plasmid with NsiI‐HF (NEB, Cat. No. R3127) and SnaBI (NEB, Cat. No. R0130) and the insertion of a synthetic dsDNA fragment coding for a gRNA scaffold sequence with MS2 stem‐loop motifs (manufactured by Synbio Technologies, South Brunswick Township, USA; Sequence: 5′‐ATGCATGCTCTTCAACCTCAATAACGTTATATCCTGATTCACGTAATATTTTTGGGGTAAATTTAGTTCCTGTTCCATTAACTGCGCTAAAAATAATTTTTAAATCTTTTTTAGCTTCTTGCTCTTTTTTGTACGTCTCTGTTTTAGAGCTAGGCCAACATGAGGATCACCCATGTCTGCAGGGCCTAGCAAGTTAAAATAAGGCTAGTCCGTTATCAACTTGGCCAACATGAGGATCACCCATGTCTGCAGGGCCAAGTGGCACCGAGTCGGTGCTTTTTTTAAGCTTGGCGTAACTAGATCTTGAGACACTGCTTTTTGCTTGTACTGGGTCTCTCTGGTTAGACCAGATCTGAGCCTGGGAGCTCTCTGGCTAACTAGGGAACCCACTGCTTAAGCCTCAATAAAGCTTGCCTTGAGTGCTTCAAGTAGTGTGTGCCCGTCTGTTGTGTGACTCTGGTAACTAGAGATCCCTCAGACCCTTTTAGTCAGTGTGGAAAATCTCTAGCAGTACGTA‐3′). The MS2 loops in the gRNA scaffold allow the recruitment of the p65‐HSF1 transactivator complex (expressed from pLentiMPH2). Next, the puromycin resistance gene was extended with a sequence coding for a viral p2A self‐cleaving motif and a tagBFP, thereby removing the stop codon from the puromycin resistance gene. For this, the plasmid was linearized with BstEII‐HF (NEB, Cat. No. R3162) and MluI‐HF (NEB, Cat. No. R3198), and the synthetic dsDNA fragment (manufactured by Twist Bioscience, Sequence: 5′‐GGTCACCGAGCTGCAAGAACTCTTCCTCACGCGCGTCGGGCTCGACATCGGCAAGGTGTGGGTCGCGGACGACGGCGCCGCCGTGGCGGTCTGGACCACGCCGGAGAGCGTCGAAGCGGGGGCGGTGTTCGCCGAGATCGGCCCGCGCATGGCCGAGTTGAGCGGTTCCCGGCTGGCCGCGCAGCAACAGATGGAAGGCCTCCTGGCGCCGCACCGGCCCAAGGAGCCCGCGTGGTTCCTGGCCACCGTCGGAGTCTCGCCCGACCACCAGGGCAAGGGTCTGGGCAGCGCCGTCGTGCTCCCCGGAGTGGAGGCGGCCGAGCGCGCCGGGGTGCCCGCCTTCCTGGAGACCTCCGCGCCCCGCAACCTCCCCTTCTACGAGCGGCTCGGCTTCACCGTCACCGCCGACGTCGAGGTGCCCGAAGGACCGCGCACCTGGTGCATGACCCGCAAGCCCGGTGCCGGAAGCGGAGCTACTAACTTCAGCCTGCTGAAGCAGGCTGGCGACGTGGAGGAGAACCCTGGACCTGGAGGGTCAGGGGGAGCCACCATGGTGTCTAAGGGCGAAGAGCTGATTAAGGAGAACATGCACATGAAGCTGTACATGGAGGGCACCGTGGACAACCATCACTTCAAGTGCACATCCGAGGGCGAAGGCAAGCCCTACGAGGGCACCCAGACCATGAGAATCAAGGTGGTCGAGGGCGGCCCTCTCCCCTTCGCCTTCGACATCCTGGCTACTAGCTTCCTCTACGGCAGCAAGACCTTCATCAACCACACCCAGGGCATCCCCGACTTCTTCAAGCAGTCCTTCCCTGAGGGCTTCACATGGGAGAGAGTCACCACATACGAAGACGGGGGCGTGCTGACCGCTACCCAGGACACCAGCCTCCAGGACGGCTGCCTCATCTACAACGTCAAGATCAGAGGGGTGAACTTCACATCCAACGGCCCTGTGATGCAGAAGAAAACACTCGGCTGGGAGGCCTTCACCGAAACTCTGTACCCCGCTGACGGCGGCCTGGAAGGCAGAAACGACATGGCCCTGAAGCTCGTGGGCGGGAGCCATCTGATCGCAAACGCCAAGACCACATATAGATCCAAGAAACCCGCTAAGAACCTCAAGATGCCTGGCGTCTACTATGTGGACTACAGACTGGAAAGAATCAAGGAGGCCAACAACGAGACCTACGTCGAGCAGCACGAGGTGGCAGTGGCCAGATACTGCGACCTCCCTAGCAAACTGGGGCACAAGCTTAATTAAACGCGT‐3′) was inserted to the plasmid backbone thus generating CROP‐seq‐MS2. The custom gRNA library was amplified in electrocompetent Endura bacteria (Lucigen, Cat. No. 60242), and plasmid DNA was purified as described. Library complexity was verified via NGS using the MiSeq V3 kit (Read configuration: 167 cycles Read 1, 8 cycles index 1 [i7]).

Individual gRNAs were cloned into the modified CROP‐seq‐MS2 or the pXPR_502 (Addgene #96923 was a gift from John Doench and David Root) lentivectors for CRISPRa experiments, and the pLKO5_RFP657 backbone for knockout (CRISPR‐KO) experiments via restriction digest of the respective lentivector with BsmBI (NEB, Cat. No. R0739). Oligonucleotides (Sigma‐Aldrich) with the gRNA sequences and complementary overhangs were phosphorylated, annealed, and inserted into the respective lentiviral delivery vector (Table 1).

**Table 1:** Oligonucleotides used for cloning of individual gRNAs.

Name	Sequence (5′‐3′)
Sense LRP8‐KO #1	CACCGGCCACTGCATCCACGAACGG
Antisense LRP8‐KO #1	AAACCCGTTCGTGGATGCAGTGGCC
Sense LRP8‐KO #2	CACCGCTGCTTAGACCACAGCGACG
Antisense LRP8‐KO #2	AAACCGTCGCTGTGGTCTAAGCAGC
Sense LRP8‐OE #1	CACCGGGGCGGAGGCGGCAGCGGGA
Antisense LRP8‐OE #1	AAACTCCCGCTGCCGCCTCCGCCCC
Sense LRP8‐OE #2	CACCGGGCAGAGCCGAGTCAGAGAC
Antisense LRP8‐OE #2	AAACGTCTCTGACTCGGCTCTGCCC
Sense AIFM2‐OE #1	CACCGTAACCTTGACCCTGAGCGAA
Antisense AIFM2‐OE #1	AAACTTCGCTCAGGGTCAAGGTTAC
Sense LRP8‐VBC #2	CACCGTACGGCTGAAGAGAGTGCGT
Antisense LRP8‐VBC #2	AAACACGCACTCTCTTCAGCCGTAC
Sense LRP8‐VBC #3	CACCGGCAATAAGACCATCTCAG
Antisense LRP8‐VBC #3	AAACCTGAGATGGTCTTATTGCC
Sense LRP8‐exon9 #3	CACCGTACCATTACCTAGCCATGGA
Antisense LRP8‐exon9 #3	AAACTCCATGGCTAGGTAATGGTAC
Sense LRP8‐exon9 #4	CACCGGAACCTGGGGACTTAGACC
Antisense LRP8‐exon9 #4	AAACGGTCTAAGTCCCCAGGTTCC
Sense mLrp8 #1	CACCGATCTTCACGAACCGACACG
Antisense mLrp8 #1	AAACCGTGTCGGTTCGTGAAGATC
Sense mLrp8 #2	CACCGAGGTGCGGAGGATAGACC
Antisense mLrp8 #2	AAACGGTCTATCCTCCGCACCTC

### Lentivirus production

Large‐scale lentivirus production was performed using a second‐generation lentivirus system and a calcium phosphate transfection kit (Invitrogen, Cat. No. K278001) in HEK293T cells. Briefly, early passaged HEK293T cells were cotransfected with the lentiviral transfer plasmid, a packaging plasmid (psPAX2, Addgene plasmid #12260 was a gift from Didier Trono), as well as with a plasmid encoding the VSV‐G envelope (pMD2.G, Addgene plasmid #12259 was a gift from Didier Trono). Viral supernatant was collected 48‐h post‐transfection, snap frozen, and stored at −80°C until use. Alternatively, for the generation of the cell expressing the following constructs; p442‐LRP8‐Flag, p442‐Mock, p442‐hSLC7A11, p442‐mSlc7a11, p442‐GFP and p442‐mScarlet, HEK293T cells were used to produce replication‐incompetent lentiviral particles pseudotyped with the ecotropic envelope protein of the murine leukemia virus (MLV) or the pantropic envelope protein VSV‐G. The third‐generation packaging plasmids (MDLg_pRRE and pRSV_Rev) and transfer plasmids were co‐transfected into HEK293T cells. Cell cultural supernatant containing viral particles was harvested 48‐h post‐transfection and used to transduce cell lines of interest. All experimental procedures for lentivirus production and transduction were performed in a biosafety level 2 laboratory.

### Cell line generation for CRISPRa and CRISPR‐KO experiments

Polyclonal SK‐N‐DZ cells constitutively expressing the CRISPR activation machinery were engineered by transducing wild‐type cells with lentiviral particles carrying a dCas9‐VP64 (lenti dCas9VP64_Blast, Addgene plasmid #61425 was a gift from Feng Zhang) at a multiplicity of infection (MOI) of ~0.5. After recovery, cells were selected via Blasticidin treatment (20 μg/ml).

For the validation and scCRISPRa screens, SK‐N‐DZ cells expressing dCas9‐VP64 were transduced with lentiviral particles encoding the p65‐HSF1 transactivator complex (pLentiMPH2, Addgene plasmid #89308 was a gift from Feng Zhang). After recovery and selection with hygromycin B (200 μg/ml, Gibco, Cat. No. 10687010), cells were individualized by fluorescence‐activated cell sorting based on physical parameters (forward scatter and side scatter) and exclusion of dead cells via DAPI staining. Clonal cell lines were established and tested for their CRISPRa potential. Two independent clonal SK‐N‐DZ cell lines were selected for the validation screen, while the best‐scoring clonal cell line was used for the scCRISPRa screen.

For the CRISPR‐KO experiments, cells were transduced with lentivirus carrying a transgene that encodes a doxycycline‐inducible wild‐type Cas9 nuclease (pCW‐Cas9‐EGFP). After recovery, Cas9‐inducible cells were transduced with the gRNA lentivirus (pLKO5‐RFP657 backbone) at an MOI of ~0.3. Double‐positive cells (EGFP/RFP657) were individualized via FACS with the exclusion of dead cells (DAPI negative). Cas9 expression was induced by adding doxycycline (1 μg/ml, Sigma‐Aldrich, Cat. No. D9891) into the cell culture medium.

### Genome‐wide CRISPRa screen to identify negative regulators of ferroptosis

Polyclonal SK‐N‐DZ cells expressing dCas9‐VP64 were transduced with the genome‐wide Calabrese CRISPRa library in triplicate at an MOI of ~0.3. For each replicate, 190 million cells were transduced, achieving a representation of ~1,000 cells per gRNA. After initial recovery for 4 days, cells were selected with puromycin (0.65 μg/ml) for 7 days. For each replicate, cells were split into four groups, of which one was harvested to determine baseline gRNA representation, another one maintained as the control group, and two others were treated with one of the ferroptosis‐inducing agents, RSL3 or Erastin, respectively, at a concentration lethal for wild‐type SK‐N‐DZ (300 nM/1 μM). Cells were maintained either with the addition of puromycin (control group) or puromycin and the respective drug for additional 2 weeks. The control cells were passaged, maintaining the gRNA representation of 1,000 cells per gRNA throughout the screen. Cells were harvested once the treatment groups reached approximately the same number as the control groups.

The genomic DNA was extracted using a commercial kit (Quick‐DNA Midirep Plus kit, Zymo Research, Cat. No. D4075) following the manufacturer's instructions and NGS libraries prepared as described. Libraries were sequenced as a multiplexed pool on a single lane of a HiSeq2000 chip with the following read configuration (125 cycles Read 1, 8 cycles index 1 [i7]).

### Genome‐wide CRISPRa screen analysis and selection of candidate hits

Raw sequencing reads were processed as previously described (Spahn *et al*, 2017). Briefly, reads were trimmed to remove the constant vector sequence upstream of the gRNA sequences with *cutadapt* and then mapped to the reference library with *Bowtie2*. For each gRNA, the assigned reads were counted and normalized (cpm, counts per million reads). Using the control samples, a negative binomial count distribution was estimated and used to determine fold changes of individual gRNAs in the treatment samples. *P*‐values were computed and corrected for multiple testing (FDR). Finally, a gene‐level score was calculated from the mean log fold‐change of all gRNAs targeting the same gene. Candidate hits were selected using the top 100 enriched genes and examining protein interactions via StringDB (Szklarczyk *et al*, 2019) network analysis (R package version 2.4.0). Those hits were selected that showed a predicted interaction in the network (total of 36 genes, 162 predicted interactions, *P*‐value: 0.0055).

### Secondary validation CRISPRa screen

Two monoclonal SK‐N‐DZ cell lines constitutively expressing dCas9‐VP64 and the p65‐HSF1 transactivator complex were transduced with the custom gRNA library targeting the transcription start sites of 36 selected hits from the primary screen (gRNA representation > 2,000×). After recovery and selection with puromycin (1 μg/ml), cells were split into one control group and a treatment group with RSL‐3 (100 nM). Cells were harvested after 2 weeks, and sequencing libraries were prepared as described. Libraries were sequenced as a multiplexed pool on a MiSeq V3 (Read 1: 167 cycles, index 1 [i7]: 8 cycles).

### Secondary validation screen analysis

A count matrix of gRNA abundances was determined as described for the primary screen while mapping the reads to the custom reference library. To compute enrichment scores and *P*‐values, the robust rank aggregation workflow in the MaGeCK (Li *et al*, 2014) pipeline was used.

### Single‐cell CRISPRa screen

A monoclonal SK‐N‐DZ expressing dCas9‐VP64 and p65‐HSF1 was transduced in duplicate with the custom gRNA library in the CROP‐seq‐MS2 backbone at a low MOI of ~0.07 to minimize the integration of multiple gRNAs. Transduction efficiency was confirmed via flow cytometric analysis of tagBFP expression. Cells were selected with puromycin at 1 μg/ml 24 h post‐transduction. Puromycin selection was continued for additional 4 days at 1.5 μg/ml. Ten days after transduction, cells were collected, and ~20,000 individual cells per lane were partitioned by the 10× Chromium Controller. Single‐cell RNA‐seq libraries were constructed using the Chromium Single Cell Gene Expression v3.1 kit (10× Genomics, Cat. No. 1000128) following the manufacturer's protocol, thereby retaining 40 ng of full‐length cDNA for the enrichment of gRNAs. Guide RNA sequences were selectively amplified by adapting a hemi‐nested PCR approach that was previously described (Hill *et al*, 2018) using the gRNA_enrichment1_fw (5′‐GTGACTGGAGTTCAGACGTGTGCTCTTCCGATCTCTTGTGGAAAGGACGAAACACCG‐3′) and gRNA_enrichment1_rv (5′‐CTACACGACGCTCTTCCGATCT‐3′) primers and the 2× KAPA HiFi HotStart Ready Mix (Roche, Cat. No. KK2601). In a subsequent PCR, 1 ng of purified PCR product was used as input to construct sequencing‐ready libraries with the gRNA‐enrichment2_fw (5′‐CAAGCAGAAGACGGCATACGAGAT[index1]GTGACTGGAGTTCAG‐3′) and gRNA_enrichment2_rv (5′‐AATGATACGGCGACCACCGAGATCTACACTCTTTCCCTACACGACGCTC‐3′) primers. The whole‐transcriptome single‐cell libraries were sequenced on a NovaSeq6000 with 28 cycles Read 1, 8 cycles index 1 (i7), and 91 cycles Read 2. The gRNA enrichment libraries were sequenced on a NextSeq550 high with the following read configuration: 32 cycles Read 1, 8 cycles index 1 (i7), and 120 cycles Read 2.

### Single‐cell CRISPRa screen analysis

Whole‐transcriptome single‐cell reads were mapped to the reference genome (GRCh 38), and count matrices were generated using Cell Ranger (10× Genomics, version 6.0.1) with default parameters. Single‐cell sequencing data from two individual 10× Chromium lanes were combined using the aggregation function (cellranger aggr). Data normalization and downstream analysis were performed with the R package Seurat (version 4.0.1). Based on the inflection point per cell barcode, a threshold of 1,000 detected features per cell was set to filter out empty droplets and cells with low complexity. The count matrix was filtered for genes detected in at least 10 cells. For each cell, the UMI counts of each gene were divided by the total number of counts and multiplied by a scaling factor (10,000), after which they were log‐transformed. Guide RNA enrichment libraries were processed via fba (Duan & Hon, 2021; version 0.0.11) and mapped to the custom gRNA library, allowing two mismatches. The Seurat object was filtered for cells assigned to a particular gRNA, and cells with a strong CRISPRa phenotype were determined using Seurat's Mixscape function. Differential gene expression analysis was performed for each CRISPRa group with DESeq2 (version 1.32.0) using cells assigned to the NT group as control. The top 50 differentially expressed genes were selected (ranked by the adjusted P‐value) from each overexpression group and merged together to create the gene expression signature list. For Fig 1F, functionally related differentially expressed genes were labeled based on overrepresented terms retrieved from the Gene Ontology, the Reactome, the KEGG, and the Molecular Signatures databases and based on enrichment analysis performed with the R package clusterProfiler (version 4.0.2).

### SLC‐focused CRISPR‐based screens for selenium‐relevant transporters

SK‐N‐DZ were transduced with the SLC‐focused CRISPR‐based library in duplicate at an MOI of 0.5. A total of 10 million cells were transduced for each replicate to achieve a representation of > 1,000. After recovery for 3 days, the cells were selected with 0.5 μg/ml puromycin and were maintained with 500 nM Lip‐1 for 5 days. After selection, for each replicate, the cells were split into five groups, which were treated with 20 nM Na_2_SeO_3_, 20 nM Selenocystiene, 200 nM Selenomethione, 50 μM ßME, and 500 nM Lip‐1, respectively. The cells were passaged, representing > 1,000 cells per gRNA throughout the screen. Cell pellets were harvested on Day 14 of treatment. The genomic DNA was extracted using a commercial kit (QIAGEN‐DNeasy) following the manufacturer's instructions. Libraries were sequenced as a multiplexed pool on a Nextseq500 (8 cycles index 2 [i5], 8 cycles index 1 [i7], 75 cycles Read 1, 75 cycles Read 2).

### Generation of *LRP8*‐knockout cell lines

SK‐N‐DZ cells were transduced with lentiviral particles encoding LRP8 gRNAs (lentiCRISPRv2 blast, Addgene plasmid #98293 was a gift from Brett Stringer). After recovery and selection with blasticidin (10 μg/ml), cells were subcloned via limiting dilution. Clonal cell lines were established and tested for LRP8 expression by Western blot. Two clonal cell lines were selected for further experiments.

NHO2A cells were transduced with lentiviral particles encoding murine Lrp8 gRNAs (lentiCRISPRv2 blast, Addgene plasmid #98293 was a gift from Brett Stringer). After selection with blasticidin (10 μg/ml) and recovery, the cell lines were used for further experiments.

Clonal HT1080 and A375 cells constitutively expressing Cas9 were transfected with plasmids encoding gRNAs (pKLV2‐U6gRNA5(BbsI)‐PGKpuro2AmAG‐W, Addgene plasmid #67976 was a gift from Kosuke Yusa) targeting introns flanking *LRP8* exon 9. After selection with puromycin (1 μg/ml), cells were grown by limiting dilution. Clonal cell lines were established and tested by genotyping PCR and Western blot. Two clonal cell lines were selected for further experiments and subsequent CRISPR‐based screens.

### SLC‐focused CRISPR‐based screens in *LRP8*‐KO and wild‐type

*LRP8*‐KO and WT cell lines were transduced with the SLC‐focused CRISPR‐based library at an MOI of 0.5. A total of 10 million cells for each cell line were transduced, achieving > 1,000 cells per gRNA. After recovery and selection with 1 μg/ml puromycin, the *LRP8*‐KO and ‐WT cell lines were split and maintained at the gRNA representation of > 1,000. Cell pellets were harvested after 2 weeks of passaging. The genomic DNA was extracted with a commercial kit (QIAGEN‐DNeasy) following the manufacturer's instructions. Libraries were multiplexed and sequenced on a Nextseq 500 (8 cycles index 2 [i5], 8 cycles index 1 [i7], 75 cycles Read 1, 75 cycles Read 2). The mapping of raw sequencing reads to the reference library, and computing of enrichment scores and *P*‐values were processed using the MaGeCK pipeline. The MaGeCKFlute was used for the identification of gene hits and associated pathways.

### Cell lines generation for a competition assay

Human neuroblastoma cell lines (SK‐N‐DZ and SH‐EP MYCN‐ER) expressing a lentiviral construct carrying *SLC7A11* cDNA or an empty vector were generated using the methods described above. Briefly, lentiviral constructs were transduced into the corresponding cell line labeled with mScarlet or eGFP. The combination of construct/fluorophore is illustrated in the figures present in the main text. Established cell lines were counted and mixed at a 1:1 ratio, and the distribution was monitored during the period indicated in the main text. Rescue experiments were carried out using a combination of ßMe (50 μM) and Erastin (2 μM).

### Analysis of cell viability

The impact of various compounds on cell viability was analyzed using the CellTiter‐Glo (CTG) assay (Promega). To determine changes in cell viability, 3,000 cells were seeded in the complete medium in 96‐well plates (Greiner Bio One) 24 h before the treatment. Cells were then treated for 72 h with the indicated concentration of compounds. Cell viability was analyzed using the CTG assay following the manufacturer's instructions. For *in vitro* clonogenic assays, 200 cells were seeded in a 12‐well plate for 14 days with each particular experimental condition, and colonies were stained with 1 ml 0.01% (w/v) crystal violet.

### Analysis of lipid peroxidation

Approximately 10^5^ cells were seeded in six‐well plates. Before lipid peroxidation was analyzed, the medium was removed, C11‐BODIPY (wavelength: 581/591 nm) diluted in Hank's Balanced Salt Solution (HBSS; Gibco), and added to wells at a final concentration of 4 μM. After 15‐min staining at 37°C inside the tissue culture incubator, cells were harvested gently, and lipid peroxidation levels were immediately analyzed using a BD FACS Aria™ III cell sorter.

### Transient siRNA‐mediated gene knockdown

SK‐N‐DZ cells were seeded in 12‐well plates (200,000 cells/well) and 24 h later transiently transfected with a mix of RNAiMax (0.04 μl/well; Thermo Fisher Scientific) and 0.01 μM/well of siRNA following the manufacturers' instructions.

### Selenium speciation analyses

We measured total selenium by inductively coupled plasma sectorfield mass spectrometry (ICP‐of‐MS), and the selenium species selenite (Se‐IV), selenate (Se‐VI), selenomethionine‐bound selenium (Se‐MET), selenocystine‐bound selenium (Se‐Cys), thioredoxin reductase‐bound selenium (Se‐TrxR), glutathione‐peroxidase‐bound selenium (Se‐GPx), selenoprotein‐P‐bound selenium (SELENOP), and albumin‐bound selenium (Se‐HSA) using ion exchange chromatography (IEC) coupled with ICP‐dynamic reaction cell mass spectrometry (ICP‐DRC‐MS) in analogy to methodologies previously established (Solovyev *et al*, 2013). The experimental settings for ICP‐sf‐MS (ELEMENT II, Thermo Scientific, Bremen Germany) were: radio frequency power: 1260 W, plasma gas flow: 16 l Ar/min auxiliary gas flow: 0.85 l Ar/min, nebulizer gas flow: 1.085 l Ar/min, daily optimized, dwell time 300 ms, ions monitored: 77Se, 78Se, high‐resolution mode. For the speciation of selenium compounds, we used the hyphenated system from Perkin Elmer (Rodgau, Germany) comprising a NexSAR gradient HPLC pump, autosampler, and NexION 300 D ICP‐DRC‐MS, completely controlled by Clarity software. The separation column for species separation was an ion exchange pre‐ and analytical column AG‐11 + AS‐11 (250 × 4 mm I.D.) from Thermo Dionex (Idstein, Germany). The sample volume was 50 μl. The mobile phases and chromatographic gradient were previously published (Solovyev *et al*, 2013). Briefly, the flow rate was 0.80 ml/min. The experimental settings for ICP‐DRC‐MS were: radio frequency power: 1250 W, plasma gas flow: 15 l Ar/min auxiliary gas flow: 1.05 l Ar/min, nebulizer gas flow: 0.92 l Ar/min, daily optimized, dwell time 300 ms, ions monitored: 77Se, 78Se, 80Se, DRC reaction gas: CH4 reaction at 0.58 ml/min, DRC rejection parameter *q*: 0.6. Five‐point calibration curves from 0 to 5,000 ng/l were linear with *r* ^2^ for the three Se isotopes being better than 0.999881. Data files from selenium chromatograms were processed with Clarity software for peak area integration.

### Generation of HepG2 conditional medium and SELENOP enrichment

HepG2 cells were seeded to a 15‐cm^2^‐round dish with standard DMEM medium (10% FBS^+^ 1% Pen/Strep). The medium was changed to FBS‐free medium supplemented with 50 or 200 nM Na_2_SeO_3_ when HepG2 cells achieved ~70–80% confluent. After 48 h incubation, the medium was collected and briefly spined down to clarify the supernatant. The supernatant was transferred to the centrifugal filter (Amicon Ultra‐15, 30K. UFC903024) and centrifuged for 10 min at 3,000 *g*. After the supernatant was centrifuged through the centrifugal filter, 2 ml of FBS‐free medium was used to resuspend the concentratred fraction from the filter. HT1080‐Cas9 LRP8 WT/KO and SK‐N‐DZ cells were plated and treated with 10 nM Na_2_SeO_3_ or HepG2 concentrated medium in six‐well plates in the absence of FBS. After 24‐h incubation, the cell lysates were collected.

### Analyses of pediatric cohorts of neuroblastoma samples

RNA sequencing of 498 neuroblastoma cases was performed as described previously (Zhang *et al*, 2015). In short, mRNA purification was performed using the Dynabeads mRNA Purification Kit (Invitrogen), and library construction was completed according to the standard TruSeq protocol. Clusters were generated according to the TruSeq PE cluster Kit version 3 reagent preparation guide (for cBot‐HiSeq/HiScanSQ). Paired‐end sequencing with 100 bp read length was performed on the Illumina HiSeq 2000 platform. Raw data processing, read mapping, and gene expression quantification were done using the Magic‐AceView analysis pipeline and AceView transcriptome reference (http://www.aceview.org) as described previously (Zhang *et al*, 2015). Genes with generally low read counts were removed using R (v4.1.1) and the function “filterByExpr” in R‐package edgeR (v3.34.1). Differential gene expression analysis was done using the empirical Bayes method implemented in the R‐package limma (v3.48.3).

### *In vivo* orthotopic mouse experiments

All studies involving mice and experimental protocols were conducted in compliance with the German Cancer Center Institute guidelines and approved by the governmental review board of the state of Baden‐Wuerttemberg, Regierungspraesidium Karlsruhe, under the authorization number G‐176/19, followed the German legal regulations. Mouse strains used in the study: NOD.Cg‐Prkdc^scid^Il2rgtm1^Wjl^/SzJ (NSG, JAX stock #005557). Female mice, 3–4 months of age, were used for experiments. Mice have been housed in individually ventilated cages under temperature and humidity control. Cages contained an enriched environment with bedding material. To generate orthotopic mouse models for neuroblastoma, 2 × 10^5^ SK‐N‐DZ cells were transplanted into the right adrenal gland after the surgical site was prepared. Cells were resuspended in a 1:1 (vol/vol) mix of growth factor‐reduced matrigel (Corning) and PBS. Overall, 20 μl of this cell suspension was injected into the right adrenal gland of anesthetized mice. After tumor cell transplantation, we monitored the mice for evidence of tumor development by bioluminescent signal using an IVIS Spectrum Xenogen device (Caliper Life Sciences). We observed a clear signal from the tumors 1 week after injecting 2 × 10^5^ SK‐N‐DZ cells. For liproxstatin (Lip‐1), we used 10 mg/kg/day for the first 5 days. Lip‐1 treatment (every second day) continued in one group for another 2 weeks. Animals' health was monitored daily, and mice were euthanized as soon as they reached the abortion criteria defined in the procedure. The sample size was calculated with the help of a biostatistician using R version 3.4.0. Assumptions for power analysis were as follows: *α* error, 5%; *β* error, 20%. Mice were randomized into treatment groups before treatment. If animals had to be sacrificed before the predefined endpoint (due to weight loss or other termination criteria), they were excluded from any downstream analyses. All animal experiments were blinded during experiments and follow‐up assessments.

## Author contributions

**Hamed Alborzinia:** Conceptualization; supervision; funding acquisition; writing – original draft; writing – review and editing. **Zhiyi Chen:** Data curation; formal analysis; investigation; writing – original draft; writing – review and editing. **Umut Yildiz:** Formal analysis; investigation; data curation; writing – original draft. **Florencio Porto Freitas:** Investigation. **Felix C E Vogel:** Investigation. **Julianna Patricia Varga:** Investigation. **Jasmin Batani:** Investigation. **Christoph Bartenhagen:** Data curation; formal analysis; visualization. **Werner Schmitz:** Data curation; formal analysis; methodology. **Gabriele Büchel:** Resources. **Bernhard Michalke:** Resources; methodology. **Jiashuo Zheng:** Investigation. **Svenja Meierjohann:** Resources; methodology. **Enrico Girardi:** Resources. **Elisa Espinet:** Investigation. **Andrés F Flórez:** Investigation. **Ancely Ferreira dos Santos:** Investigation. **Nesrine Aroua:** Investigation. **Tasneem Cheytan:** Investigation. **Julie Haenlin:** Methodology. **Lisa Schlicker:** Investigation. **Thamara N Xavier da Silva:** Investigation. **Adriana Przybylla:** Methodology. **Petra Zeisberger:** Methodology. **Giulio Supert‐Furga:** Resources. **Martin Eilers:** Resources. **Marcus Conrad:** Methodology. **Marietta Fabiano:** Investigation; methodology. **Ulrich Schweizer:** Resources; methodology. **Matthias Fischer:** Data curation; formal analysis. **Almut Schulze:** Supervision; funding acquisition. **Andreas Trumpp:** Conceptualization; funding acquisition; writing – review and editing. **José Pedro Friedmann Angeli:** Conceptualization; supervision; funding acquisition; writing – original draft; project administration; writing – review and editing.

## Disclosure and competing interests statement

The authors declare the following financial interests/personal relationships, which may be considered as potential competing interests: MC is the cofounder of ROSCUE THERAPEUTICS GmbH and author of a patent application related to ferroptosis. GS‐F is coauthor of patent applications related to SLCs, cofounder of Solgate GmbH, and the Academic Project Coordinator of the IMI grants RESOLUTE and Resolution in partnership with Pfizer, Novartis, Bayer, Sanofi, Boehringer‐Ingelheim and Vifor Pharma. The GS‐F laboratory receives funds from Pfizer. All other authors declare no other relevant conflicts of interest.

## Supporting information



Appendix S1

Dataset EV1

Dataset EV2

Dataset EV3

Source Data for Figure 1

Source Data for Figure 2

Source Data for Figure 3

Source Data for Figure 4

## Data Availability

Raw screening data generated from the genome‐wide and validation CRISPRa screens are uploaded to the Sequence Read Archive under the accession code PRJNA866989 (https://www.ncbi.nlm.nih.gov/bioproject/PRJNA866989). Raw and processed data from the CROP‐seq experiment is deposited on the Gene Expression Omnibus (accession number GSE210762; http://www.ncbi.nlm.nih.gov/geo/query/acc.cgi?acc=GSE210762).
