# Supplementary material for: LRP8‐mediated selenocysteine uptake is a targetable vulnerability in MYCN‐amplified neuroblastoma
Source: EMBO Mol Med. 2023 Jul 12;15(8):e18014. doi: 10.15252/emmm.202318014 (PMC10405063; doi:10.15252/emmm.202318014)
Supplement: Supplementary file 1 — Appendix S1 [file EMMM-15-e18014-s006.pdf]

Table of Contents

Content	Page
Appendix Figure S1	2
Appendix Figure S2	4
Appendix Figure S3	6
Appendix Figure S4	8
Appendix Figure S5	10
Appendix Figure S6	11

Appendix Figure S1

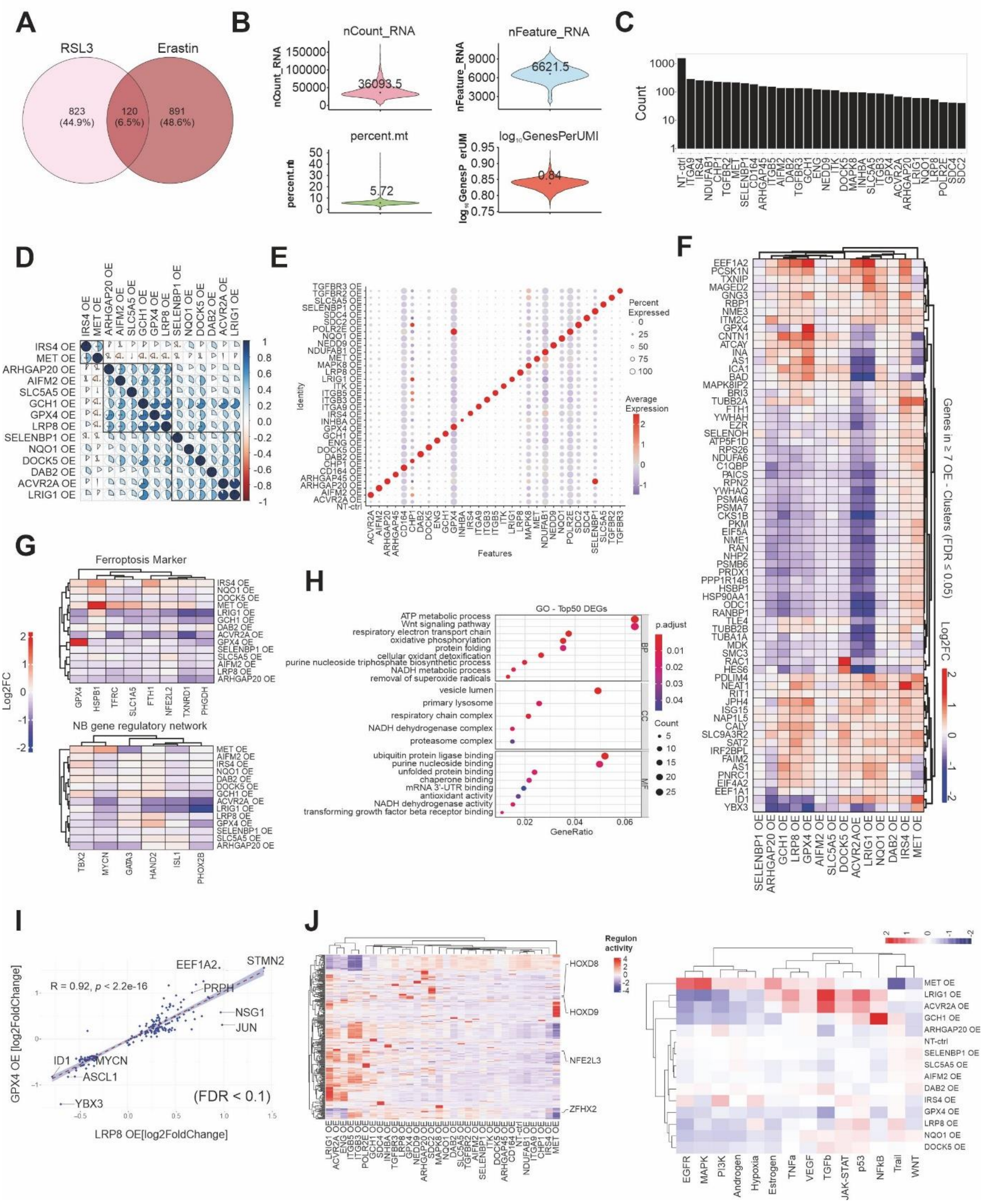

Appendix Figure S1.

A, Venn diagram comparing CRISPRa hits identified in the genome-wide ferroptosis resistance screens in the SK-N-DZ neuroblastoma cell line upon RSL3- and Erastin-induced ferroptosis (FDR ≥ 0.05, AvgLogFC > 0).

B, Top left: number of unique molecular identifiers (UMIs) per cell in the scCRISPRa sample. Top right: number of detected genes per cell. Bottom left: percentage of mitochondrial reads detected—bottom

right: number of genes per UMI (log-transformed) reflecting library complexity. The median values are printed and highlighted by a dot.

C, In the single-cell experiment, the number of cells (y-axis, in log scale) is assigned to each scoring hit (indicated on the x-axis). OE: overexpression, NT ctrl: cells assigned to non-targeting control gRNAs.

D, Pairwise comparison of transcriptome similarities of validated CRISPRa clusters and genes derived from the gene expression signature list (based on Spearman correlation). Color intensities and ratios of the pie chart indicate relative overlap. Rows and columns are hierarchically ordered, and related groups are marked with a blue rectangle. ***P < 0.001, **P < 0.01, *P < 0.05.

E, Dot plot showing expression of target genes (x-axis) in each CRISPRa cluster (y-axis). The size of the dots reflects the fraction of cells within a particular cluster for which the gene expression was detected. OE: overexpression. NT ctrl: a cluster of cells assigned to non-targeting control gRNAs. F, Changes in gene expression following CRISPRa (clusters represented by each row) of genes that were detected as significantly differentially expressed (FDR ≤ 0.05) in at least seven of the 14 scorings CRISPRa clusters. Columns and rows were hierarchically clustered based on Pearson correlation.

G, Changes in expression of ferroptosis-related genes (upper) and transcriptional core regulatory circuits maintaining cell state in MYCN-amplified neuroblastoma derived from(Durbin et al, 2018) (lower) that were detected as significantly differentially expressed in at least one of the CRISPRa clusters represented by each row.

H, Gene Ontology (GO) term enrichment analyzis was conducted on the signature gene list derived from the top 50 genes showing the most significant differential expression in each scoring CRISPRa cluster. Selected terms are shown up to FDR ≤ 0.05. BP: biological process, CC: cellular compartment, MF: molecular function.

I, Scatterplot showing commonly differentially expressed genes in GPX4-OE (normalized expression values indicated on the y-axis) and LRP8-OE cells (x-axis) compared to control cells transduced with non-targeting gRNAs (FDR ≤ 0.1). A linear model was added to the trend line (dashed line in red), and a Pearson correlation coefficient was computed.

J, left: single-cell regulatory network inference and clustering (SCENIC)(Aibar et al, 2017) results are shown in a heatmap. Transcription factor activity scores inferred from the CROP-seq experiment are shown across scoring CRISPRa groups (x-axis) in the primary genome-wide and validation screens. Transcription factors with a positive activity score that overlap in the GPX4- and LRP8-OE groups are highlighted. Right: Heatmap showing inferred pathway activity scores (via PROGENy)(Schubert et al, 2018) from the CROP-seq experiment across scoring CRISPRa groups (y-axis).

Appendix Figure S2

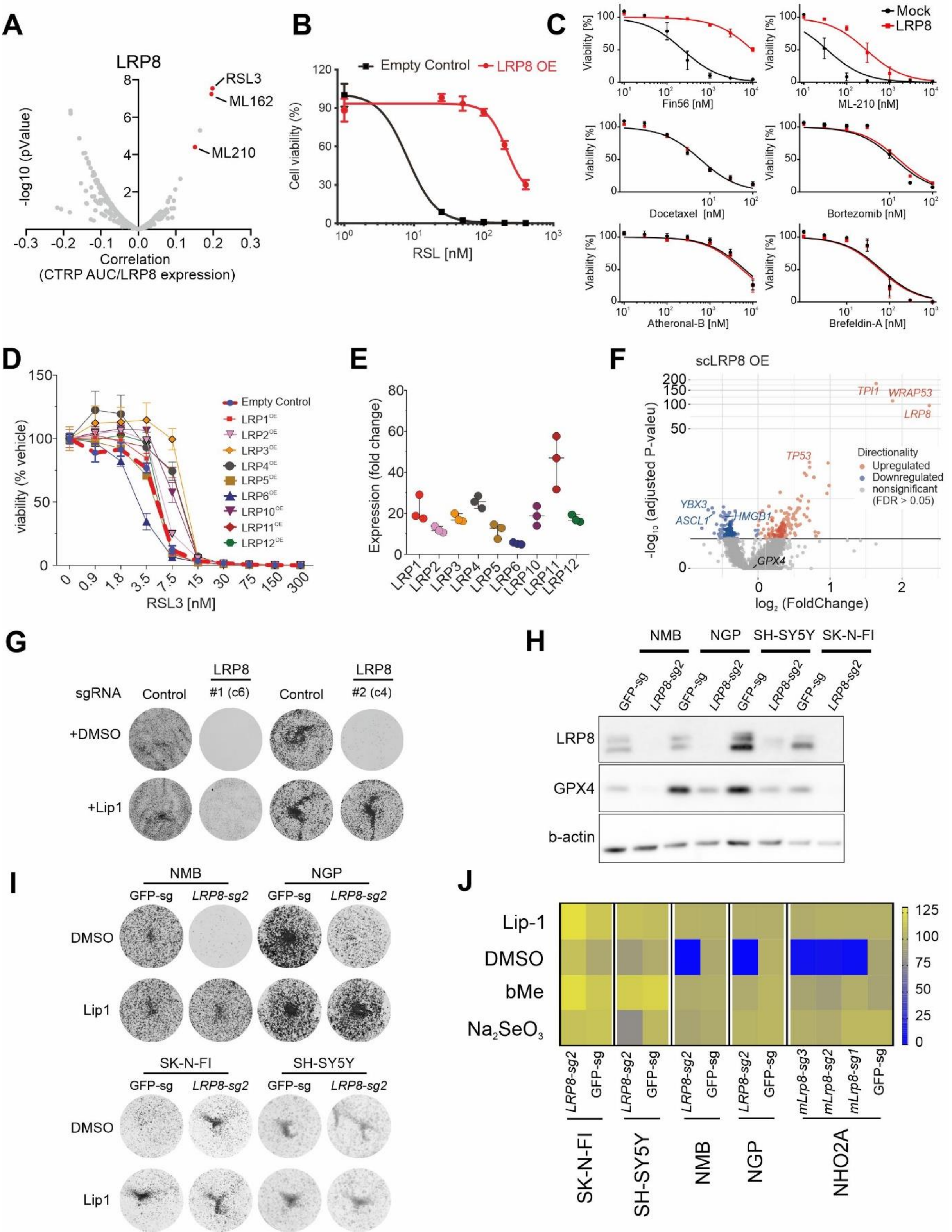

Appendix Figure S2.

A, High expression of LRP8 is correlated with resistance to GPX4 inhibitors in a panel of 1400 cell lines. Data were extracted from the CTRP/depmap database.

B, Dose-dependent toxicity of RSL3 in SK-N-DZ dCas9-SAM cells expressing gRNA targeting the LRP8promoter or a control gRNA. Data are the mean ± s.e.m. of n = 3 wells of a 96-well plate from three independent experiments.

C, Dose-dependent response of SK-N-DZ cells overexpressing an empty vector or human LRP8(hLRP8) using a panel of cytotoxic drugs. Data are the mean ± s.e.m. of n = 3 wells of a 96-well plate from three independent experiments.

D, dose-dependent toxicity of RSL3 in SK-N-DZ dCas9-SAM cell expressing gRNAs targeting different members of the LRP family. Data are the mean ± s.e.m. of n = 3 wells of a 96-well plate from three independent experiments.

E, Expression changes of different LRPs in SK-N-DZ dCas9-SAM cell expressing the respective gRNA. Data are presented as mean ± s.e.m. of n = 3 wells.

F, Volcano-plot showing differentially expressed genes in the LRP8 overexpression cluster compared to non-targeting control cells. Each dot represents a single gene. Mean expression values (log2 foldchange) are plotted against the significance values (log10 transformed adjusted p-values). Significantly upregulated genes are marked in red, while downregulated genes are marked in blue (FDR ≤ 0.05).

G, The clonogenic capacity of two clonal cell lines derived from the SK-N-DZ line in standard medium and medium supplemented with Lip-1 (500 nM).

H, Immunoblot analyzis of LRP8 and GPX4 in MYCN-amplified (NMB and NGP) and non-amplified (SHSY5Y and SK-N-FI) neuroblastoma cells transduced with gRNA targeting Lrp8.

I, The clonogenic capacity of the indicated neuroblastoma cell lines transduced with a gRNA targeting LRP8 in the presence or absence of the ferroptosis suppressor liproxstatin-1 (500nM Lip-1).

J, Heatmap indicating viability measured using Alamar Blue of the indicated cell lines transduced with gRNA expression constructs targeting LRP8 or controls (GFP-sg) in the presence of different ferroptosis inhibitors, including sodium selenite

Appendix Figure S3

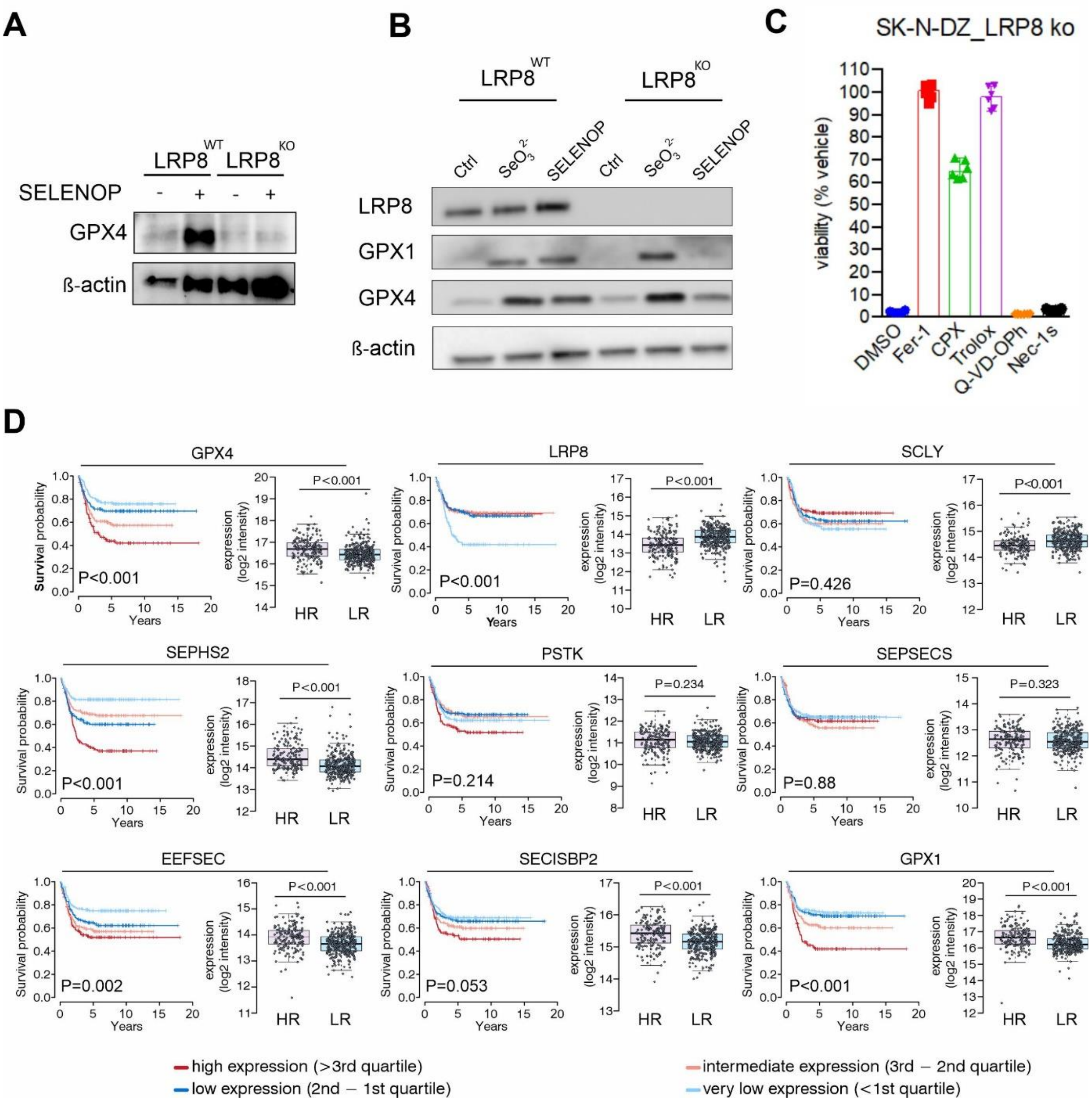

Appendix Figure S3.

A, Immunoblot of GPX4 in SK-N-DZ cell line proficient and deficient for LRP8 exposed for 24 h to SELENOP isolated from HepG2 concentrated supernatants.

B, Immunoblot of GPX1 and GPX4 in HT1080 cell lines proficient and deficient for LRP8 exposed to 10nM selenite or a 1:1 mixture of HepG2 supernatant and FBS-free media for 24h.

C, Impact of different cell death inhibitors (Fer-1, 5 µM; CPX, 500 nM; Trolox, 100 µM; Q-VD-OPh, 10 µM and Nec-1s, 1 µM) on the cell death induced by LRP8 loss. Cells were plated without Lip1 and supplemented with the indicated inhibitors for 72 hours. Viability was assessed using SYTOGreen. Data are the mean ± s.e.m. of n = 6 wells of a 96-well plate from 3 independent experiments.

D, Association between selected genes in the selenium/selenocysteine biosynthetic pathway on KaplanMeier survival analyzis and expression levels (log2 intensity) in low-risk (LR) and high-risk (HR) neuroblastoma patients (n=459). The p-values were calculated using a two-sided Wilcoxon rank-sum test (boxplots, comparison of gene expression between high and low-risk patients) and log-rank test

(Kaplan-Meier curves, pairwise comparisons) and Benjamini-Hochberg corrected. All p-values were adjusted for multiple testing (Benjamini- Hochberg). The centreal band indicates the median. The boxes cover the first and third quartile, with whiskers indicating the minimum and maximum of the data within 1.5× the interquartile range.

Appendix Figure S4

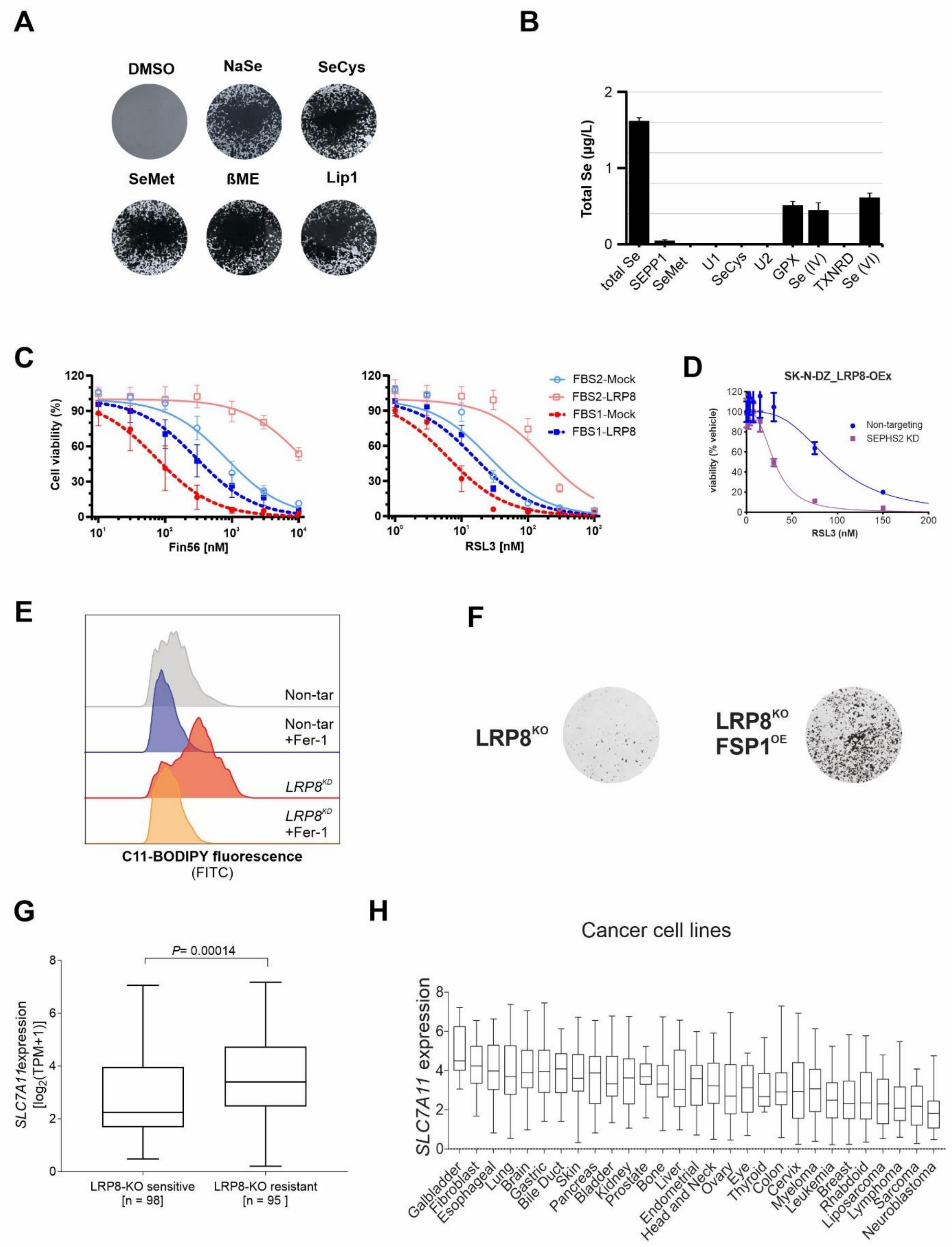

Appendix Figure S4.

A, Clonogenic capacity of SK-N-DZ cells in SELENOP poor FBS. Rescue was carried out using liproxistatin-1 (Lip-1, 500 nM), ?Me (50 µM), NaSe (50 nM), selenomethione (500 nM) and selenocysteine (50 nM).

B, Analyzis of selenium speciation in FBS lacking growth-supporting capacity of SK-N-DZ cells. Data are the mean ± SD of n = 2.

C, Dose-dependent toxicity of FIN56 and RSL3 in SK-N-DZ expressing a lentiviral construct expressing Flag-LRP8 (LRP8) or empty vector control (Mock). Viability assays were performed in the two FBS batches described in (A). Data are the mean ± s.e.m. of n = 3 wells of a 96-well plate from three independent experiments.

D, Dose-dependent toxicity of RSL3 in SK-N-DZ cells overexpressing LRP8 (LRP8-OEx) and cotransfected with siRNA targeting SEPHS2 (SEPHS2KD) or non-targeting control. Data are the mean ± s.e.m. of n = 3 wells of a 96-well plate from three independent experiments.

E, Flow cytometry analyzis of C11-BODIPY oxidation in SK-N-DZ LRP8 knockdown cells (LRP8KD) or

non-targeting controls in the presence and absence of ferrostatin-1 (1 µM).

F, Clonogenic capacity of SK-N-DZ LRP8 knockout cells (LRP8KO) expressing an FSP1 overexpression construct (FSP1OE) or empty vector control.

G, Comparison of SLC7A11 expression (log2(TPM+1)) in cancer cell lines most sensitive to a LRP8 knockout (n = 98) versus cancer cell lines showing least sensitivity (n = 95). Gene expression and CRISPR dependency data retrieved from the cancer dependency map (gene expression release 22q4 public, CRISPR dependency Chronos release 22q4). Two-tailed Mann-Whitney test was used to compute the significance score as indicated. The center line in the box plot indicates the median value. Lower and upper hinges represent the 25th and 75th quantiles and the whiskers denote the 1.5x interquartile range.

H, SLC7A11 expression (log2(TPM+1)) in cancer cell lines grouped by cancer type. Gene expression data retrieved from the depmap database (release 22q4 public; n = 1377). The center lines in the box plots indicate the median values. Lower and upper hinges represent the 25th and 75th quantiles and the whiskers range from the minimum to the maximum value.

Appendix Figure S5

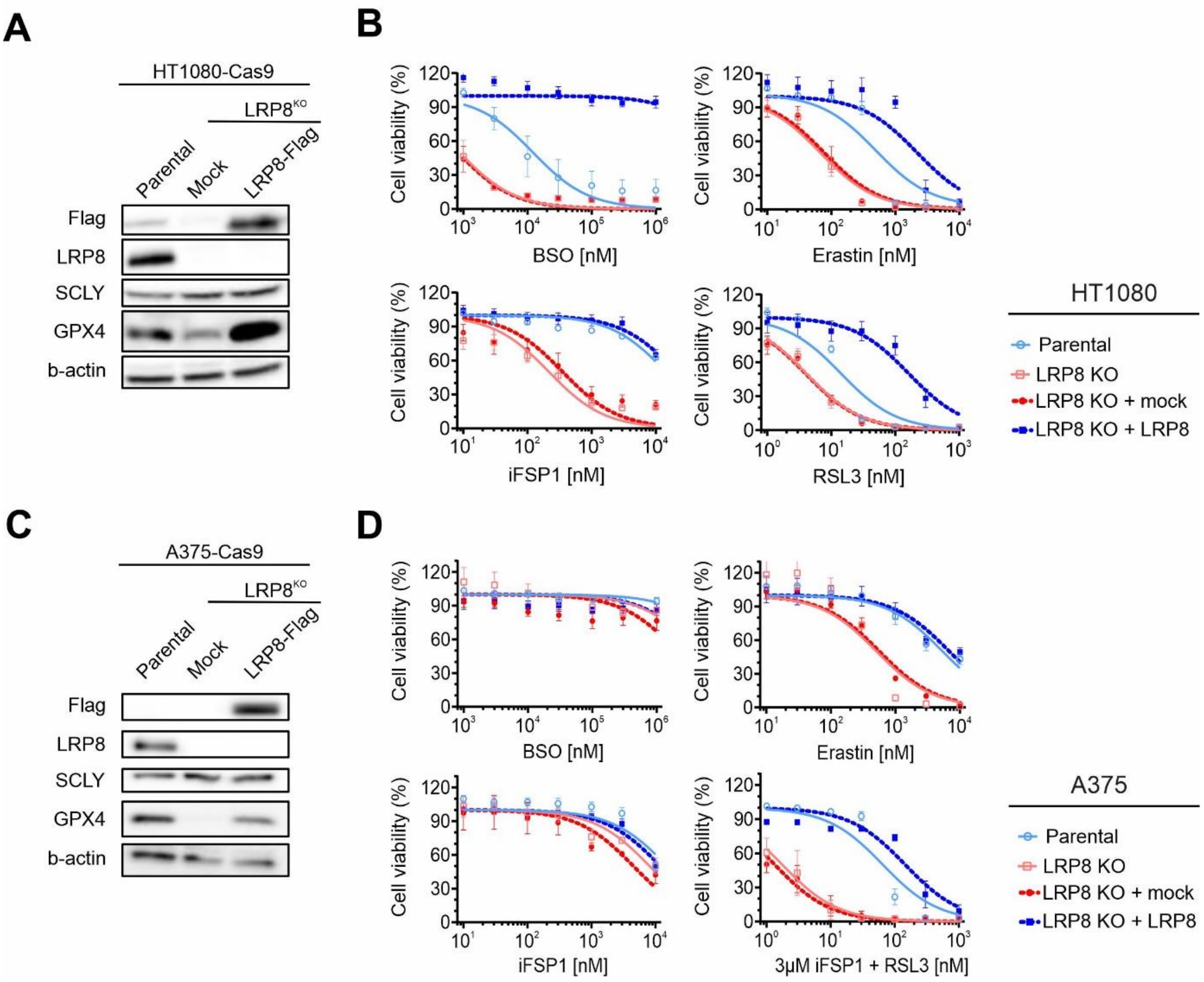

Appendix Figure S5.

A-D A, Immunoblot analyzis of LRP8, SCLY, GPX4, and Flag in LRP8-deficient HT1080 (A) and A375 (C) cells were overexpressing hLRP8-Flag or empty vector (Mock). Parental cell lines only expressing Cas9 are shown as control. Dose-dependent toxicity of the ferroptosis inducers iFSP1, Erastin, BSO, and RSL3 in LRP8-deficient (LRP8 KO) HT1080 (B) and A375 (D) cell lines stably transduced with a vector expressing hLRP8-Flag. Parental cell lines only expressing Cas9 are shown as control. Data are the mean ± s.e.m. of n = 3 wells of a 96-well plate from three independent experiments

Appendix Figure S6

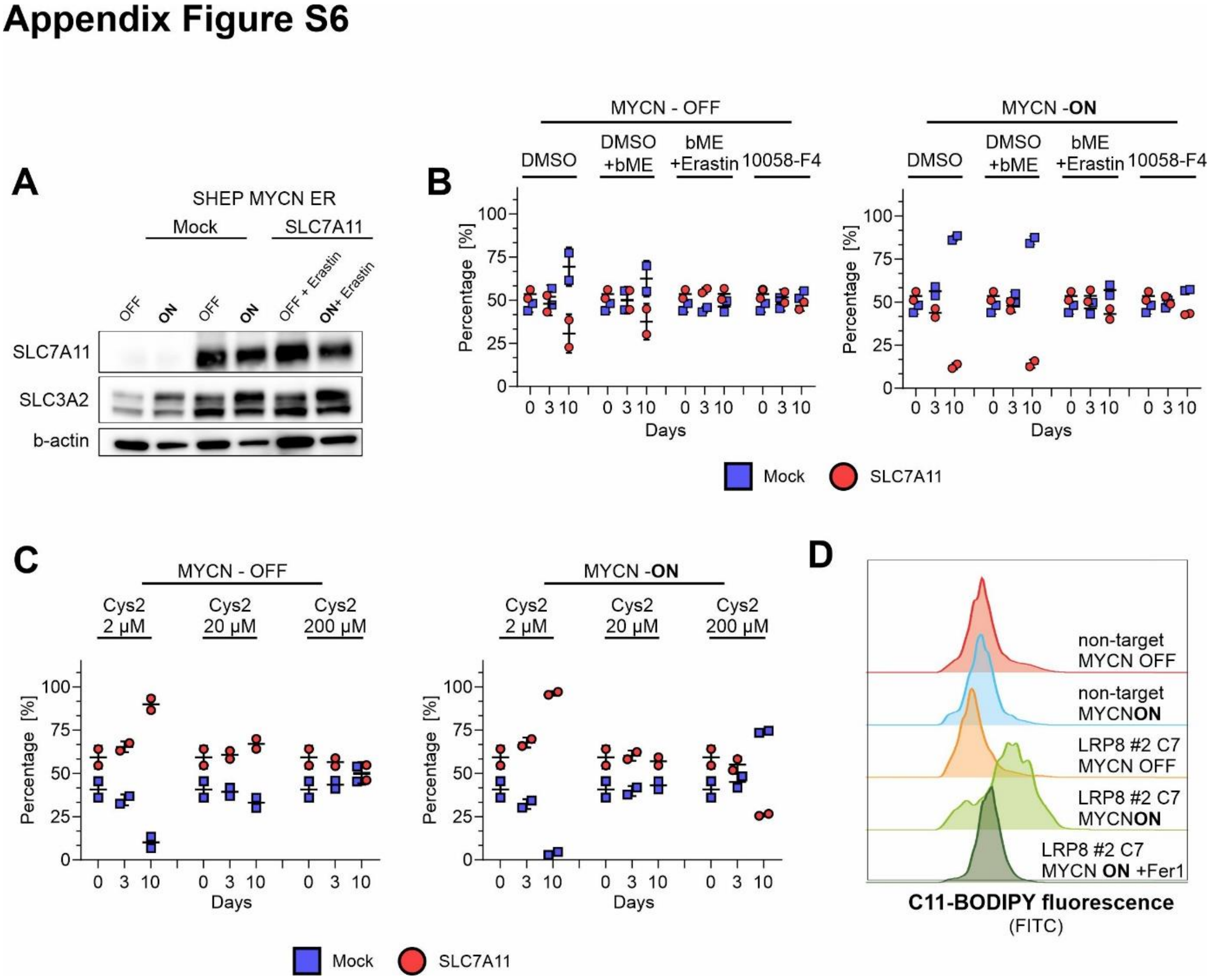

Appendix Figure S6.

A, Immunoblot analysis of SLC7A11 and SLC3A2 in the SHEP MYCN-ERT2 model.

B, Cell competition assay of SHEP MYCN-ERT2 cell line overexpressing SLC7A11 or mock controls. The pharmacological rescues in the presence of 50 µM beta-Mercaptoethanol (ßME), 2 µM Erastin or 20 µM of the MYCN inhibitor 10058-F4. Data are the mean ± SD from two independent experiments.

C, cystine withdrawl rescue. For the experiment, cells were seeded with and without 4OH-Tamoxifen (TAM, 500 nM) at a ratio of 50/50 and 50,000 events were measured via flow cytometry at the depicted time points. Rescue experiments were performed. Bars display percentage of eGFP- (blue) and Scarletcells (red) the means +/- SD of two independent expreriments.

D, Flow cytometry analyzis of BODIPY 581/591 C11 oxidation in SHEP MYCN-ERT2 LRP8 WT and KO activating MYCN activity with Tamoxifen
