# Supplementary material for: LRP8‐mediated selenocysteine uptake is a targetable vulnerability in MYCN‐amplified neuroblastoma
Source: EMBO Mol Med. 2023 Jul 12;15(8):e18014. doi: 10.15252/emmm.202318014 (PMC10405063; doi:10.15252/emmm.202318014)
Supplement: Supplementary file 6 — Source Data for Figure 2 [file EMMM-15-e18014-s009.zip › Figure 2/Fig 2G/Western blot/Fig 2G.pptx]

## Slide 1
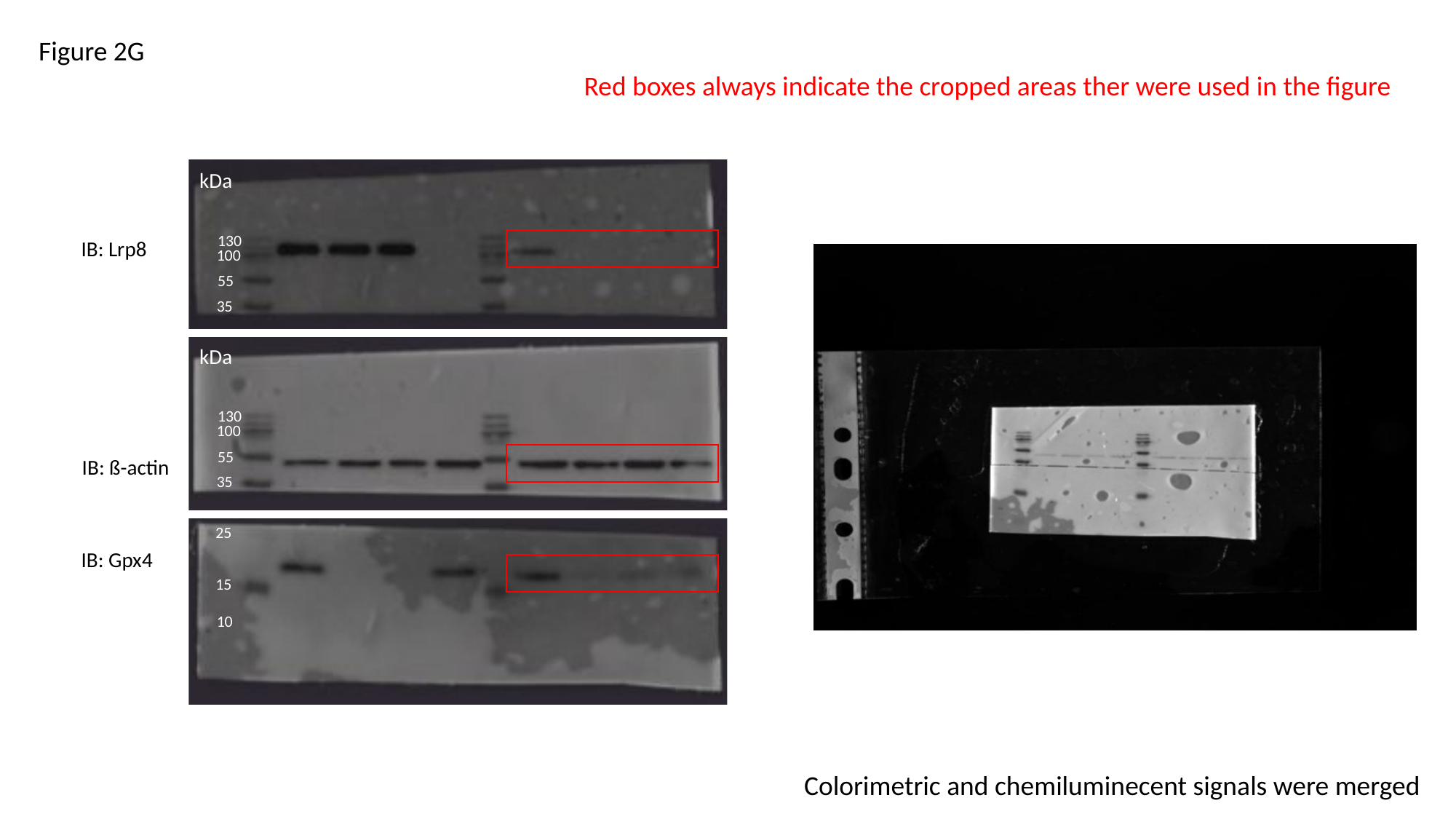

Figure 2G
Red boxes always indicate the cropped areas ther were used in the figure
kDa
130
IB: Lrp8
100
55
35
kDa
130
100
55
IB: ß-actin
35
25
IB: Gpx4
15
10
Colorimetric and chemiluminecent signals were merged

## Slide 2
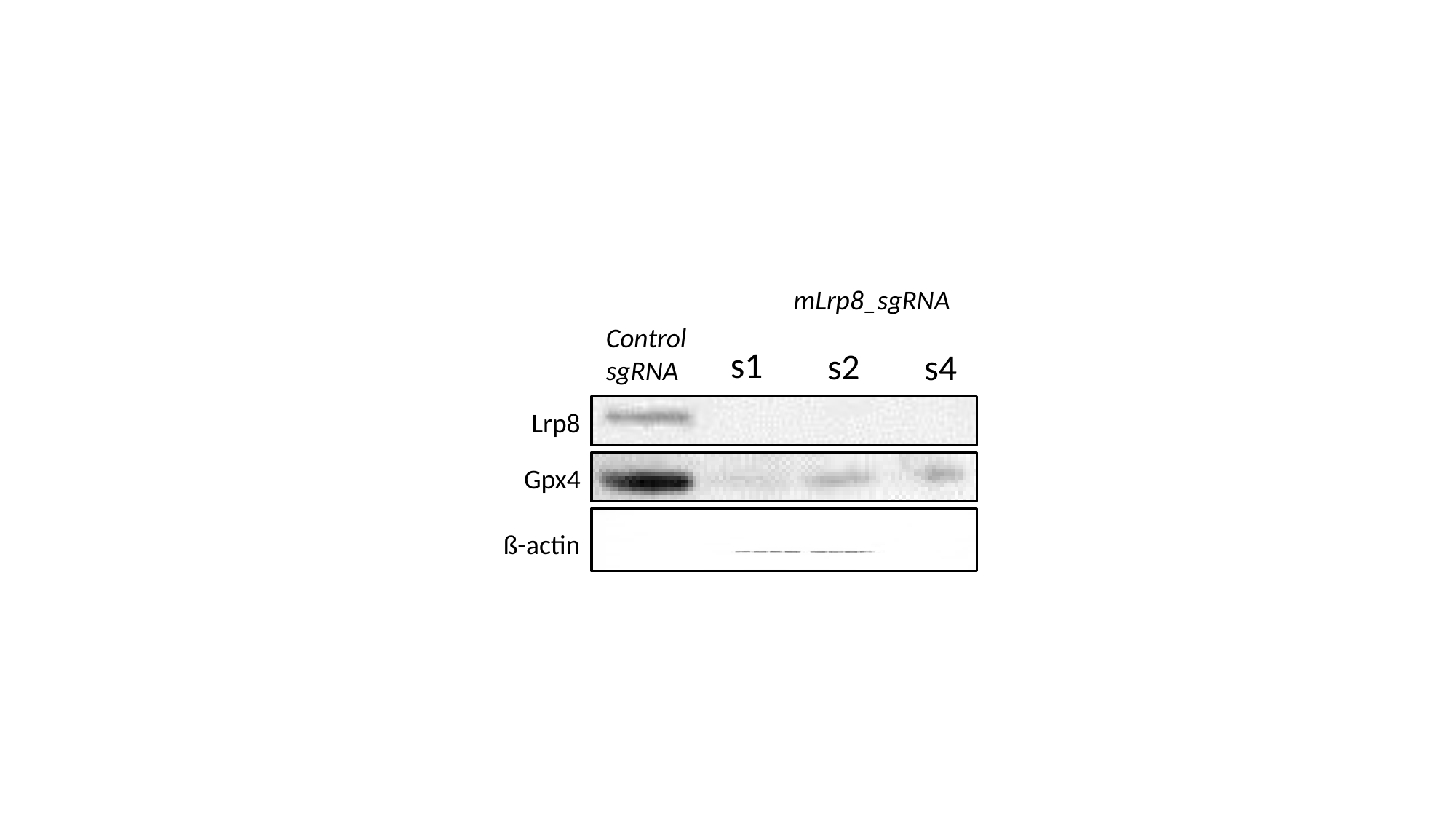

mLrp8_sgRNA
Control
sgRNA
s1
s2
s4
Lrp8
Gpx4
ß-actin
