# Supplementary figures and images for: LRP8‐mediated selenocysteine uptake is a targetable vulnerability in MYCN‐amplified neuroblastoma
Source: EMBO Mol Med. 2023 Jul 12;15(8):e18014. doi: 10.15252/emmm.202318014 (PMC10405063; doi:10.15252/emmm.202318014)

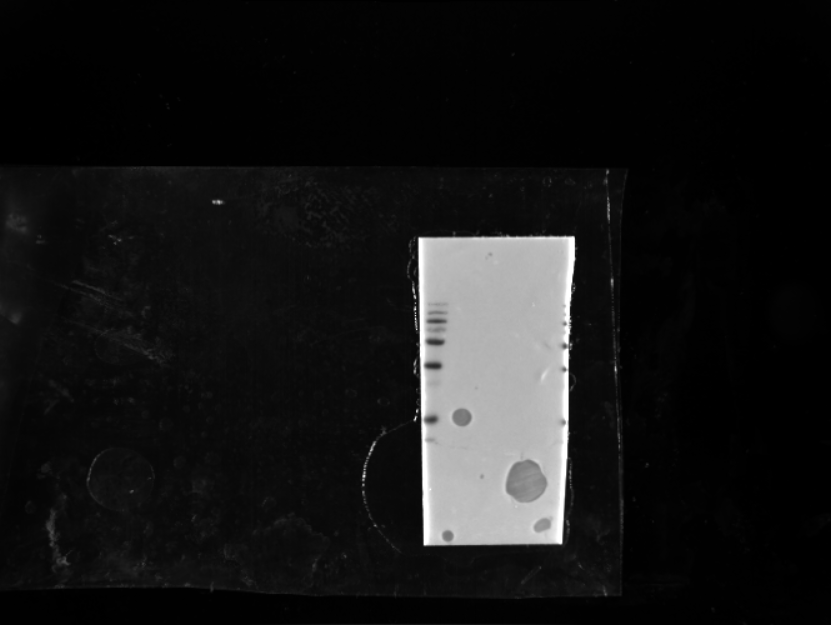

Supplement: Supplementary file 6 — Source Data for Figure 2 [file EMMM-15-e18014-s009.zip › Figure 2/Fig 2A/Fig 2A-LRP8 marker_pub.tif]

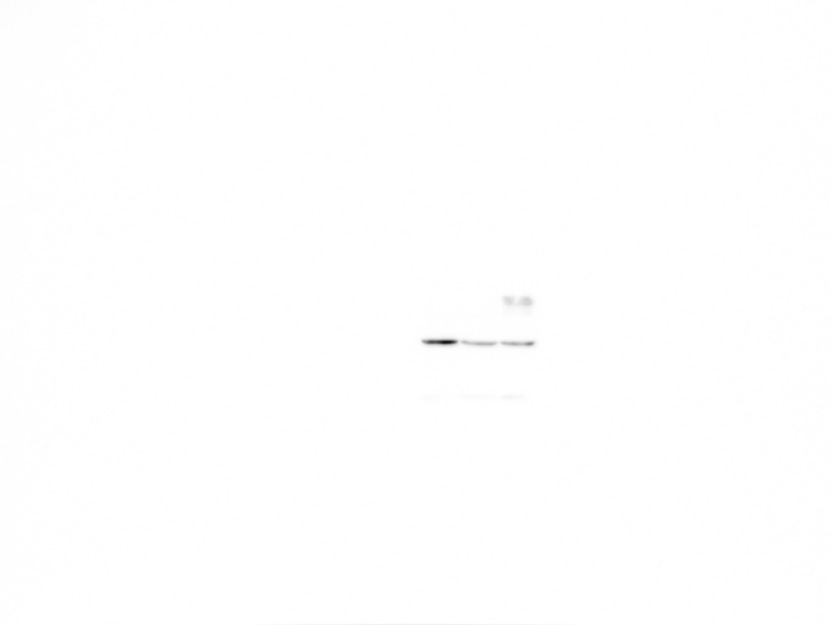

Supplement: Supplementary file 6 — Source Data for Figure 2 [file EMMM-15-e18014-s009.zip › Figure 2/Fig 2A/Fig 2A-actin_pub.tif]

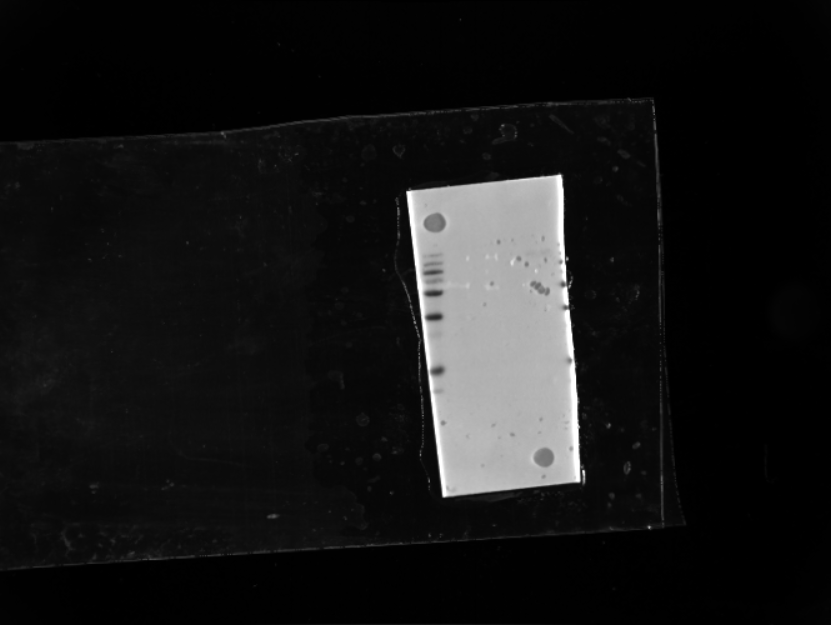

Supplement: Supplementary file 6 — Source Data for Figure 2 [file EMMM-15-e18014-s009.zip › Figure 2/Fig 2A/Fig 2A-GPX4-MARKER_pub.tif]

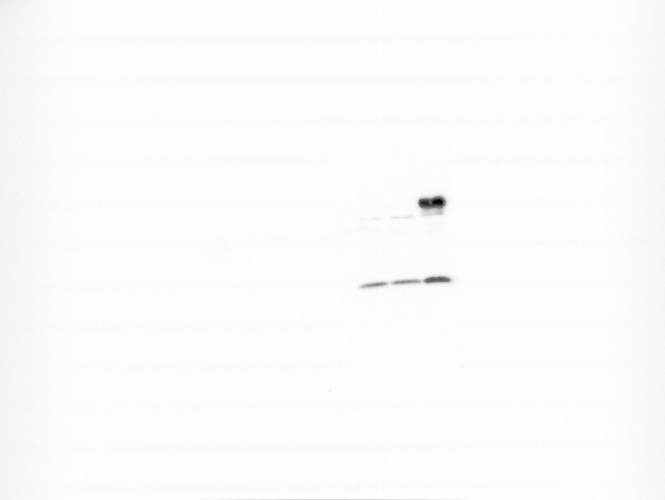

Supplement: Supplementary file 6 — Source Data for Figure 2 [file EMMM-15-e18014-s009.zip › Figure 2/Fig 2A/Fig 2A-GPX4.tif]

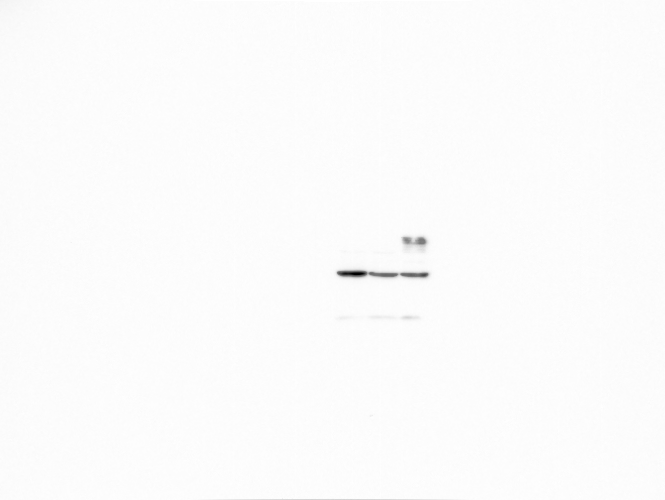

Supplement: Supplementary file 6 — Source Data for Figure 2 [file EMMM-15-e18014-s009.zip › Figure 2/Fig 2A/Fig 2A-actin.tif]

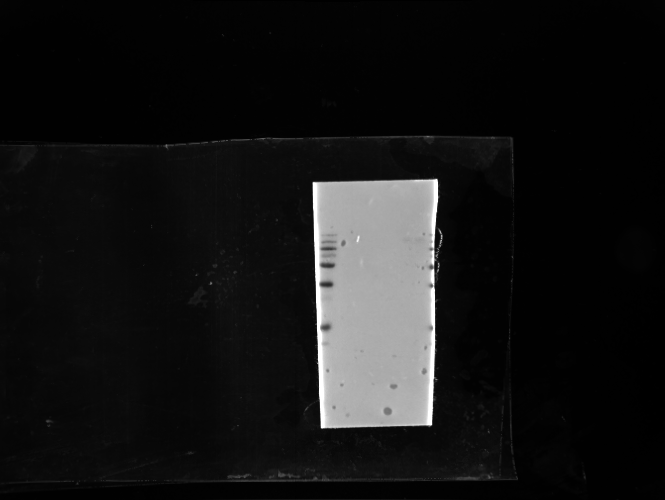

Supplement: Supplementary file 6 — Source Data for Figure 2 [file EMMM-15-e18014-s009.zip › Figure 2/Fig 2A/Fig 2A-actin-marker.tif]

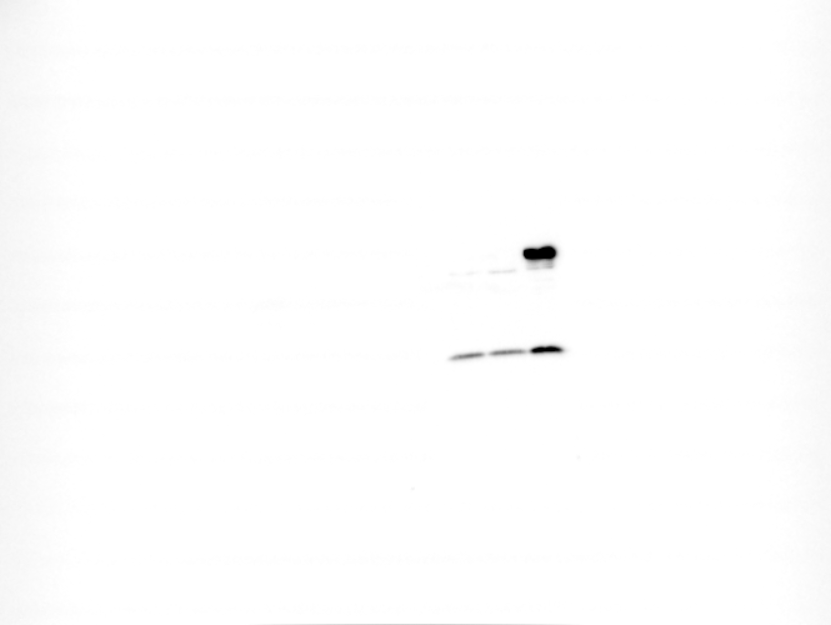

Supplement: Supplementary file 6 — Source Data for Figure 2 [file EMMM-15-e18014-s009.zip › Figure 2/Fig 2A/Fig 2A-GPX4_pub.tif]

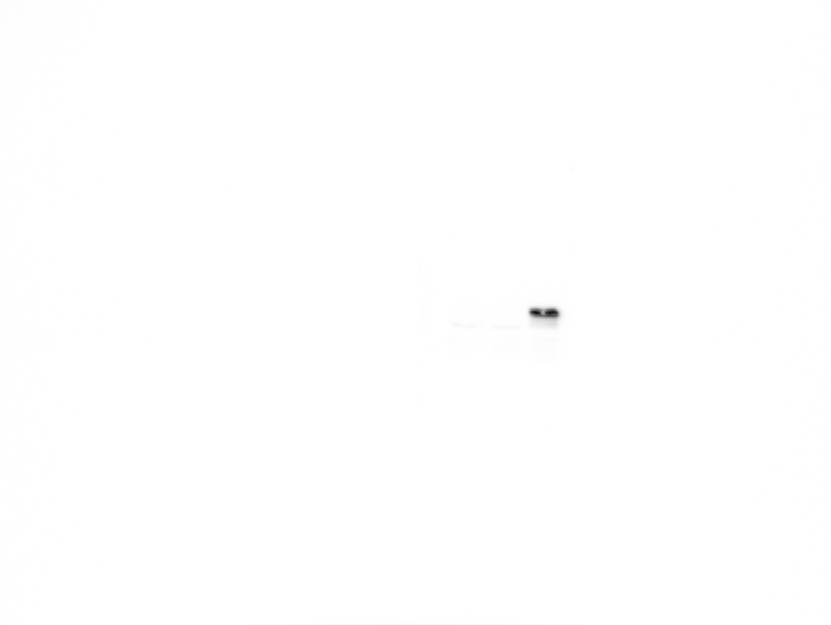

Supplement: Supplementary file 6 — Source Data for Figure 2 [file EMMM-15-e18014-s009.zip › Figure 2/Fig 2A/Fig 2A-LRP8_pub.tif]

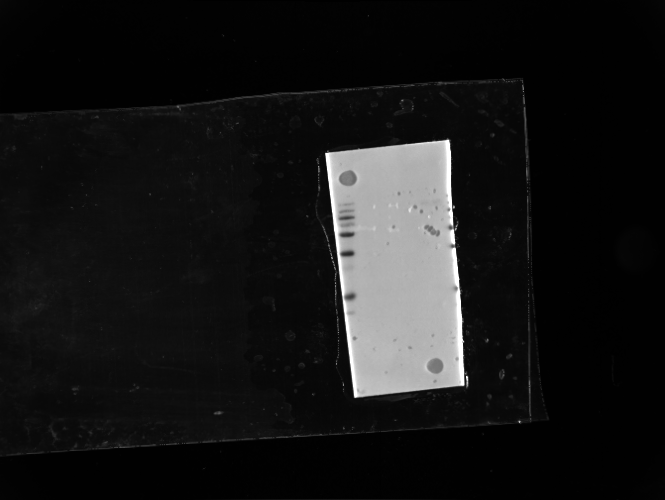

Supplement: Supplementary file 6 — Source Data for Figure 2 [file EMMM-15-e18014-s009.zip › Figure 2/Fig 2A/Fig 2A-GPX4-MARKER.tif]

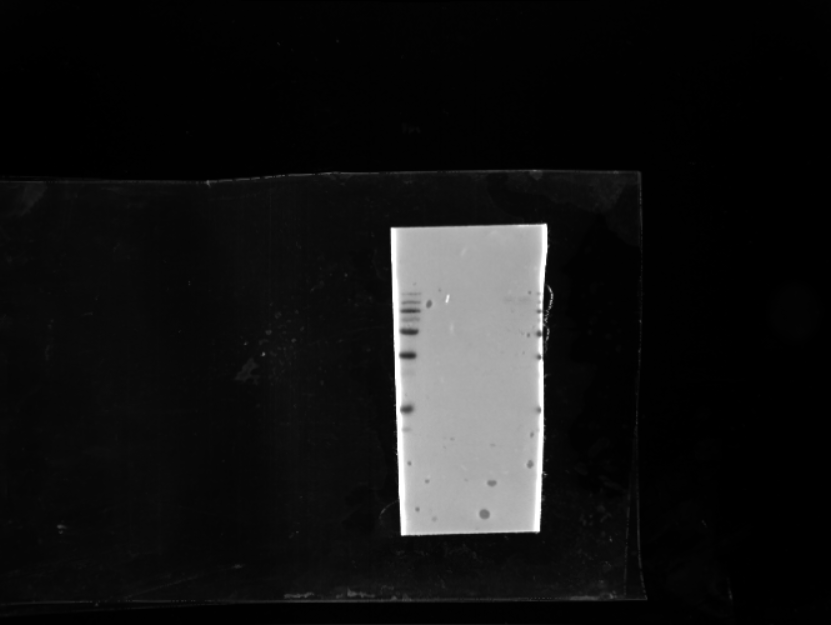

Supplement: Supplementary file 6 — Source Data for Figure 2 [file EMMM-15-e18014-s009.zip › Figure 2/Fig 2A/Fig 2A-actin-marker_pub.tif]

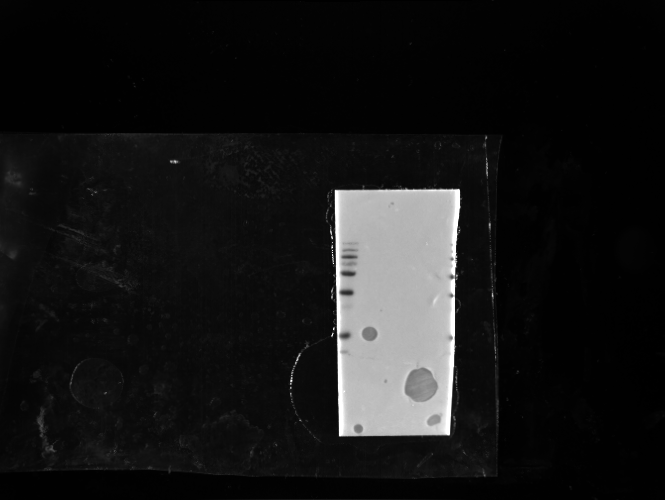

Supplement: Supplementary file 6 — Source Data for Figure 2 [file EMMM-15-e18014-s009.zip › Figure 2/Fig 2A/Fig 2A-LRP8 marker.tif]

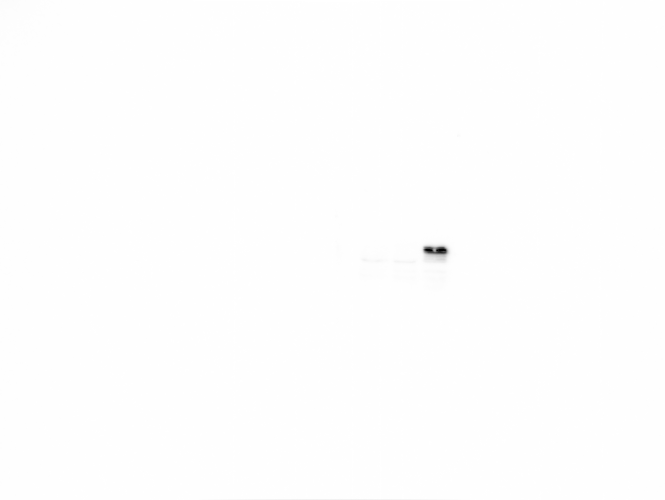

Supplement: Supplementary file 6 — Source Data for Figure 2 [file EMMM-15-e18014-s009.zip › Figure 2/Fig 2A/Fig 2A-LRP8.tif]

## Slide 1
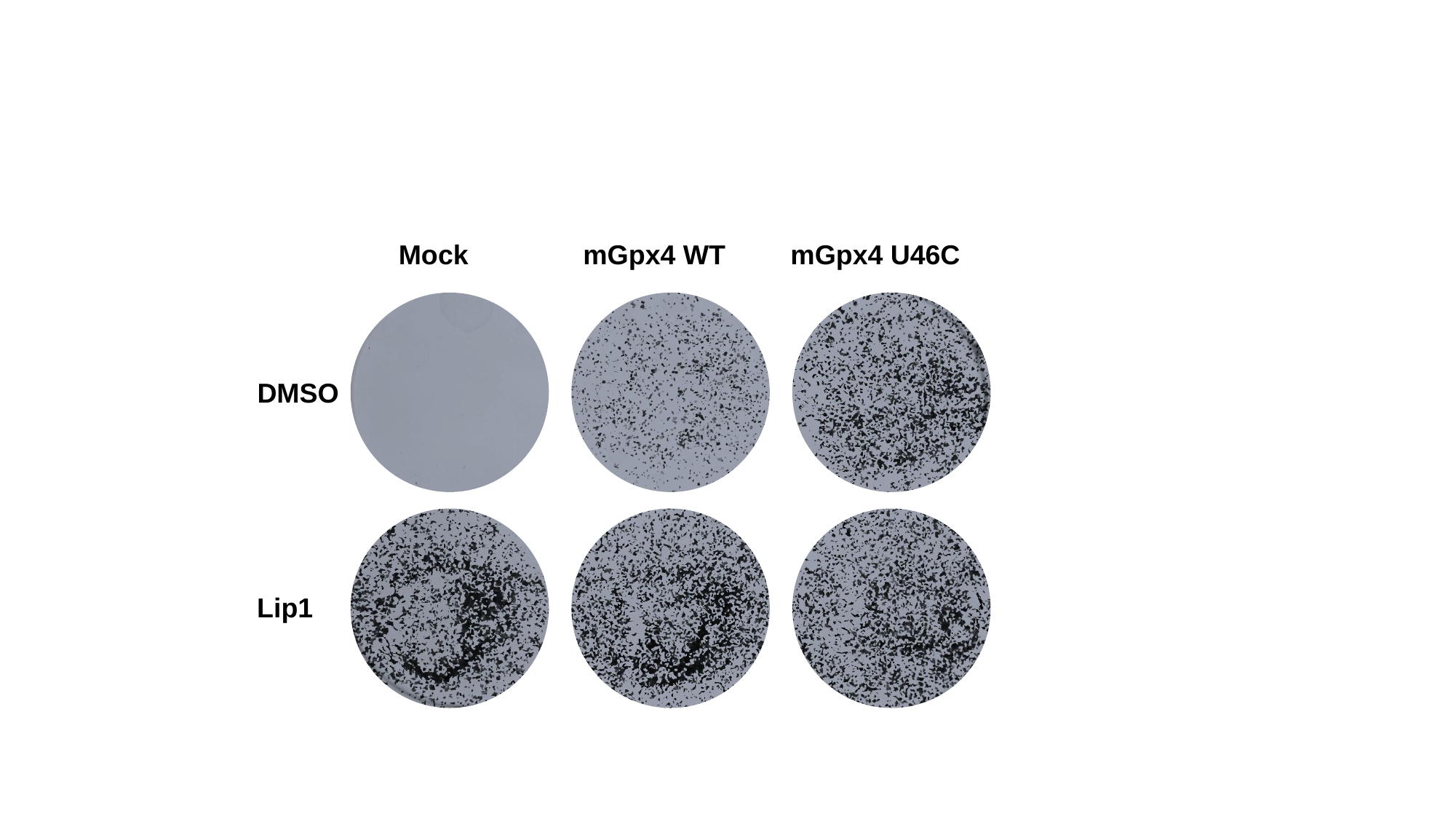

Mock
mGpx4 WT
mGpx4 U46C
DMSO
Lip1

Supplement: Supplementary file 6 — Source Data for Figure 2 [file EMMM-15-e18014-s009.zip › Figure 2/Fig 2I/Fig 2I.pptx]

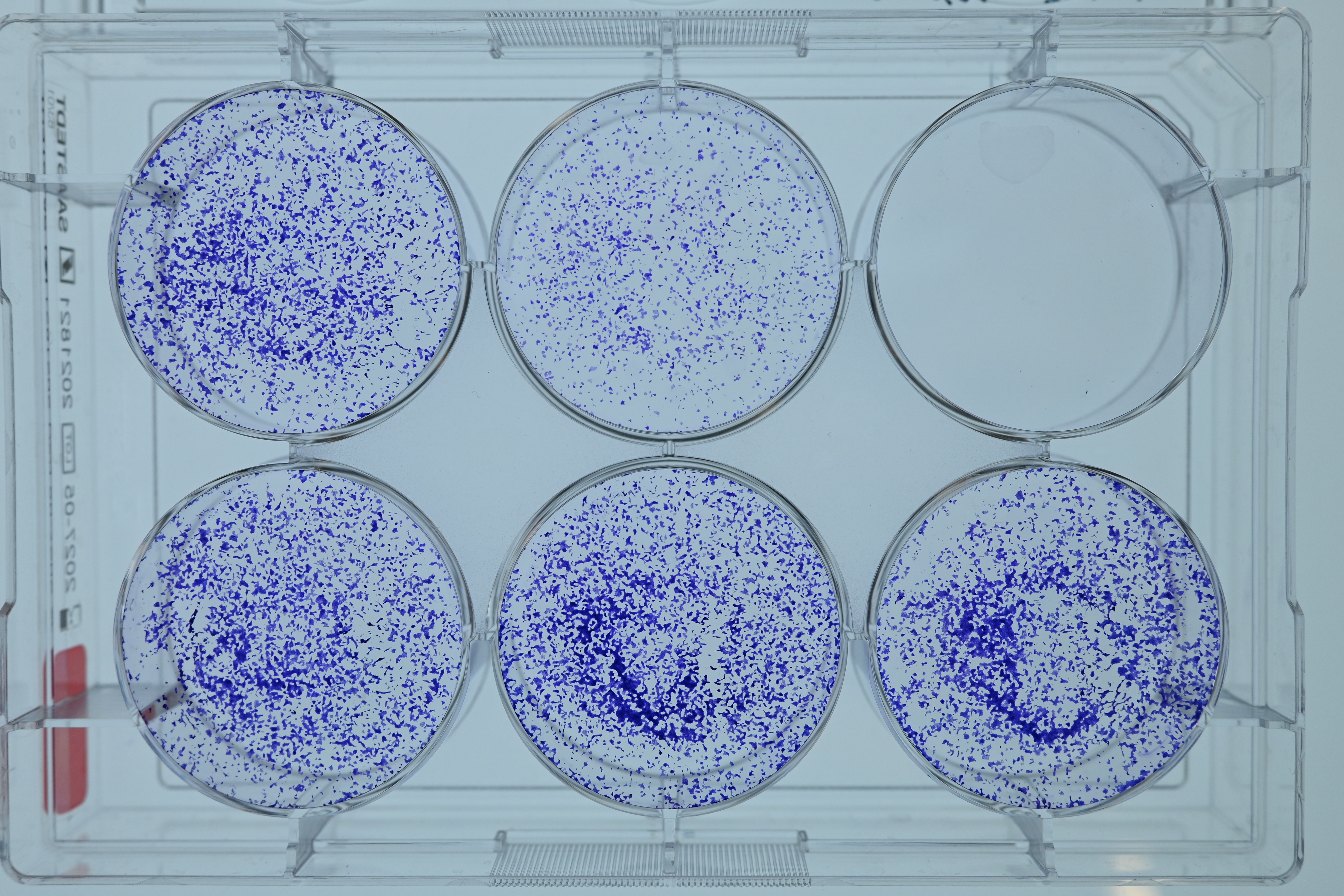

Supplement: Supplementary file 6 — Source Data for Figure 2 [file EMMM-15-e18014-s009.zip › Figure 2/Fig 2I/Fig 2 I.JPG]

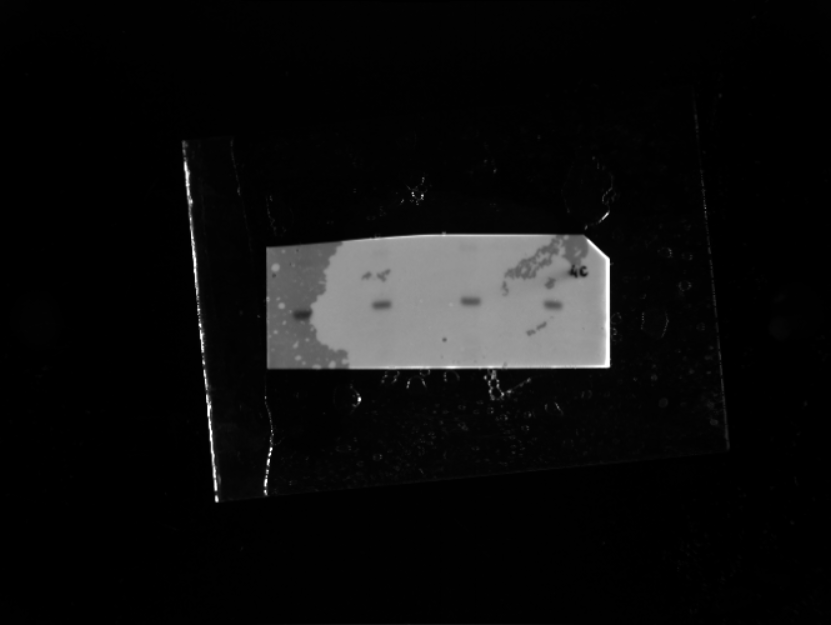

Supplement: Supplementary file 6 — Source Data for Figure 2 [file EMMM-15-e18014-s009.zip › Figure 2/Fig 2J/Fig 2J_gpx4-pic2_pub-Marker.tif]

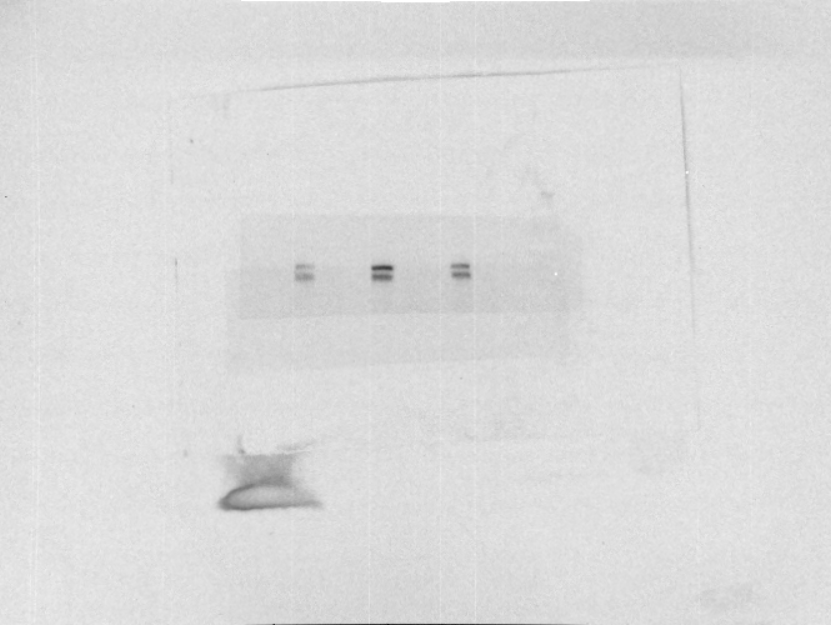

Supplement: Supplementary file 6 — Source Data for Figure 2 [file EMMM-15-e18014-s009.zip › Figure 2/Fig 2J/Fig 2J_lrp8-pic2_pub.tif]

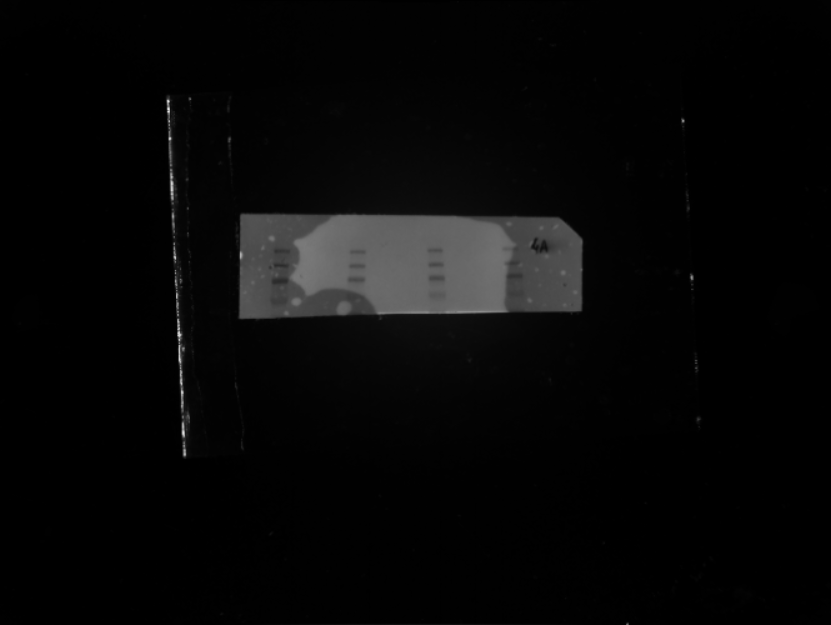

Supplement: Supplementary file 6 — Source Data for Figure 2 [file EMMM-15-e18014-s009.zip › Figure 2/Fig 2J/Fig 2J_lrp8-pic2_pub-Marker.tif]

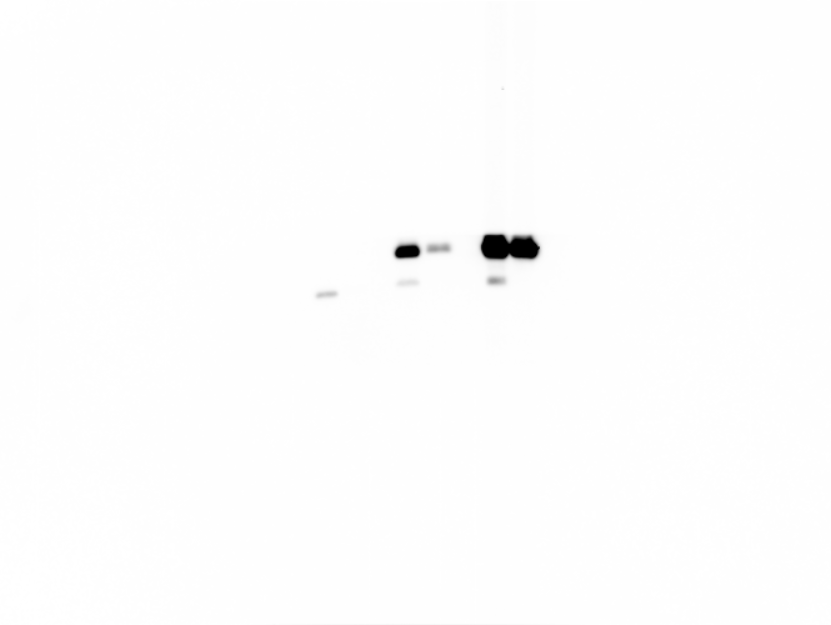

Supplement: Supplementary file 6 — Source Data for Figure 2 [file EMMM-15-e18014-s009.zip › Figure 2/Fig 2J/Fig 2J_gpx4-pic2_pub.tif]

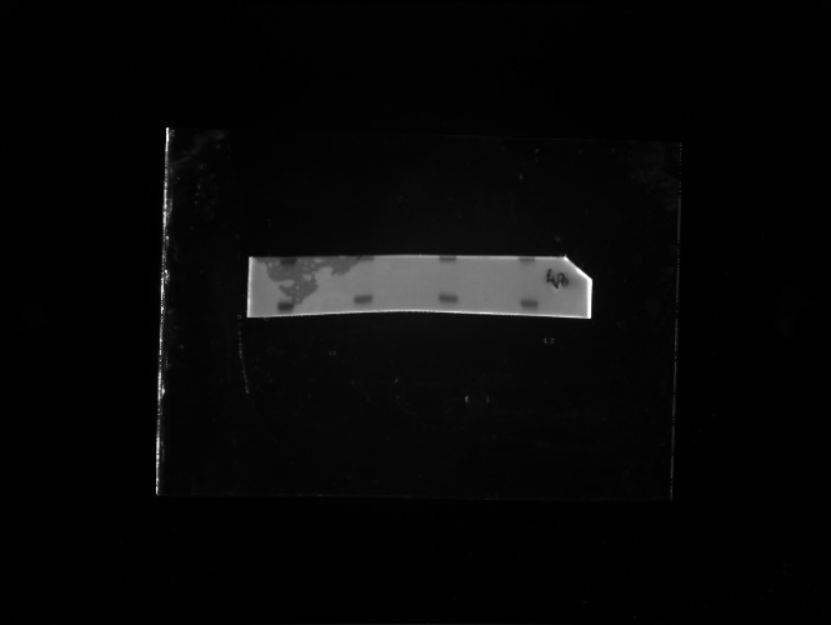

Supplement: Supplementary file 6 — Source Data for Figure 2 [file EMMM-15-e18014-s009.zip › Figure 2/Fig 2J/Fig 2J_actin-pic1_pub-Marker.tif]

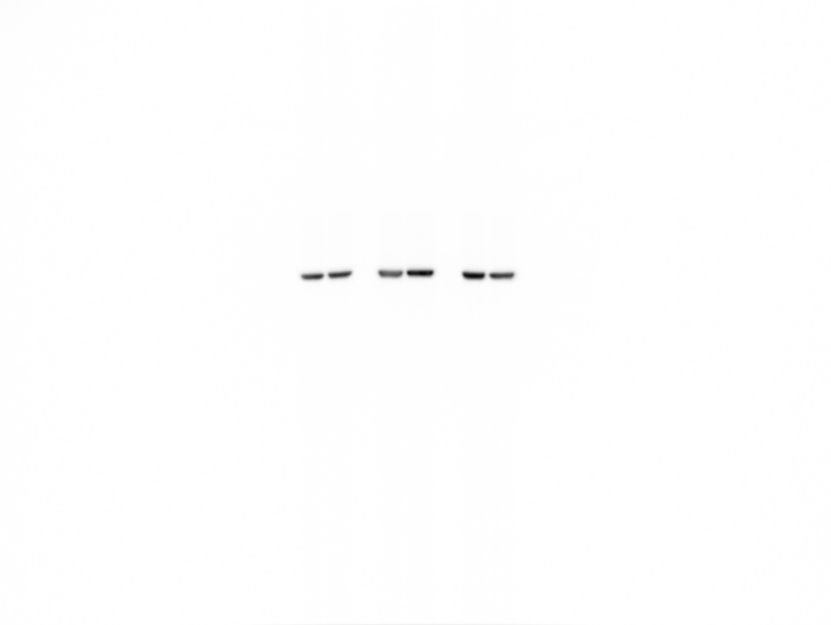

Supplement: Supplementary file 6 — Source Data for Figure 2 [file EMMM-15-e18014-s009.zip › Figure 2/Fig 2J/Fig 2J_actin-pic1_pub.tif]

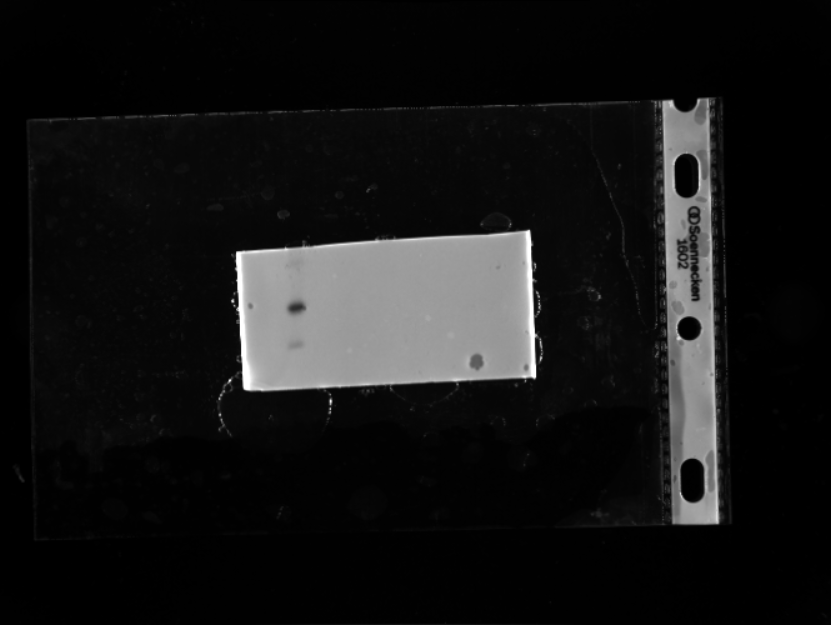

Supplement: Supplementary file 6 — Source Data for Figure 2 [file EMMM-15-e18014-s009.zip › Figure 2/Fig 2F/Western blot/Fig 2F-gpx4-marker_pub.tif]

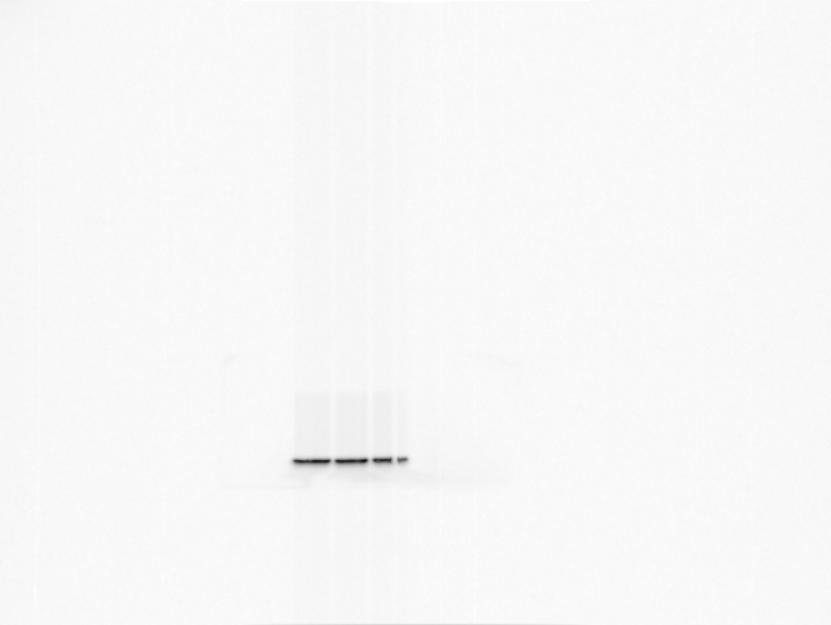

Supplement: Supplementary file 6 — Source Data for Figure 2 [file EMMM-15-e18014-s009.zip › Figure 2/Fig 2F/Western blot/Fig 2F-actin_pub.tif]

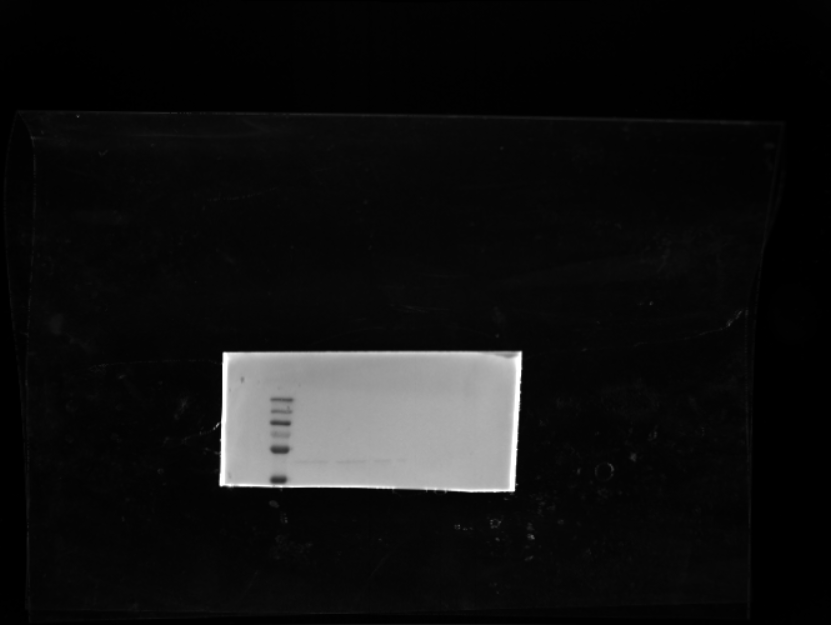

Supplement: Supplementary file 6 — Source Data for Figure 2 [file EMMM-15-e18014-s009.zip › Figure 2/Fig 2F/Western blot/Fig 2F-actin-marker_pub.tif]

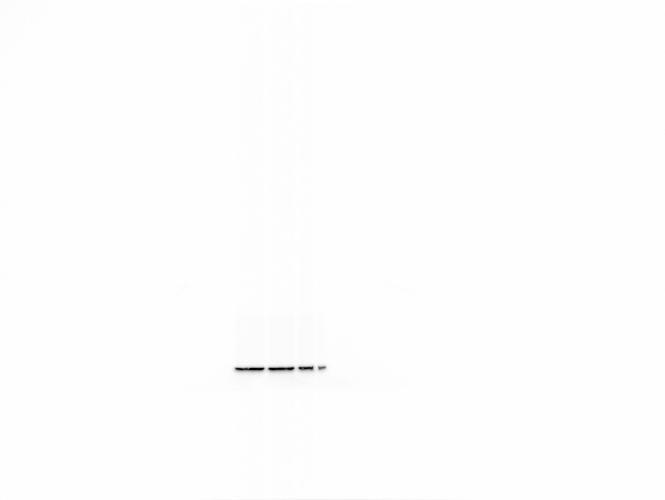

Supplement: Supplementary file 6 — Source Data for Figure 2 [file EMMM-15-e18014-s009.zip › Figure 2/Fig 2F/Western blot/Fig 2F-actin.tif]

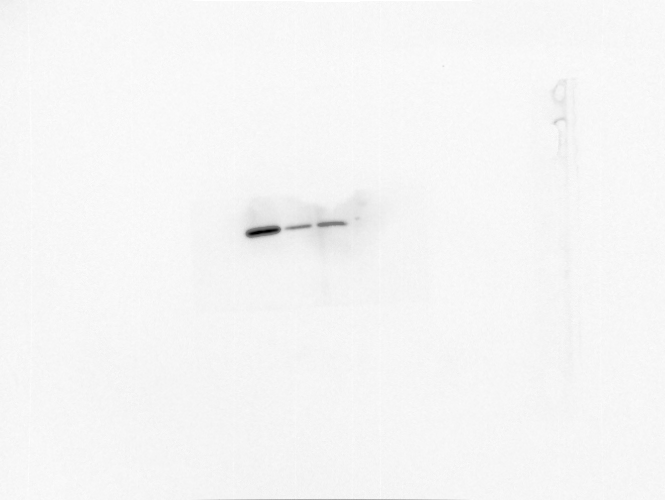

Supplement: Supplementary file 6 — Source Data for Figure 2 [file EMMM-15-e18014-s009.zip › Figure 2/Fig 2F/Western blot/Fig 2F-gpx4.tif]

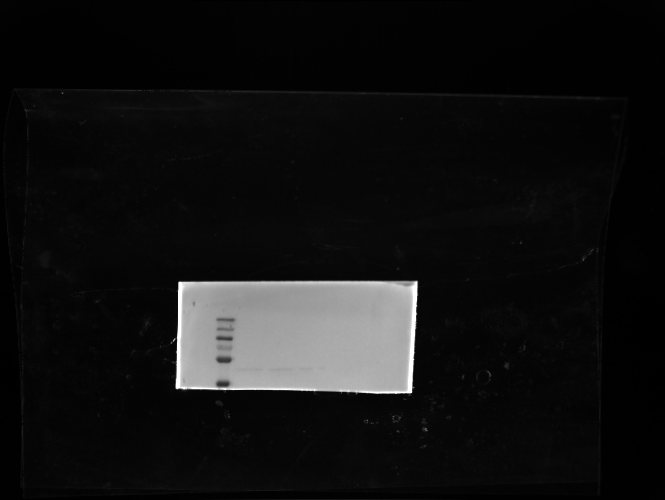

Supplement: Supplementary file 6 — Source Data for Figure 2 [file EMMM-15-e18014-s009.zip › Figure 2/Fig 2F/Western blot/Fig 2F-actin-marker.tif]

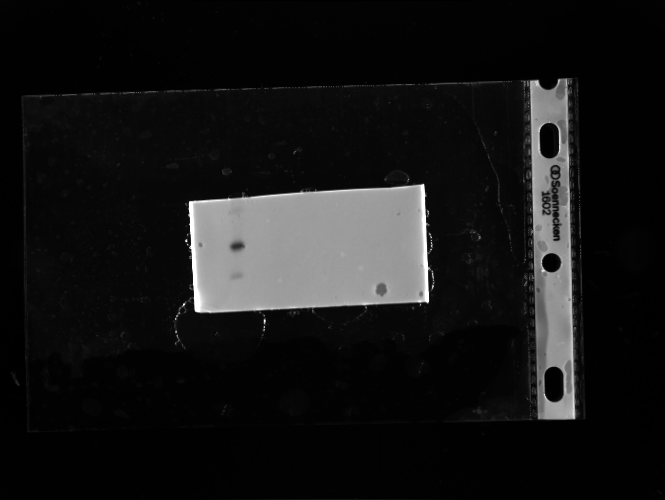

Supplement: Supplementary file 6 — Source Data for Figure 2 [file EMMM-15-e18014-s009.zip › Figure 2/Fig 2F/Western blot/Fig 2F-gpx4-marker.tif]

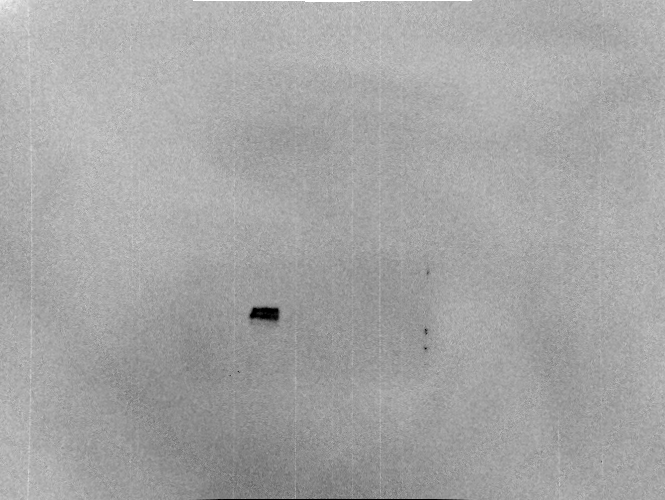

Supplement: Supplementary file 6 — Source Data for Figure 2 [file EMMM-15-e18014-s009.zip › Figure 2/Fig 2F/Western blot/Fig 2F-lrp8.tif]

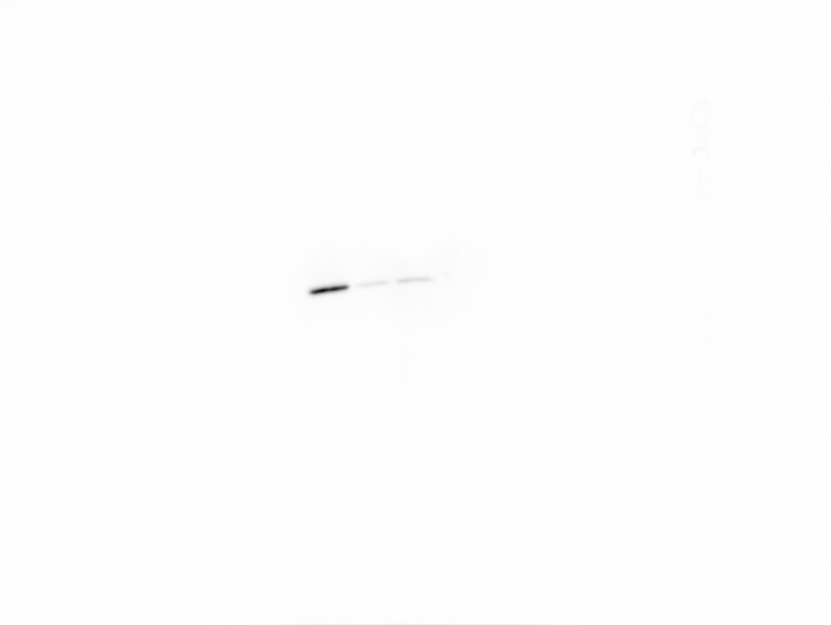

Supplement: Supplementary file 6 — Source Data for Figure 2 [file EMMM-15-e18014-s009.zip › Figure 2/Fig 2F/Western blot/Fig 2F-gpx4_pub.tif]

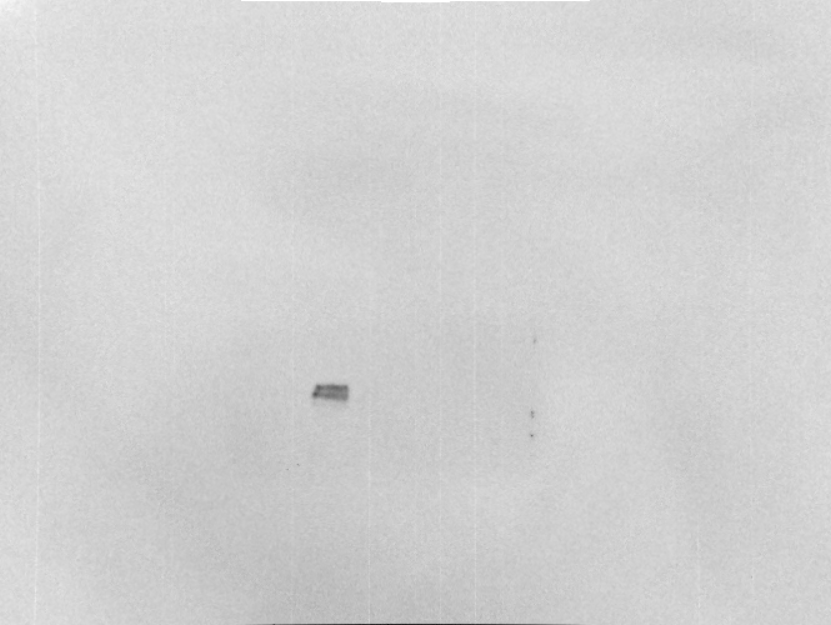

Supplement: Supplementary file 6 — Source Data for Figure 2 [file EMMM-15-e18014-s009.zip › Figure 2/Fig 2F/Western blot/Fig 2F-lrp8_pub.tif]

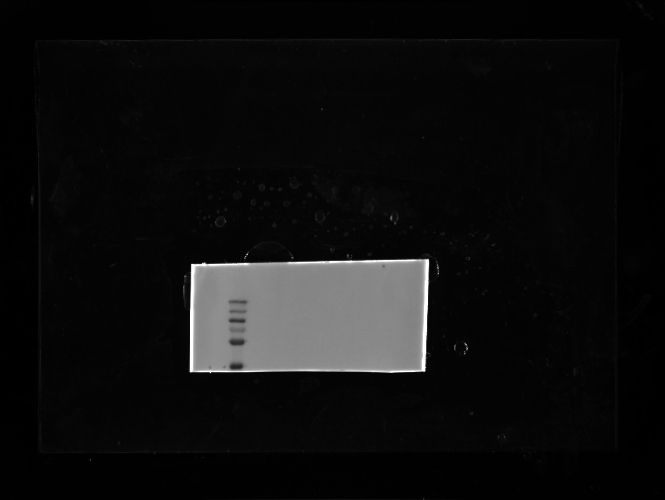

Supplement: Supplementary file 6 — Source Data for Figure 2 [file EMMM-15-e18014-s009.zip › Figure 2/Fig 2F/Western blot/Fig 2F-lrp8.markr.tif]

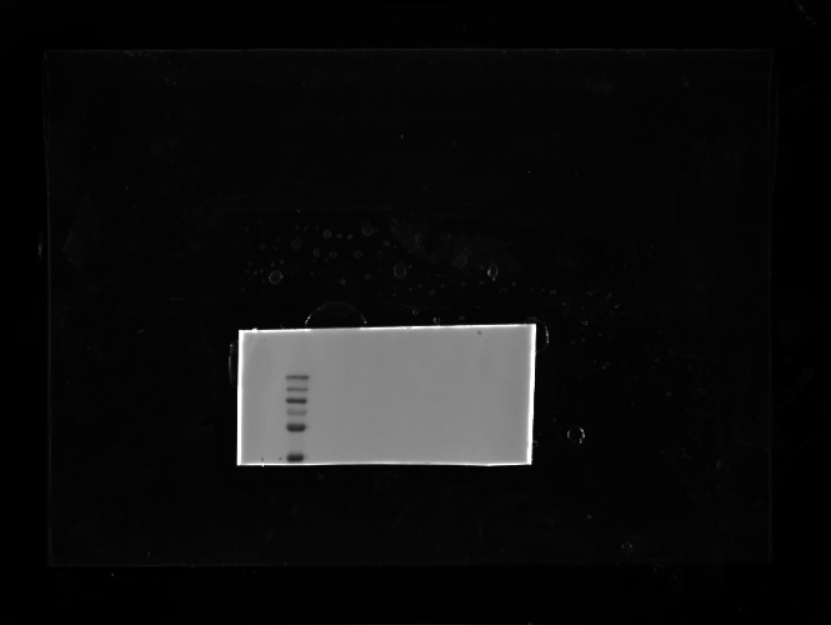

Supplement: Supplementary file 6 — Source Data for Figure 2 [file EMMM-15-e18014-s009.zip › Figure 2/Fig 2F/Western blot/Fig 2F-lrp8.markr_pub.tif]

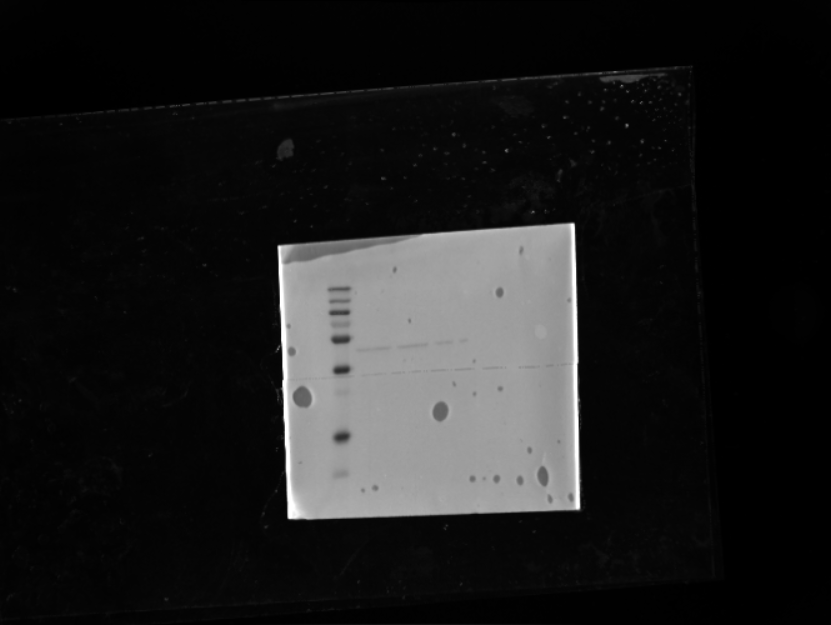

Supplement: Supplementary file 6 — Source Data for Figure 2 [file EMMM-15-e18014-s009.zip › Figure 2/Fig 2F/Western blot/Fig 2F-allmem_pub.tif]

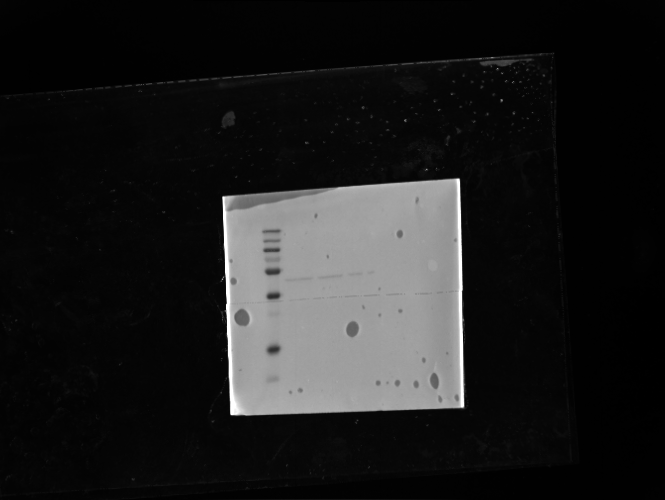

Supplement: Supplementary file 6 — Source Data for Figure 2 [file EMMM-15-e18014-s009.zip › Figure 2/Fig 2F/Western blot/Fig 2F-allmem.tif]

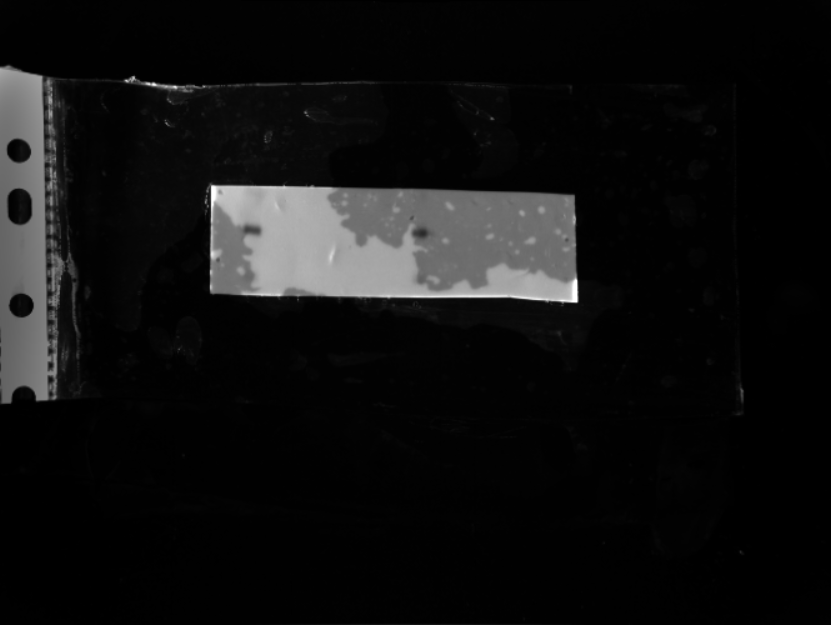

Supplement: Supplementary file 6 — Source Data for Figure 2 [file EMMM-15-e18014-s009.zip › Figure 2/Fig 2G/Western blot/Fig 2G-GPx4-marker_pub.tif]

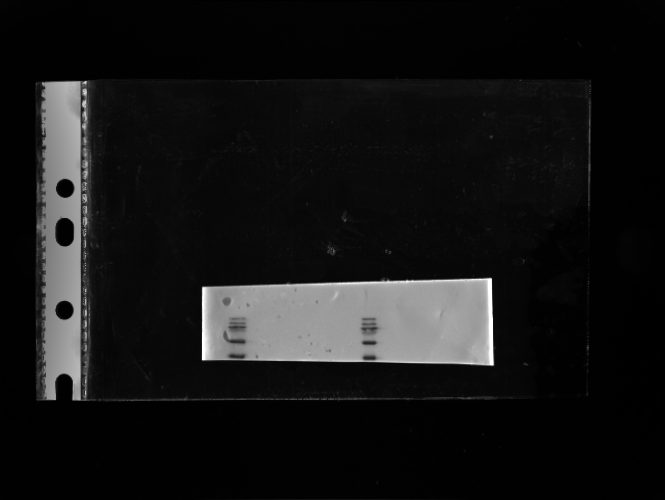

Supplement: Supplementary file 6 — Source Data for Figure 2 [file EMMM-15-e18014-s009.zip › Figure 2/Fig 2G/Western blot/Fig 2G-actin-marker.tif]

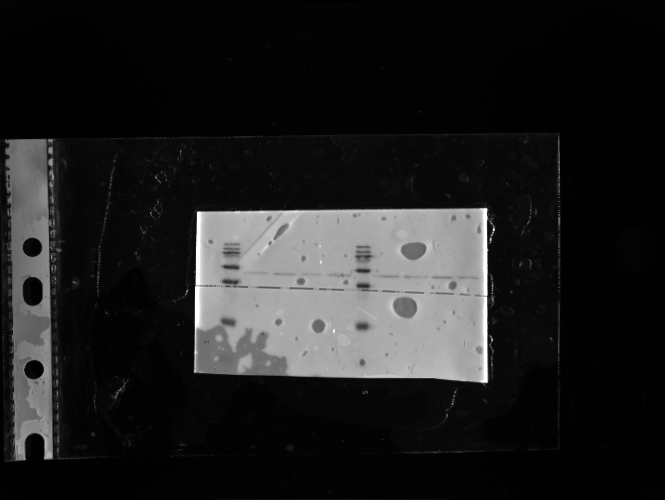

Supplement: Supplementary file 6 — Source Data for Figure 2 [file EMMM-15-e18014-s009.zip › Figure 2/Fig 2G/Western blot/Fig 2G-all mem.tif]

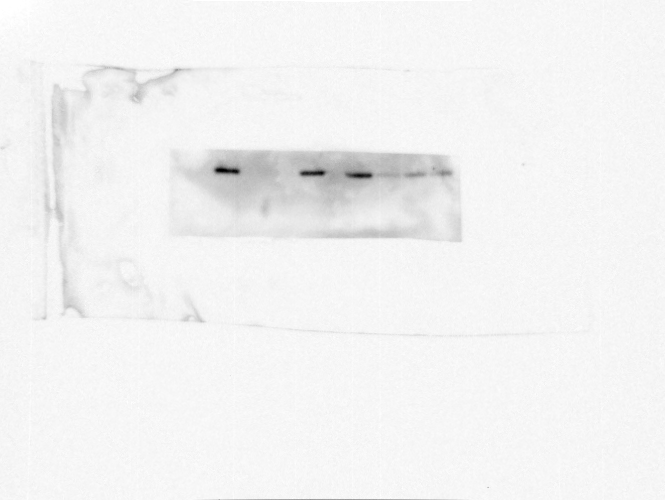

Supplement: Supplementary file 6 — Source Data for Figure 2 [file EMMM-15-e18014-s009.zip › Figure 2/Fig 2G/Western blot/Fig 2G-GPx4.tif]

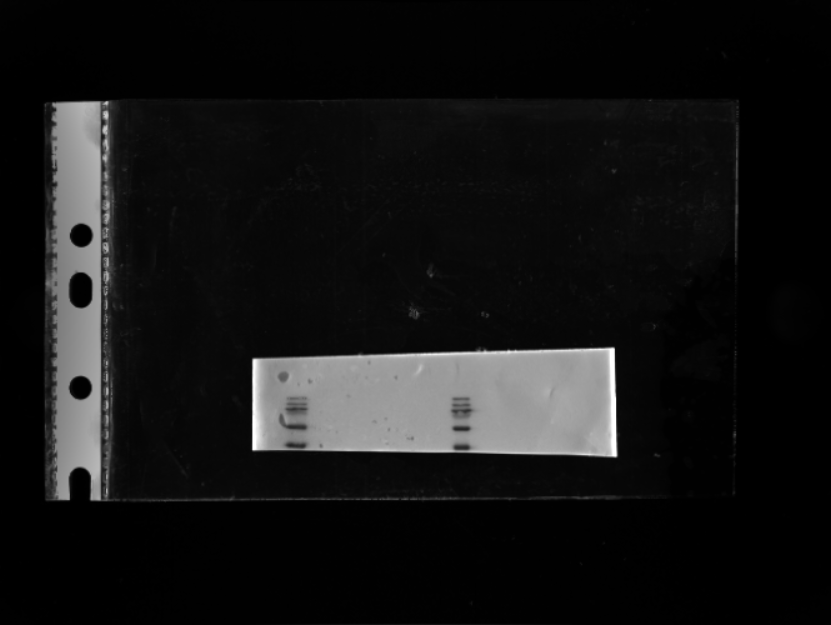

Supplement: Supplementary file 6 — Source Data for Figure 2 [file EMMM-15-e18014-s009.zip › Figure 2/Fig 2G/Western blot/Fig 2G-actin-marker_pub.tif]

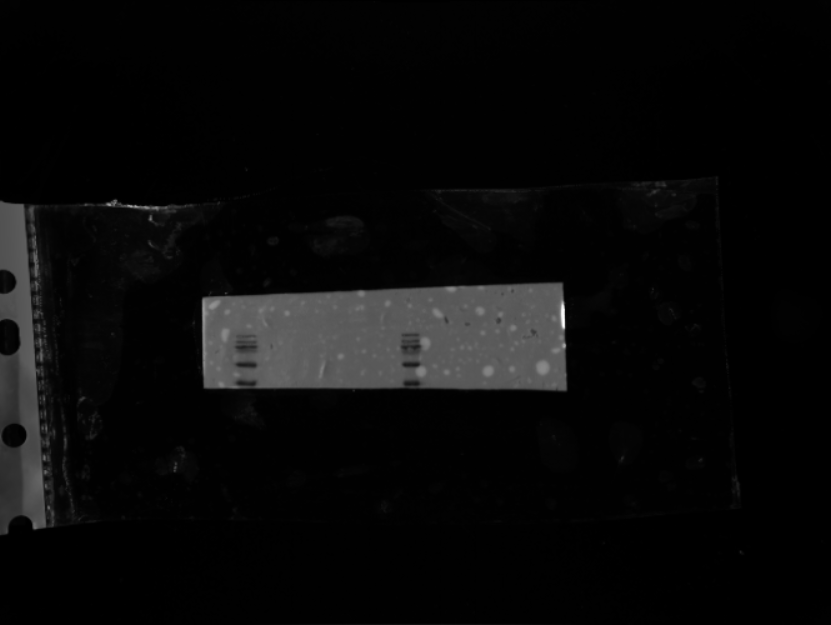

Supplement: Supplementary file 6 — Source Data for Figure 2 [file EMMM-15-e18014-s009.zip › Figure 2/Fig 2G/Western blot/Fig 2G-LRP8-MARKER_pub.tif]

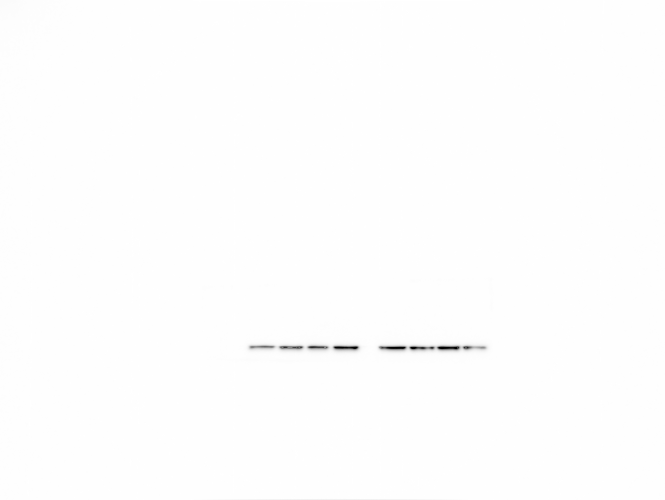

Supplement: Supplementary file 6 — Source Data for Figure 2 [file EMMM-15-e18014-s009.zip › Figure 2/Fig 2G/Western blot/Fig 2G-actin.tif]

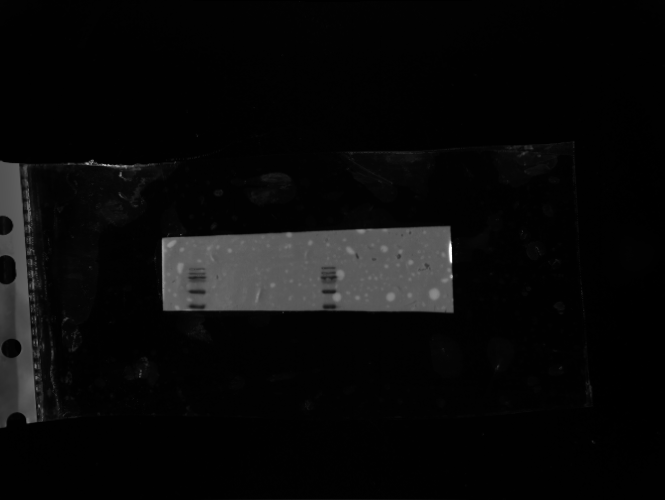

Supplement: Supplementary file 6 — Source Data for Figure 2 [file EMMM-15-e18014-s009.zip › Figure 2/Fig 2G/Western blot/Fig 2G-LRP8-MARKER.tif]

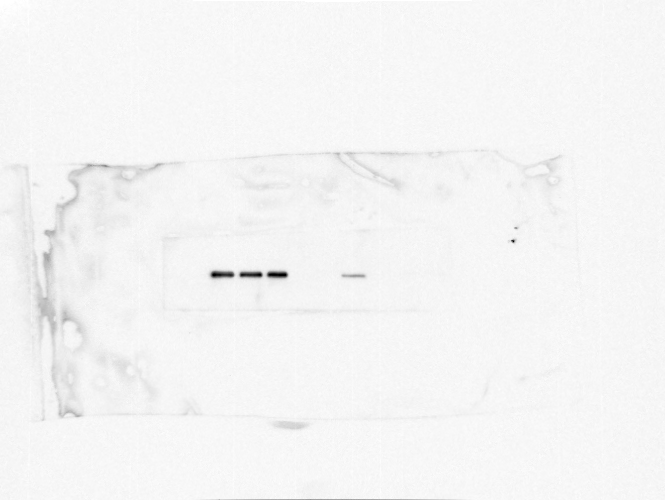

Supplement: Supplementary file 6 — Source Data for Figure 2 [file EMMM-15-e18014-s009.zip › Figure 2/Fig 2G/Western blot/Fig 2G-LRP8.tif]

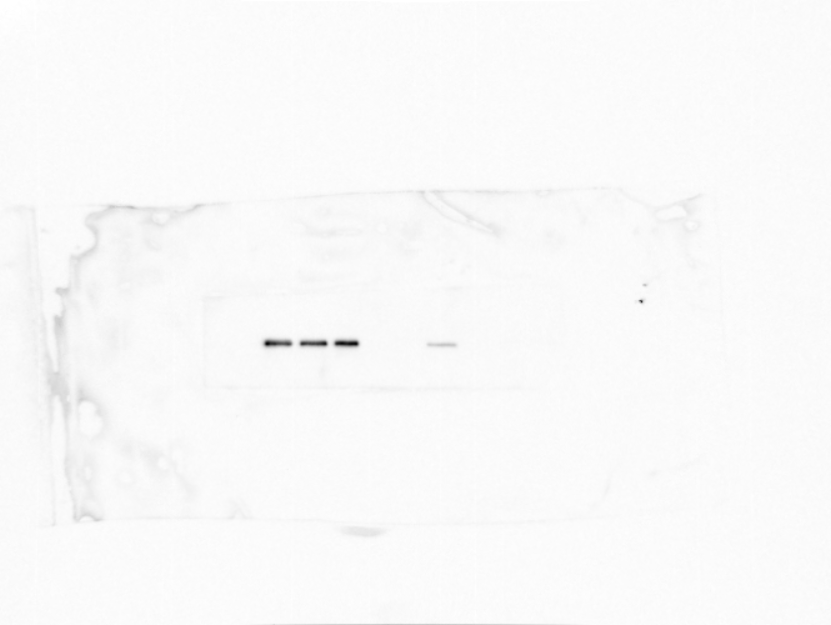

Supplement: Supplementary file 6 — Source Data for Figure 2 [file EMMM-15-e18014-s009.zip › Figure 2/Fig 2G/Western blot/Fig 2G-LRP8_pub.tif]

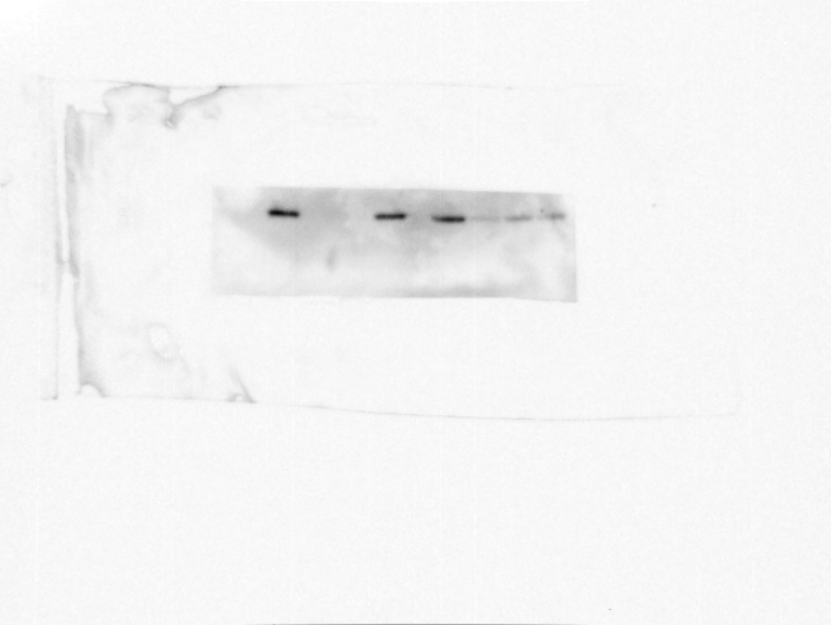

Supplement: Supplementary file 6 — Source Data for Figure 2 [file EMMM-15-e18014-s009.zip › Figure 2/Fig 2G/Western blot/Fig 2G-GPx4_pub.tif]

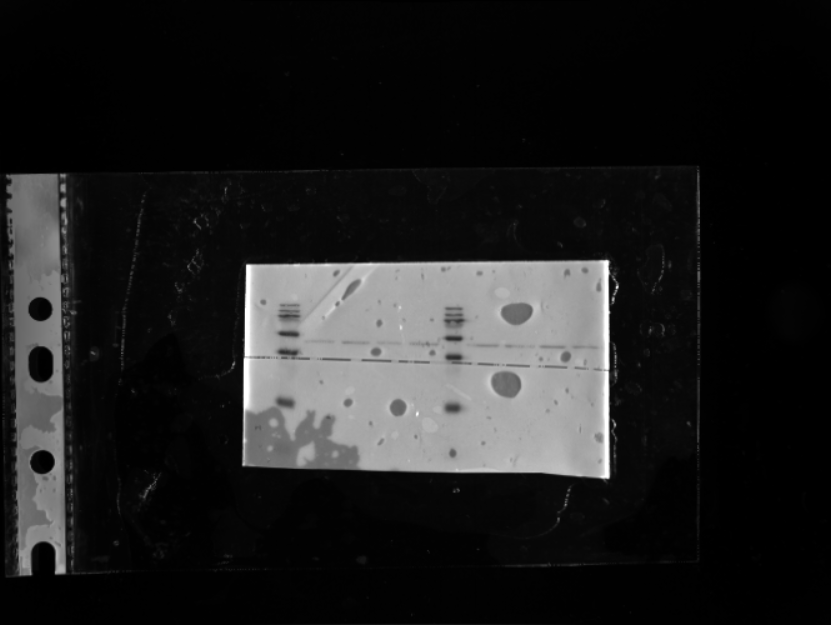

Supplement: Supplementary file 6 — Source Data for Figure 2 [file EMMM-15-e18014-s009.zip › Figure 2/Fig 2G/Western blot/Fig 2G-all mem_pub.tif]

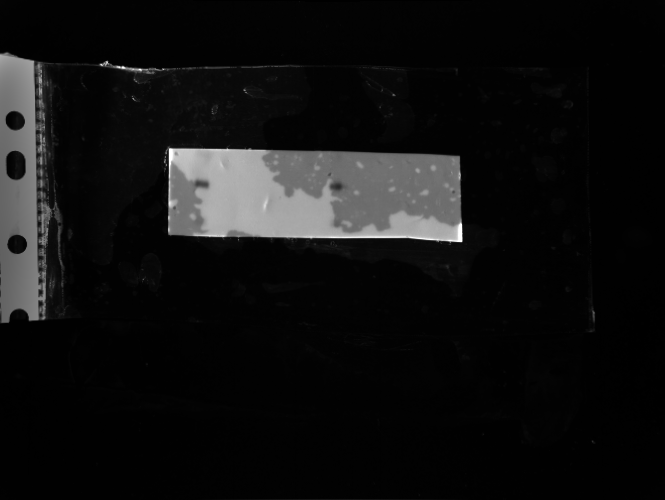

Supplement: Supplementary file 6 — Source Data for Figure 2 [file EMMM-15-e18014-s009.zip › Figure 2/Fig 2G/Western blot/Fig 2G-GPx4-marker.tif]

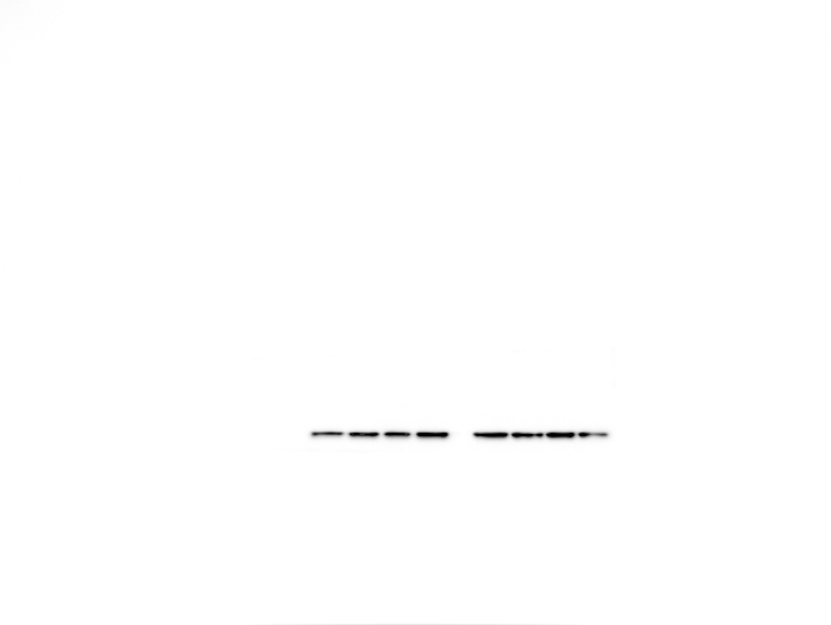

Supplement: Supplementary file 6 — Source Data for Figure 2 [file EMMM-15-e18014-s009.zip › Figure 2/Fig 2G/Western blot/Fig 2G-actin_pub.tif]

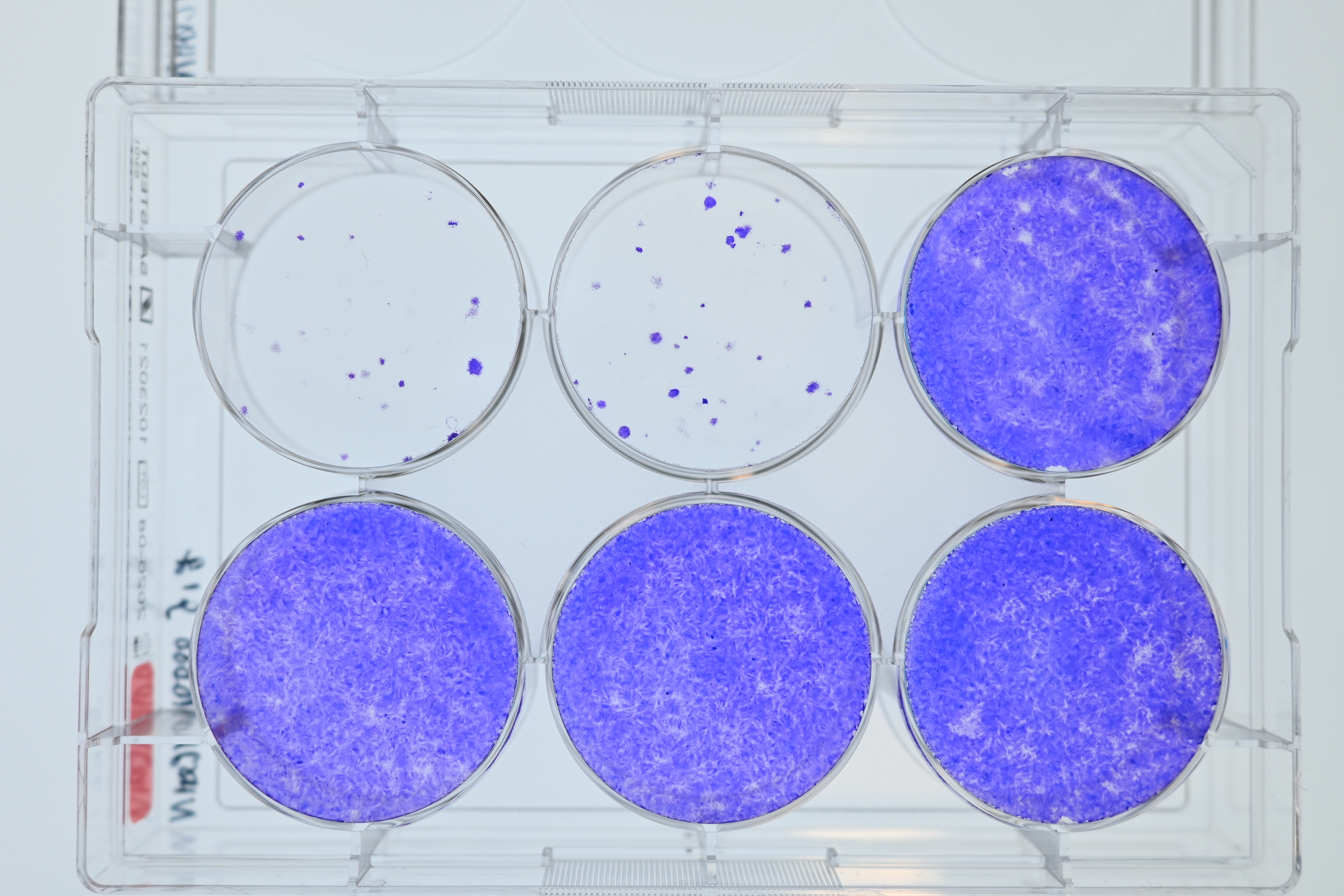

Supplement: Supplementary file 6 — Source Data for Figure 2 [file EMMM-15-e18014-s009.zip › Figure 2/Fig 2G/Clonogenic assay/Fig 2G-1.JPG]

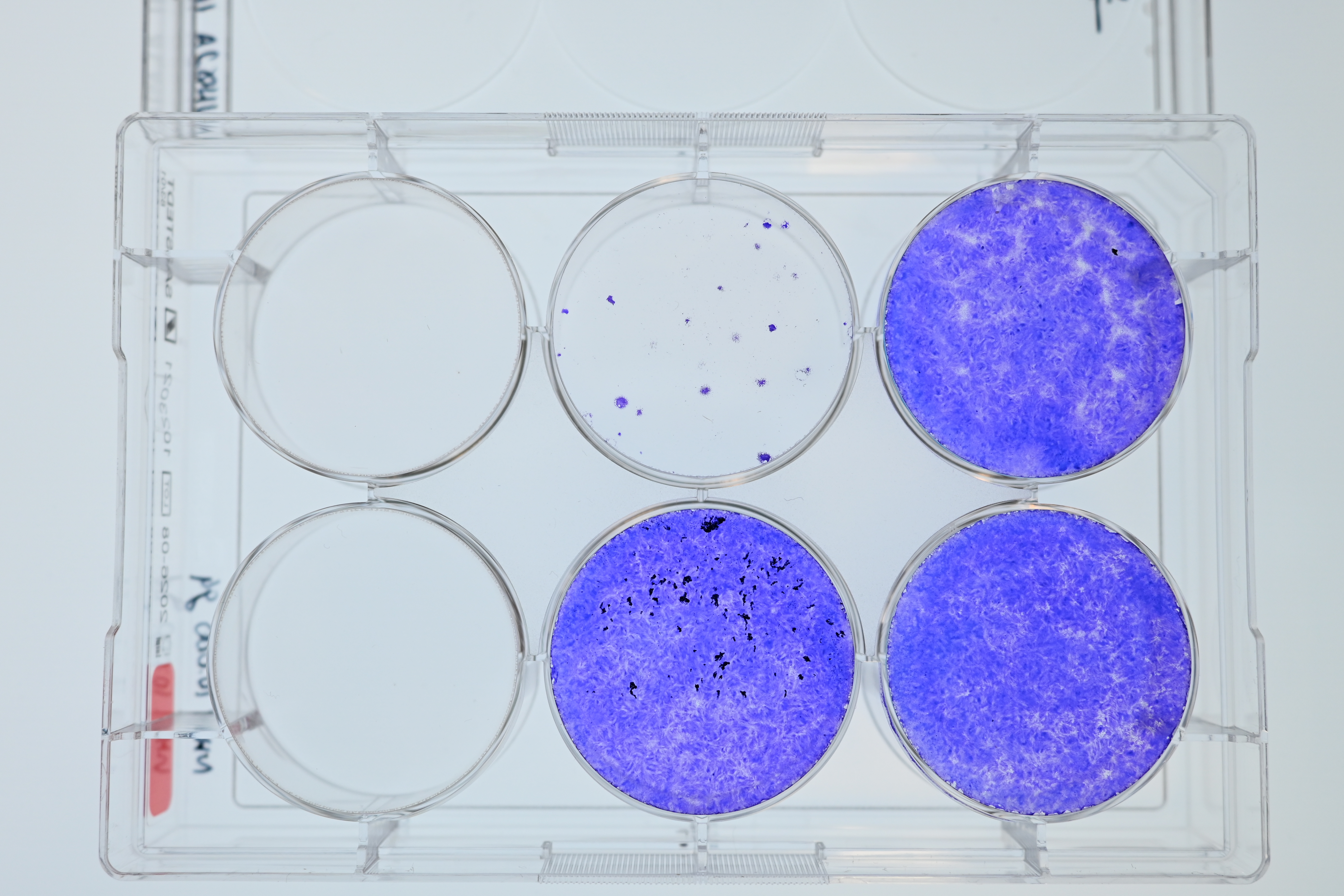

Supplement: Supplementary file 6 — Source Data for Figure 2 [file EMMM-15-e18014-s009.zip › Figure 2/Fig 2G/Clonogenic assay/Fig 2G-2.JPG]

## NH02A

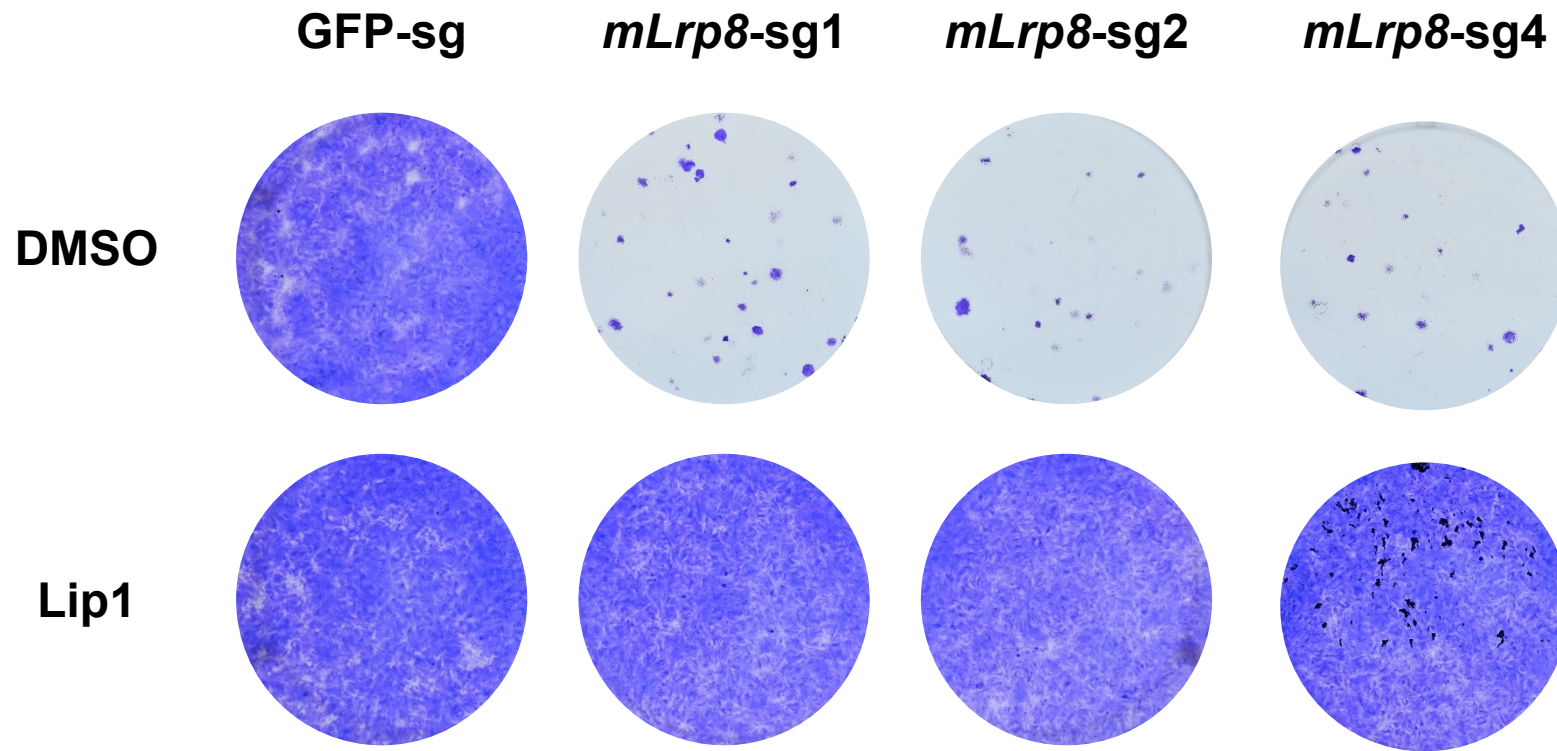

## NH02A

---

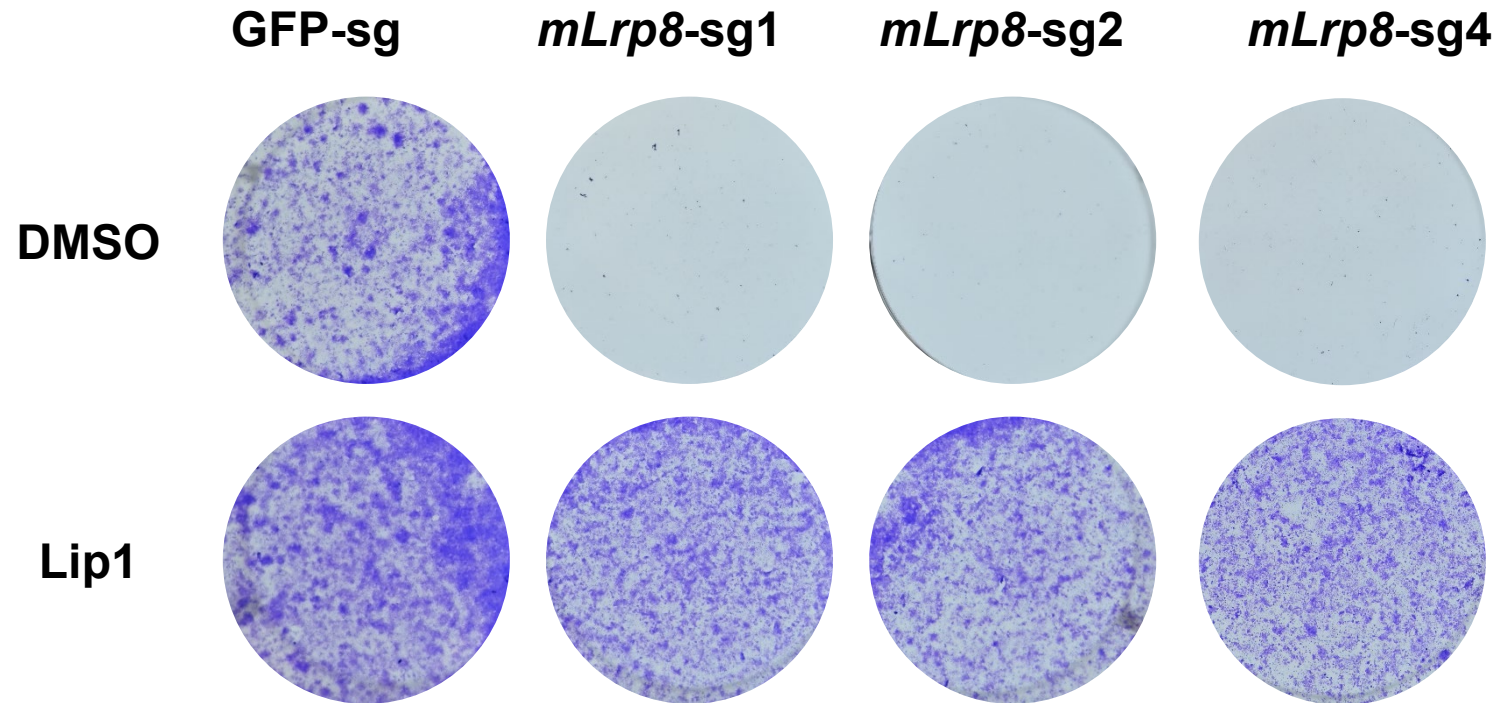

Supplement: Supplementary file 6 — Source Data for Figure 2 [file EMMM-15-e18014-s009.zip › Figure 2/Fig 2G/Clonogenic assay/Fig 2G.pdf]
