# Supplementary material for: LRP8‐mediated selenocysteine uptake is a targetable vulnerability in MYCN‐amplified neuroblastoma
Source: EMBO Mol Med. 2023 Jul 12;15(8):e18014. doi: 10.15252/emmm.202318014 (PMC10405063; doi:10.15252/emmm.202318014)
Supplement: Supplementary file 9 [file EMMM-15-e18014-s003.pdf]

EMBO Molecular Medicine

LRP8-mediated selenocysteine uptake is a targetable vulnerability in MYCN-amplified neuroblastoma.

José Friedmann Angeli, Hamed Alborzinia, Marcus Conrad, Bernhard Michalke, Christoph Bartenhagen, Werner Schmitz, Umut Yildiz, Elisa Espinet, Andreas Trumpp, Martin Eilers, Matthias Fischer, Andres Florez, Nesrine Aroua, Giulio Supert-Furga, Gabriele Büchel, zhiyi chen, florencio freitas, Felix Vogel, Julianna Varga, Jasmin batani, Svenja Meierjohann, Enrico Girardi, Ancely dos Santos, Thamara Xavier da Silva, Adriana przybylla, Petra Zeisberger, Almut Schulze, Ulrich Schweizer, Jiashuo Zheng, Tasneem Cheytan, Julie Haenlin, Marietta Fabiano, and Lisa Schlicker
DOI: 10.15252/emmm.202318014

Corresponding authors: José Friedmann Angeli (pedro.angeli@virchow.uni-wuerzburg.de) , Andreas Trumpp (a.trumpp@dkfz.de), José Friedmann Angeli (pedro.angeli@virchow.uni-wuerzburg.de), Hamed Alborzinia (hamed.alborzy@gmail.com)

Review Timeline:

Submission Date:	16th May 23
Editorial Decision:	25th May 23
Revision Received:	16th Jun 23
Editorial Decision:	19th Jun 23
Revision Received:	23rd Jun 23
Accepted:	23rd Jun 23

Editor: Lise Roth

Transaction Report:

(Note: Please note that the manuscript was previously reviewed at another journal and the reports were taken into account in the decision making process at EMBO Molecular Medicine. Since the original reviews are not subject to EMBO's transparent review process policy, the reports and author response cannot be published. With the exception of the correction of typographical or spelling errors that could be a source of ambiguity, letters and reports are not edited. Depending on transfer agreements, referee reports obtained elsewhere may or may not be included in this compilation. Referee reports are anonymous unless the Referee chooses to sign their reports.)

25th May 2023

Dear Pedro,

Thank you for the submission of your manuscript to EMBO Molecular Medicine. I have sent it to a single reviewer for evaluation, together with the existing referees' reports and your point-by-point rebuttal letter. We have now received the enclosed referee report. As you will see, the reviewer is very positive and supportive of publication, and I will therefore be able to accept your manuscript once the following editorial points will be addressed:

1/ Main manuscript text:
- Please provide a .docx formatted version of the manuscript text (including legends for the main figures, EV figures (if applicable) and tables). Only keep in track changes mode any new modification.
- Please address the queries from our data editors in track changes mode in the "data edited manuscript file". This file will be sent to you in a couple of days.
- Please provide up to 5 keywords.
- We note that you currently have together with you a total of 3 co-corresponding authors. Is that correct? Do you confirm equal contribution of these 3 people, able to take full responsibility for the paper and its content? Please also confirm that there are 3 first authors with equal contribution.
- Materials and methods:
o Please place this section after the discussion and before the figure legends.
o Cell cultures: Please provide information on origin and culture conditions for all cells used in the study.
o The table with oligonucleotides should be moved after the figure legends and named Table 1.
- Thank you for providing a 'Data Availability' section. Please remove "will be publicly accessible upon publication of this manuscript. Remaining data are available in the article, supplementary information or from the corresponding authors upon request."
- Acknowledgements: please provide information for funding sources in the submission system (this information should be identical in the submission system and in the manuscript).
- Author contributions: CRediT has replaced the traditional author contributions section because it offers a systematic machine-readable author contributions format that allows for more effective research assessment. Please remove the Authors Contributions from the manuscript and use the free text boxes beneath each contributing author's name in our system to add specific details on the author's contribution. More information is available in our guide to authors.
- Please rename competing interests: "Disclosure statement and competing interests". (We updated our journal's competing interests policy in January 2022 and request authors to consider both actual and perceived competing interests. Please review the policy https://www.embopress.org/competing-interests and update your competing interests if necessary.)
- References: please list the references in alphabetical order, with 10 authors listed before et al.

2/ Figures:
- The main figures need to be uploaded as individual, high resolution figure files. The legends should be in the manuscript text, after the References.
- Please provide exact p values, not a range, in the figures or in their legends, including for supplementary figures.
- The Appendix Figures should be compiled in one appendix PDF, with a Table of Content. The legends should be in the appendix file, underneath the corresponding figures. Please note that you have the possibility to make your appendix figures "Expanded View (EV) Figures" that are collapsible/expandable online. A maximum of 5 EV Figures can be typeset. EV Figures should be cited as 'Figure EV1, Figure EV2" etc... in the text and their respective legends should be included in the main text after the legends of regular figures. See detailed instructions here:
https://www.embopress.org/page/journal/17574684/authorguide#expandedview.
- Figure references in the manuscript text ('callouts') are missing for Fig 3E and Fig 4C. There are callouts for Supplementary Tables 1-3 but these are not provided. Please upload with revision and rename "Table EV1" etc.

3/ At EMBO Press we ask authors to provide source data for the main figures. Our source data coordinator will contact you to discuss which figure panels we would need source data for and will also provide you with helpful tips on how to upload and organize the files.

4/ Checklist: please clarify "please check with Matthias Fischer"

5/ Please provide The Paper Explained section: EMBO Molecular Medicine articles are accompanied by a summary of the articles to emphasize the major findings in the paper and their medical implications for the non-specialist reader. Please provide a draft summary of your article highlighting
- the medical issue you are addressing,
- the results obtained and
- their clinical impact.

This may be edited to ensure that readers understand the significance and context of the research. Please refer to any of our published articles for an example.

6/ Every published paper now includes a 'Synopsis' to further enhance discoverability. Synopses are displayed on the journal webpage and are freely accessible to all readers. They include a short stand first (maximum of 300 characters, including space) as well as 2-5 one-sentences bullet points that summarizes the paper. Please write the bullet points to summarize the key NEW findings. They should be designed to be complementary to the abstract - i.e. not repeat the same text. We encourage inclusion of key acronyms and quantitative information (maximum of 30 words / bullet point). Please use the passive voice.
Please also suggest a striking image or visual abstract to illustrate your article as a PNG file 550 px wide x 300-600 px high.

7/ As part of the EMBO Publications transparent editorial process initiative (see our Editorial at http://embomolmed.embopress.org/content/2/9/329), EMBO Molecular Medicine will publish online a Review Process File (RPF) to accompany accepted manuscripts.
This file will be published in conjunction with your paper and will include the anonymous referee reports, your point-by-point response and all pertinent correspondence relating to the manuscript. Let us know whether you agree with the publication of the RPF and as here, if you want to remove or not any figures from it prior to publication.
Please note that the Authors checklist will be published at the end of the RPF.

EMBO Molecular Medicine has a "scooping protection" policy, whereby similar findings that are published by others during review or revision are not a criterion for rejection.

I look forward to receiving your revised manuscript.

With kind regards,

Lise

Lise Roth, PhD
Senior Editor
EMBO Molecular Medicine

*Additional important information regarding Figures

Each figure should be given in a separate file and should have the following resolution:
Graphs 800-1,200 DPI
Photos 400-800 DPI
Colour (only CMYK) 300-400 DPI"

Figures are not edited by the production team. All lettering should be the same size and style; figure panels should be indicated by capital letters (A, B, C etc). Gridlines are not allowed except for log plots. Figures should be numbered in the order of their appearance in the text with Arabic numerals. Each Figure must have a separate legend and a caption is needed for each panel.

*Additional important information regarding figures and illustrations can be found at https://bit.ly/EMBOPressFigurePreparationGuideline. See also figure legend preparation guidelines: https://www.embopress.org/page/journal/17574684/authorguide#figureformat

The system will prompt you to fill in your funding and payment information. This will allow Wiley to send you a quote for the article processing charge (APC) in case of acceptance. This quote takes into account any reduction or fee waivers that you may be eligible for. Authors do not need to pay any fees before their manuscript is accepted and transferred to our publisher.

***** Reviewer's comments *****

Referee #1 (Remarks for Author):

Ferroptosis is a regulated form of cell death characterized by the accumulation of oxidatively damaged phospholipids. Recent

research suggests that inducing ferroptosis could be beneficial in treating forms of cancer that are resistant to conventional therapies. For example, studies report that MYCN-amplified neuroblastomas are exceptionally sensitive to ferroptosis triggered by GPX4 inhibition. However, the precise mechanisms behind why MYCN-amplified neuroblastomas are particularly sensitive to ferroptosis remain poorly understood.

In the current manuscript, genome-wide CRISPRa screens reveal an exceptional requirement for LRP8 as a ferroptosis suppressor in MYCN-amplified neuroblastoma. Notably, while previous studies have found that LRP8 loss sensitizes cancer cells to ferroptosis induction, this research convincingly establishes that the loss of LRP8 alone is sufficient to trigger ferroptosis in MYCN-amplified neuroblastoma. A key strength of this study lies in the characterization of the underlying mechanism behind the LRP8 dependency. Their investigations demonstrate that the LRP8 dependency is due to the low expression of system Xc- and, consequently, the limited uptake of selenium. This pivotal insight not only reaffirms the significance of LRP8 but also sheds light on the critical role of selenium metabolism as a key therapeutic vulnerability in MYCN-amplified neuroblastoma.

The experimental design and execution presented in this manuscript are exemplary, reflecting rigorous methodology and thoughtful analysis. The authors effectively address previous reviewers' comments, further strengthening the overall quality of their work. Its implications for targeted therapeutic approaches in this devastating form of cancer make it an important addition to the scientific literature. Based on the substantial contribution of these findings to our understanding of ferroptosis regulation in MYCN-amplified neuroblastoma, I recommend immediate publication of this manuscript.

-James Olzmann

19th Jun 2023

Dear Pedro,

Thank you for the submission of your revised manuscript to EMBO Molecular Medicine. Before I can accept your manuscript, please address the following remaining editorial points:

1/ Main manuscript text:
- Please address the remaining queries from our data editors (Fig. 4F/Appendix Fig. S4: Please define the central band, boxes and whiskers of the boxplot)
- We can accommodate a maximum of 5 keywords, please adjust accordingly.
- We note that you currently have together with you a total of 3 co-corresponding authors. Is that correct? Do you confirm equal contribution of these 3 people, able to take full responsibility for the paper and its content? Please also confirm that there are 3 first authors with equal contribution.
- Materials and methods, Human samples: please indicate the details of authority granting ethics approval, provide reference number for approval. Please include a statement confirming that informed consent was obtained from all subjects and that the experiments conformed to the principles set out in the WMA Declaration of Helsinki and the Department of Health and Human Services Belmont Report. Please correct the checklist accordingly (it is currently stated "please check with Matthias Fischer").
- Acknowledgements: The funding information provided in the manuscript should be the same as the one provided in the submission system. Currently, SFB873, "RiskY-AML", the "Integrate-TN" Consortium funded by the Deutsche Krebshilfe, the Dietmar Hopp Foundation, Humboldt Postdoctoral Fellowship, FOR2314, SFB 1399, Förderverein für krebskranke Kinder e.V. Köln Austrian Academy of Sciences, a Marie Sklodowska-Curie fellowship (MSCA-IF-2014-661491) are missing from the submission system.

2/ Figures and Appendix:
- Thank you for providing exact p values. Please also indicate the exact p values for n.s, non significant p values.
- We note that you currently have for 4 figures and 6 Appendix figures. You have the possibility if you wish to make some of the Appendix figures "main figures". Alternatively, you could have up to 5 EV (Expanded View) figures, which are expandable/collapsible online, and which should be cited as "Figure EV1" etc. These figures should then be uploaded separately as figures and the corresponding legends would remain in the manuscript. If you wish to keep them all in the Appendix file, the legends should then be in the Appendix file and not in the manuscript. The Appendix table of content needs page numbers. Please contact us if you need further guidance.
- We found references to tables EV1-EV4 in the main manuscript text, but only found 1 table in the manuscript (Table EV4). Please clarify. If the current table EV4 remains the only table, please rename it Table 1 and should be moved after the figure legends.
- Your manuscript contains error bars based on n=2 (e.g. figure 3H, appendix figure S6C). Please use scatter blots showing the individual datapoints in these cases. The use of statistical tests needs to be justified.

3/ Source Data:
While I found the complete Source Data checklist, I could not find the Source Data themselves. Could you upload them again, 1 file per figure? If you have any question regarding the Source Data, please contact me or Hannah.

4/ Thank you for providing The Paper Explained. I included minor edits, please let me know if you agree or amend as you see fit, and incorporate the final text in the main manuscript file:
Problem
Ferroptosis, a distinct form of cell death, has emerged as a potential approach for eliminating solid tumors that are refractory to treatment. However, inhibiting the selenoprotein glutathione peroxidase 4 (GPX4), which suppresses ferroptosis, remains challenging due to the absence of suitable inhibitors and to the potential systemic toxicity. Our previous findings have demonstrated a notable dependence on GPX4 in high-risk MYCN-amplified neuroblastomas, yet the precise factors contributing to this phenomenon remain incompletely understood.
Results
Our study reveals that high-risk MYCN-amplified neuroblastomas exhibit a significant dependency on LRP8. Through genome-wide and single-cell transcriptomics CRISPR-activation screens, we identify the low-density lipoprotein receptor-related protein 8 (LRP8) as a critical factor in selenium/selenocysteine metabolism in MYCN-amplified cancers. We find that MYCN-amplified cells ineficiently activate alternative selenium/selenocysteine pathways, resulting in vulnerability to LRP8 inhibition. These metabolic vulnerabilities provide a unique opportunity to target LRP8 to induce ferroptosis selectively and safely.
Impact
In light of the limited success in repurposing adult oncology drugs for neuroblastoma treatment, our research presents a significant advance by introducing innovative strategies based on ferroptosis. By specifically focusing on targeting LRP8, we have identified a potential breakthrough that not only offers novel therapeutic approaches for neuroblastoma but also holds promise for a range of pediatric malignancies and MYCN-driven cancers.

5/ To fit our style and format, I slightly shortened your synopsis text, please let me know if you agree with the following:
"The low-density lipoprotein receptor (LRP8) was identified as a critical suppressor of ferroptosis in MYCN-amplified neuroblastoma. Blocking selenium/selenocysteine uptake mechanisms via LRP8 offers a selective strategy to induce ferroptosis and disrupt GPX4 function.
• Ferroptosis, a cell death modality, is gaining interest as a therapeutic approach against challenging tumors.
• GPX4 is crucial for suppressing ferroptosis, but suitable in vivo inhibitors are lacking, limiting translation to cancer therapies.
• Genome-wide and single-cell CRISPR-activation screens reveal LRP8 as a critical ferroptosis suppressor in MYCN-amplified neuroblastoma.
• Blocking selenium/selenocysteine uptake via LRP8 disrupts GPX4 function and selectively induces ferroptotic cell death.
• LRP8 dependency emerges as the result of the low system Xc- activity suggesting that targeting LRP8 could be explore in other entities such as AML and lymphoma."
Thank you for also providing a nice synopsis image. Please upload it as a png/tiff/jpeg file 550 px wide x 300-600 px high.

6/ As part of the EMBO Publications transparent editorial process initiative (see our Editorial at http://embomolmed.embopress.org/content/2/9/329), EMBO Molecular Medicine will publish online a Review Process File (RPF) to accompany accepted manuscripts.
This file will be published in conjunction with your paper and will include the referee report, your point-by-point response and all pertinent correspondence relating to the manuscript (the review that took place at the previous journal will not be included).
Let us know whether you agree with the publication of the RPF and as here, if you want to remove or not any figures from it prior to publication.
Please note that the Authors checklist will be published at the end of the RPF.

I look forward to receiving your revised manuscript as soon as possible,

With kind regards,

Lise

Lise Roth, PhD
Senior Editor
EMBO Molecular Medicine

The authors addressed the remaining minor editorial issues.

23rd Jun 2023

Dear Pedro,

Thank you for providing your revised files. I am pleased to inform you that your manuscript is now accepted for publication in EMBO Molecular Medicine pending one minor change:

Please remove the statistical test (p=) in your figures where n=2 (Figure 3H and Appendix Fig. S6). Once this is done, you may send the manuscript to me via email and I'll upload it in the system.

Please read below for additional IMPORTANT information regarding your article, its publication and the production process.

Congratulations on your interesting work!

With my best wishes,

Lise

Lise Roth, Ph.D
Senior Editor
EMBO Molecular Medicine

*** *** *** IMPORTANT INFORMATION *** *** ***

SPEED OF PUBLICATION
The journal aims for rapid publication of papers, using using the advance online publication "Early View" to expedite the process: A properly copy-edited and formatted version will be published as "Early View" after the proofs have been corrected. Please help the Editors and publisher avoid delays by providing e-mail address(es), telephone and fax numbers at which author(s) can be contacted.

Should you be planning a Press Release on your article, please get in contact with embomolmed@wiley.com as early as possible, in order to coordinate publication and release dates.

LICENSE AND PAYMENT:

All articles published in EMBO Molecular Medicine are fully open access: immediately and freely available to read, download and share.

EMBO Molecular Medicine charges an article processing charge (APC) to cover the publication costs. You, as the corresponding author for this manuscript, should have already received a quote with the article processing fee separately. Please let us know in case this quote has not been received.

Once your article is at Wiley for editorial production you will receive an email from Wiley's Author Services system, which will ask you to log in and will present you with the publication license form for completion. Within the same system the publication fee can be paid by credit card, an invoice, pro forma invoice or purchase order can be requested.

Payment of the publication charge and the signed Open Access Agreement form must be received before the article can be published online.

PROOFS

You will receive the proofs by e-mail approximately 2 weeks after all relevant files have been sent o our Production Office. Please return them within 48 hours and if there should be any problems, please contact the production office at embopressproduction@wiley.com.

Please inform us if there is likely to be any difficulty in reaching you at the above address at that time. Failure to meet our

deadlines may result in a delay of publication.

All further communications concerning your paper proofs should quote reference number EMM-2023-18014-V3 and be directed to the production office at embopressproduction@wiley.com.

EMBO Press Author Checklist

Corresponding Author Name: Dr. Hamed Alborzinia, Prof. Andreas Trumpp, Prof. José Pedro	
Journal Submitted to: EMBO Molecular Medicine	
Manuscript Number: EMM-2023-18014	

USEFUL LINKS FOR COMPLETING THIS FORM
The EMBO Journal - Author Guidelines
EMBO Reports - Author Guidelines
Molecular Systems Biology - Author Guidelines
EMBO Molecular Medicine - Author Guidelines

Reporting Checklist for Life Science Articles (updated January
This checklist is adapted from Materials Design Analysis Reporting (MDAR) Checklist for Authors. MDAR establishes a minimum set of requirements in transparent reporting in the life sciences (see Statement of Task: 10.31222/osf.io/9sm4x). Please follow the journal's guidelines in preparing your
Please note that a copy of this checklist will be published alongside your article.

Abridged guidelines for figures
1. Data
The data shown in figures should satisfy the following conditions:
- ➞ the data were obtained and processed according to the field's best practice and are presented to reflect the results of the experiments in an accurate and unbiased manner.
- ➞ ideally, figure panels should include only measurements that are directly comparable to each other and obtained with the same assay.
- ➞ plots include clearly labeled error bars for independent experiments and sample sizes. Unless justified, error bars should not be shown for technical
- ➞ if n<5, the individual data points from each experiment should be plotted. Any statistical test employed should be justified.
- ➞ Source Data should be included to report the data underlying figures according to the guidelines set out in the authorship guidelines on Data

2. Captions
Each figure caption should contain the following information, for each panel where they are relevant:
- ➞ a specification of the experimental system investigated (eg cell line, species name).
- ➞ the assay(s) and method(s) used to carry out the reported observations and measurements.
- ➞ an explicit mention of the biological and chemical entity(ies) that are being measured.
- ➞ an explicit mention of the biological and chemical entity(ies) that are altered/varied/perturbed in a controlled manner.
- ➞ the exact sample size (n) for each experimental group/condition, given as a number, not a range;
- ➞ a description of the sample collection allowing the reader to understand whether the samples represent technical or biological replicates (including how many animals, litters, cultures, etc.).
- ➞ a statement of how many times the experiment shown was independently replicated in the laboratory.
- ➞ definitions of statistical methods and measures:
  - common tests, such as t-test (please specify whether paired vs. unpaired), simple χ2 tests, Wilcoxon and Mann-Whitney tests, can be unambiguously identified by name only, but more complex techniques should be described in the methods section;
  - are tests one-sided or two-sided?
  - are there adjustments for multiple comparisons?
  - exact statistical test results, e.g., P values = x but not P values < x;
  - definition of 'center values' as median or average;
  - definition of error bars as s.d. or s.e.m.

Please complete ALL of the questions below.
Select "Not Applicable" only when the requested information is not relevant for your study.

Materials

**Newly Created Materials**	**Information included in the manuscript?**	**In which section is the information available?** (Reagents and Tools Table, Materials and Methods, Figures, Data Availability Section)
New materials and reagents need to be available; do any restrictions apply?	Yes	Methods - Molecular cloning: CROP-seq MS2 lentivector. Plasmid is avaialbe upon request

**Antibodies**	**Information included in the manuscript?**	**In which section is the information available?** (Reagents and Tools Table, Materials and Methods, Figures, Data Availability Section)
For **antibodies** provide the following information:<br>- Commercial antibodies: RRID (if possible) or supplier name, catalogue number and or/clone number<br>- Non-commercial: RRID or citation	Yes	Materials and Methods

**DNA and RNA sequences**	**Information included in the manuscript?**	**In which section is the information available?** (Reagents and Tools Table, Materials and Methods, Figures, Data Availability Section)
**Short novel DNA or RNA including primers, probes**: provide the sequences.	Yes	custom gRNA library targeting [illegible] hits from the primary screen. Guide RNA sequences of this focused libary can be found in Supplementary Table 2. Sequence information of individual gRNA-cloning are listed in the Methods - Molecular cloning

**Cell materials**	**Information included in the manuscript?**	**In which section is the information available?** (Reagents and Tools Table, Materials and Methods, Figures, Data Availability Section)
**Cell lines:** Provide species information, strain. Provide accession number in repository **OR** supplier name, catalog number, clone number, and/**OR** RRID.	Yes	Human neuroblastoma cell lines: SK-N-FI, SK-N-DZ, ... cells were purchased from ATCC.
**Primary cultures:** Provide species, strain, sex of origin, genetic modification status.	Not Applicable	
Report if the cell lines were recently **authenticated** (e.g., by STR profiling) and tested for mycoplasma contamination.	Yes	Materials and Methods - Cell culture

**Experimental animals**	**Information included in the manuscript?**	**In which section is the information available?** (Reagents and Tools Table, Materials and Methods, Figures, Data Availability Section)
**Laboratory animals or Model organisms:** Provide species, strain, sex, age, genetic modification status. Provide accession number in repository **OR** supplier name, catalog number, clone number, **OR** RRID.	Yes	Materials and Methods
**Animal observed in or captured from the field:** Provide species, sex, and age where possible**.**	Not Applicable	
Please detail **housing and husbandry conditions**.	Yes	Materials and Methods

**Plants and microbes**	**Information included in the manuscript?**	**In which section is the information available?** (Reagents and Tools Table, Materials and Methods, Figures, Data Availability Section)
**Plants:** provide species and strain, ecotype and cultivar where relevant, unique accession number if available, and source (including location for collected wild specimens).	Not Applicable	
**Microbes:** provide species and strain, unique accession number if available, and source.	Yes	Methods: Bacteria used for amplification of plasmid DNA

**Human research participants**	**Information included in the manuscript?**	**In which section is the information available?** (Reagents and Tools Table, Materials and Methods, Figures, Data Availability Section)
If collected and within the bounds of privacy constraints report on age, sex and gender or ethnicity for all study participants.	Not Applicable	

**Core facilities**	**Information included in the manuscript?**	**In which section is the information available?** (Reagents and Tools Table, Materials and Methods, Figures, Data Availability Section)
If your work benefited from core facilities, was their service mentioned in the acknowledgments section?	Yes	Acknoledgements, Methods

Design

Study protocol	Information included in the manuscript?	In which section is the information available? (Reagents and Tools Table, Materials and Methods, Figures, Data Availability Section)
If study protocol has been **pre-registered, provide DOI in the manuscript**. For clinical trials, provide the trial registration number **OR** cite DOI.	Not Applicable	
Report the **clinical trial registration number** (at ClinicalTrials.gov or equivalent), where applicable.	Not Applicable	

Laboratory protocol	Information included in the manuscript?	In which section is the information available? (Reagents and Tools Table, Materials and Methods, Figures, Data Availability Section)
Provide DOI OR other citation details if **external detailed step-by-step protocols** are available.	Not Applicable	

Experimental study design and statistics	Information included in the manuscript?	In which section is the information available? (Reagents and Tools Table, Materials and Methods, Figures, Data Availability Section)
Include a statement about **sample size** estimate even if no statistical methods were used.	Yes	Materials and Methods
Were any steps taken to minimize the effects of subjective bias when allocating animals/samples to treatment (e.g. **randomization procedure**)? If yes, have they been described?	Yes	Materials and Methods
Include a statement about **blinding** even if no blinding was done.	Yes	Materials and Methods
Describe **inclusion/exclusion criteria** if samples or animals were excluded from the analysis. Were the criteria pre-established?<br><br>If sample or data points were omitted from analysis, report if this was due to attrition or intentional exclusion and provide justification.	Yes	Materials and Methods
For every figure, are **statistical tests** justified as appropriate? Do the data meet the assumptions of the tests (e.g., normal distribution)? Describe any methods used to assess it. Is there an estimate of variation within each group of data? Is the variance similar between the groups that are being statistically compared?	Yes	Materials and Methods

Sample definition and in-laboratory replication	Information included in the manuscript?	In which section is the information available? (Reagents and Tools Table, Materials and Methods, Figures, Data Availability Section)
In the figure legends: state number of times the experiment was **replicated** in laboratory.	Yes	Figure legends
In the figure legends: define whether data describe **technical or biological replicates**.	Yes	Biological replicates when not clearly stated

Ethics

Ethics	Information included in the manuscript?	In which section is the information available? (Reagents and Tools Table, Materials and Methods, Figures, Data Availability Section)
Studies involving **human participants**: State details of **authority granting ethics approval** (IRB or equivalent committee(s), provide reference number for approval.	Not Applicable	
Studies involving **human participants**: Include a statement confirming that **informed consent** was obtained from all subjects and that the experiments conformed to the principles set out in the WMA Declaration of Helsinki and the Department of Health and Human Services Belmont Report.	Not Applicable	
Studies involving **human participants:** For publication of **patient photos**, include a statement confirming that consent to publish was obtained.	Not Applicable	
Studies involving experimental **animals**: State details of **authority granting ethics approval** (IRB or equivalent committee(s), provide reference number for approval. Include a statement of compliance with ethical regulations.	Yes	Msterial and Methods
Studies involving **specimen and field samples:** State if relevant **permits** obtained, provide details of authority approving study; if none were required, explain why.	Not Applicable	

Dual Use Research of Concern (DURC)	Information included in the manuscript?	In which section is the information available? (Reagents and Tools Table, Materials and Methods, Figures, Data Availability Section)
Could your study fall under dual use research restrictions? Please check biosecurity documents and list of **select agents and toxins** (CDC): https://www.selectagents.gov/sat/list.htm	Not Applicable	
If you used a select agent, is the security level of the lab appropriate and reported in the manuscript?	Not Applicable	
If a study is subject to dual use research of concern regulations, is the name of the **authority granting approval and reference number** for the regulatory approval provided in the manuscript?	Not Applicable	

Reporting

The MDAR framework recommends adoption of discipline-specific guidelines, established and endorsed through community initiatives. Journals have their own policy about requiring specific guidelines and recommendations to complement MDAR.

Adherence to community standards	Information included in the manuscript?	In which section is the information available? (Reagents and Tools Table, Materials and Methods, Figures, Data Availability Section)
State if relevant guidelines or checklists (e.g., **ICMJE, MIBBI, ARRIVE, PRISMA**) have been followed or provided.	Not Applicable	
For **tumor marker prognostic studies,** we recommend that you follow the **REMARK** reporting guidelines (see link list at top right). See author guidelines, under 'Reporting Guidelines'. Please confirm you have followed these guidelines.	Not Applicable	
For **phase II and III randomized controlled trials**, please refer to the **CONSORT** flow diagram (see link list at top right) and submit the CONSORT checklist (see link list at top right) with your submission. See author guidelines, under 'Reporting Guidelines'. Please confirm you have submitted this list.	Not Applicable	

Data Availability

Data availability	Information included in the manuscript?	In which section is the information available? (Reagents and Tools Table, Materials and Methods, Figures, Data Availability Section)
Have **primary datasets** been deposited according to the journal's guidelines (see 'Data Deposition' section) and the respective accession numbers provided in the Data Availability Section?	Yes	Methods - Data availability
Were **human clinical and genomic datasets** deposited in a public access-controlled repository in accordance to ethical obligations to the patients and to the applicable consent agreement?	Yes	Comparison of RNA-seq and microarray-based models for clinical endpoint prediction. Genome Biol 16, 133 (2015) - described in Materials and Methods
Are **computational models** that are central and integral to a study available without restrictions in a machine-readable form? Were the relevant accession numbers or links provided?	Not Applicable	
If publicly available data were reused, provide the respective **data citations in the reference list**.	Yes	Results and Material and Methods
